# Evaluation of the health and healthcare system burden due to antimicrobial-resistant *Escherichia coli* infections in humans: a systematic review and meta-analysis

**DOI:** 10.1186/s13756-020-00863-x

**Published:** 2020-12-10

**Authors:** M. C. MacKinnon, J. M. Sargeant, D. L. Pearl, R. J. Reid-Smith, C. A. Carson, E. J. Parmley, S. A. McEwen

**Affiliations:** 1 Department of Population Medicine, University of Guelph, Guelph, ON Canada; 2 Food-Borne Disease and Antimicrobial Resistance Surveillance Division, Centre of Food-borne Environmental and Zoonotic Infectious Diseases, Public Health Agency of Canada, Guelph, ON Canada; 3 Centre for Public Health and Zoonoses, University of Guelph, Guelph, ON Canada

**Keywords:** *Escherichia coli*, Burden of disease, Antimicrobial resistance, Systematic review, Meta-analysis, Multidrug resistance, Third-generation cephalosporin resistance, Quinolone resistance, Mortality, Length of hospital stay

## Abstract

**Background:**

Assessment of the burden of disease due to antimicrobial-resistant *Escherichia coli* infections facilitates understanding the scale of the problem and potential impacts, and comparison to other diseases, which allows prioritization of research, surveillance, and funding. Using systematic review and meta-analysis methodology, the objectives were to evaluate whether humans with antimicrobial-resistant *E. coli* infections experience increases in measures of health or healthcare system burden when compared to susceptible *E. coli* infections.

**Methods:**

Comprehensive literature searches were performed in four primary and seven grey literature databases. Analytic observational studies of human *E. coli* infections that assessed the impact of resistance to third/fourth/fifth-generation cephalosporins, resistance to quinolones, and/or multidrug resistance on mortality, treatment failure, length of hospital stay and/or healthcare costs were included. Two researchers independently performed screening, data extraction, and risk of bias assessment. When possible, random effect meta-analyses followed by assessment of the confidence in the cumulative evidence were performed for mortality and length of hospital stay outcomes, and narrative syntheses were performed for treatment failure and healthcare costs.

**Results:**

Literature searches identified 14,759 de-duplicated records and 76 articles were included. Based on 30-day and all-cause mortality meta-analyses, regardless of the type of resistance, there was a significant increase in the odds of dying with resistant *E. coli* infections compared to susceptible infections. A summary mean difference was not presented for total length of hospital stay meta-analyses due to substantial to considerable heterogeneity. Since small numbers of studies contributed to meta-analyses for bacterium-attributable mortality and post-infection length of hospital stay, the summary results should be considered with caution. Studies contributing results for treatment failure and healthcare costs had considerable variability in definitions and reporting.

**Conclusions:**

Overall, resistant *E. coli* infections were associated with significant 30-day and all-cause mortality burden. More research and/or improved reporting are necessary to facilitate quantitative syntheses of bacterium-attributable mortality, length of hospital stay, and hospital costs.

*Protocol Registration* PROSPERO CRD42018111197.

## Background

Antimicrobial resistance (AMR) is an exceedingly important global public health problem that is jeopardizing the advances made by modern medicine [1, 2]. A report commissioned by the Prime Minister of the United Kingdom and chaired by Lord O’Neill of Gatley predicted that by 2050, 10 million people will die each year due to antimicrobial-resistant infections [3]. In humans, *Escherichia coli* can be a commensal or pathogenic organism. It is a common cause of a variety of community- and hospital-onset infections and it is the most common cause of human blood stream infections (BSI) [4, 5]. In *E. coli,* both resistance to critically important antimicrobials such as third/fourth/fifth-generation cephalosporins and quinolones, and multidrug resistance (MDR) are recognized globally [1, 6, 7]. Resistance to third/fourth/fifth-generation cephalosporins and MDR in *E. coli* infections can complicate the treatment of invasive infections and lead to reliance on carbapenems, an antimicrobial class of last resort [1]. Quinolone-resistant *E. coli* infections can make the treatment of complicated urinary tract infections more difficult [1].

To fully understand the impact of antimicrobial-resistant *E. coli* infections in people, different aspects of the burden of disease must be analyzed. One aspect of burden can be captured by quantifying the incidence rate of antimicrobial-resistant *E. coli* infections using studies with population-based designs. This approach provides information on the absolute amount of antimicrobial-resistant *E. coli* infections and after standardization, facilitates comparison between different types of antimicrobial-resistant *E. coli* infections and different human populations. When the same type of quantification is available for other bacterial species then they can be compared to the standardized incidence rates of antimicrobial-resistant *E. coli* infections. Another aspect of burden can be explored by assessing the impact of antimicrobial-resistant *E. coli* infections on patient and healthcare outcomes [8]. Burden from the patient perspective (health burden) is commonly described using measures of mortality or morbidity, whereas, the burden from the payer and provider perspective (healthcare system burden) is commonly described using length of hospital stay and healthcare costs [8]. Finally, burden from the societal perspective can be described through quantification of the excess costs, lost productivity, and summary measures of population health including DALYs (disability-adjusted life years) and QALYs (quality-adjusted life years) [8, 9]. Recently, DALYs were estimated for third-generation cephalosporin *E. coli* infections in Europe; this was the first comprehensive assessment of the societal burden from antimicrobial-resistant *E. coli* infections [10]. Assessment of the burden of disease due to antimicrobial-resistant *E. coli* infections facilitates understanding the scale of the problem and potential impacts, and comparison to other diseases, which allows prioritization of research, surveillance, and funding.

The World Health Organization (WHO) released a global report on antimicrobial resistance surveillance in 2014 and it included a systematic review and meta-analysis addressing the health and healthcare system burden from third-generation cephalosporin and fluoroquinolone resistance in human *E. coli* infections [1]. The WHO systematic review and meta-analyses found that, compared to susceptible *E. coli* infections, third-generation cephalosporin-resistant infections had a significant twofold increase in risk using all three mortality measures (all-cause, bacterium-attributable, 30-day mortality) [1]. Moreover, with fluoroquinolone-resistant *E. coli* infections, a significant twofold increase in risk of mortality (all-cause, 30-day mortality) was also identified with the meta-analyses [1]. The literature searches for the WHO systematic review were performed in March 2013 [1]; therefore, the current review was undertaken to incorporate relevant literature published since 2013 into a current and comprehensive systematic review and meta-analysis. Another important aspect of resistance is MDR; to our knowledge there is not a systematic review evaluating the health or healthcare system burden associated with multidrug resistant *E. coli* infections in humans.

### Objectives

Using systematic review and meta-analysis methodology, the objectives were to evaluate whether measures of health or healthcare system burden increase in humans with antimicrobial-resistant *E. coli* infections when compared to those with susceptible *E. coli* infections in analytic observational studies. The three types of AMR assessed separately included resistance to third/fourth/fifth-generation cephalosporins, resistance to quinolones, and MDR (resistance to at least three antimicrobial categories or classes).

## Methods

### Protocol and registration

A protocol for this systematic review was registered with the International Prospective Register of Systematic Reviews (PROSPERO CRD42018111197) [11]. The detailed time-stamped protocol is available as supplementary material (Additional file 1). Preferred Reporting Items for Systematic Reviews and Meta-analyses (PRISMA) guidelines were used to guide preparation of the manuscript [12, 13].

### Eligibility criteria

Any analytic observational studies published as manuscripts, reports, theses, or dissertations were included [14]. Types of study designs and publications that were excluded include descriptive observational studies, review articles, commentaries, opinion pieces, editorials, newspaper articles, books, and conference proceedings; these designs were excluded because either they do not provide primary data with a concurrent comparator group or sufficient detail for data extraction and risk of bias. Relevant controlled trials have not been performed due to the nature of the research question. Included studies evaluated *E. coli* infections (confirmed by culture) in humans of any age. Studies were excluded if they were non-human studies, exclusively evaluated bacterial infections other than *E. coli*, evaluated colonization with *E. coli* instead of infection, or evaluated *E. coli* infections that were not confirmed by culture. Infection was defined as clinical signs and symptoms linked to culture of *E. coli* from a diagnostic sample. Colonization was defined as culture of *E. coli* from a diagnostic sample without any clinical signs or symptoms. There were three types of AMR that were included as the exposures of interest. Studies had to evaluate resistance to third/fourth/fifth-generation cephalosporins or the impact of extended spectrum β-lactamases (ESBL; herein referred to as third-generation cephalosporin resistance), resistance to quinolones, or MDR [15]. Multidrug resistance was defined as combined resistance to at least three antimicrobial categories or classes [16]. Studies were excluded if they evaluated alternate types of AMR that did not meet the above criteria. An appropriate comparator group was required for a study to be included. There were two types of acceptable comparator groups, either humans with *E. coli* infections that were susceptible to the AMR type of interest in the exposure group or humans with pansusceptible *E. coli* infections. Other comparator groups, such as humans with *E. coli* infections that were resistant to a different antimicrobial or healthy non-infected humans led to a study being excluded. For inclusion in the systematic review, a study had to address at least one of the following four measures of burden (outcomes). For health burden, the primary outcome was mortality (including bacterium-attributable, all-cause and 30-day mortality) and the secondary outcome was treatment failure. For healthcare system burden, the primary outcome was length of hospital stay (including total LOS and post-infection LOS) and the secondary outcome was the cost to the healthcare system. If a study failed to address at least one of the outcomes above or did not contain the outcome information specific to *E. coli*, then it was excluded. Studies published with full-text available in English were included. The publication language eligibility criterion was applied during primary eligibility screening with exclusion of non-English studies, due to available resources. For the current review, literature searches were restricted to studies published after December 31st, 1998. The date restriction was based on the fact that the comprehensive literature searches without any publication date restriction performed by the WHO systematic review only identified relevant studies that were published starting in 1999 [1]. No restrictions based on country of study were applied.

### Information sources

Four literature databases were searched: MEDLINE^®^ in Ovid (including in-process and other non-indexed citations and daily—without revisions); Embase in Ovid; Web of Science Current Contents Connect in Web of Science; and Global Health in CAB Direct. Grey literature sources were searched from WHO (including Global Index Medicus), Centers for Disease Control and Prevention (CDC), European Centre for Disease Prevention and Control (ECDC), European Medicines Agency (EMA), Public Health Agency of Canada (PHAC), and Health Canada. The first 250 results sorted based on relevance from Google Scholar were also screened for eligibility. The reference lists from the included studies were reviewed to confirm saturation of the literature. The literature database searches were performed on September 17th, 2018 and the grey literature source searches were performed between September 21st and 28th, 2018.

### Search

Librarians with expertise in systematic reviews were consulted during development of the search strategy (Additional file 2). Based on the eligibility criteria, search terms related to *E. coli* (population), cephalosporins, quinolones and multidrug resistance (exposures), and the outcomes of interest were included in the search strategy. The search terms used a combination of medical subject headings (MeSH) terms and keywords. The publication date restriction from 1999 to the date the search was performed was used in the search. The search strategy for MEDLINE^®^ in Ovid is available in Additional file 2. The search strategy was modified as required for each literature database and grey literature source (additional search strategies available upon request).

### Study selection

EndNote X7 and X9 were used for citation management and duplicate removal for articles identified in the searches [17, 18]. The bibliographic citation information for all remaining articles was uploaded to DistillerSR and additional duplicates were removed [19]. DistillerSR facilitated primary screening, secondary screening, data extraction, and assessment of risk of bias [19].

Two researchers performed both primary and secondary screening independently. The answers were compared, and disagreements were discussed until consensus was achieved. If consensus was not achieved, a third researcher arbitrated. The primary screening of articles was conducted on the titles and abstracts of each article using five questions based on the eligibility criteria (Additional file 3). Prior to starting primary screening, the two researchers piloted 100 articles. The possible answers were ‘yes,’ ‘no,’ or ‘unclear.’ After consensus, if one or more answers were ‘no,’ then the article was excluded and any combination of ‘yes’ and ‘unclear’ led to an article proceeding to secondary screening. For articles proceeding to secondary screening, full text articles (PDF format) were obtained. Seven questions based on the eligibility criteria were used for secondary screening of the full text articles (Additional file 3). In deviation to the protocol, a seventh question was added for secondary screening after completion of the pilot performed on five articles. The question added was, “Does the study have outcome data specific to *E. coli* infections?” The possible answers were ‘yes’ or ‘no.’ After consensus, if one or more answers were ‘no,’ then the article was excluded and answers of ‘yes’ to all of the questions led to an article being included in the systematic review and proceeding to data extraction.

### Data collection process

Data were extracted for the characteristics of the study and study participants, and the results for the health and healthcare system outcomes. The data extraction form was piloted using five articles and finalized prior to the extraction of data for the review. Two researchers performed the data extraction independently. Their results were compared, and consensus was achieved using the methods described for primary and secondary screening. If there was insufficient detail present in the study to allow complete data extraction and the study was published within the previous 5 years, the corresponding author was contacted in an attempt to acquire the necessary data. If the study was published more than 5 years ago or the author did not respond, then as much data as possible were extracted from the study and missing data were noted.

### Data items

DistillerSR [19] documented the researcher performing the extraction, the date of extraction, the unique identifier for the article and the article citation. Related to the characteristics of the study, the following data were extracted: year of publication; type of document (e.g., peer-reviewed article, report, dissertation); author reported study design; year(s) data were collected; country or countries where study was performed; type of site for data collection (e.g., hospital, community clinic); and number of sites. The following data were extracted related to the characteristics of the study participants: underlying disease processes; definition of cases with resistant (R) infections; number of cases with R infections; definition of cases with susceptible (S) infections in comparator group; number of cases with S infections in comparator group; details of R and S group selection; mean age of R and S groups with measure of variability; distribution of sex in R and S groups; source of samples; type of infection; source/timing of participants’ infection (community-onset (community-acquired and healthcare-acquired) and hospital-onset); method used to summarize co-morbidities; method used to summarize disease severity; duration of follow-up; method for antimicrobial susceptibility testing; and minimum inhibitory concentration interpretive criteria used.

For all health and healthcare system burden outcomes, data related to the statistical analysis methods, details of adjustment for confounding, and any loss to follow-up were extracted. For the three measures of mortality (all-cause, 30-day, and bacterium-attributable), an adjusted measure of association with measure of variability was extracted from each manuscript. If an adjusted measure of association was not available, then a crude measure of association with measure of variability and/or raw data were extracted. All-cause mortality included when a patient died due to any cause with no restriction on the length of follow-up. When the cause of death was confirmed to be due to the bacterial (*E.* *coli*) infection, with no restriction on the length of follow-up, it was included as bacterium-attributable mortality. The outcome was extracted as 30-day mortality when the follow-up period was 30-days after the culture was obtained and the death was due to any cause. If a study reported 30-day mortality, those outcome data were also used for all-cause mortality. In the literature, there is not a consistent definition of treatment failure. Therefore, data related to the definition of treatment failure and associated raw data were extracted. For both measures of LOS (total LOS and post-infection LOS), the mean difference (MD) or measure of central tendency (mean or median) with measure of variability were extracted (extraction of median LOS was a deviation from the protocol). The total LOS was the number of days in hospital from admission to discharge. The post-infection LOS was the number of days in hospital from collection of the positive sample to discharge. In the literature, there is not a consistent definition of healthcare system costs. Therefore, the data extracted were a description of the components included, the cost with measure of variability for the R and S groups, and year and currency for the cost.

### Risk of bias in individual studies

Prior to risk of bias assessment, the author-reported study design for every study was verified or, if a study design was not reported, it was established. This was performed independently by two researchers and the results were compared to ensure agreement. The risk of bias assessment was performed separately for each reported outcome in every study. The Cochrane tool for Risk of Bias in Non-randomized Studies of Interventions (ROBINS-I) modified for use with exposure studies was used to assess risk of bias [20]. The five domains considered were: bias due to confounding; bias in selection of participants into the study; bias in measurement of exposures; bias due to missing data; and bias in measurement of outcomes. Dependent on individual study design and analysis, and the burden of disease measure, potentially important confounders to consider could include co-morbidities or severity of underlying disease measured at least 48 h prior to infection, length of hospital stay prior to infection, type of infection, source of bacteremia, source/timing of infection, age and sex.

The options for risk of bias in each domain of bias were low, moderate, serious, critical or no information. The most severe level of risk of bias (closest to critical) in the five domains determined the overall risk of bias for each reported outcome in every study. The domain of bias due to departures from intended intervention was not assessed because it was not relevant in the context of these exposure studies. In deviation from the protocol, the domain of bias in selection of the reported results was not assessed because none of the articles included had a pre-registered protocol or available statistical plan. Level of risk of bias was not used to determine eligibility for data synthesis.

### Summary measures

The summary measure used for mortality was the odds ration (OR) and for LOS measures was the MD. All summary measures were reported with a 95% confidence interval (CI).

### Synthesis of results

The country’s income status according to the World Bank Country Income Classification was determined for all studies [21]. Data synthesis was performed separately for studies assessing the impact of each of the three types of AMR of interest. Each type of primary outcome was synthesized separately. If outcome data for measures of mortality were extracted as raw data, then a crude OR and 95% CI was calculated to facilitate inclusion in the meta-analysis. If there were at least two studies reporting the same measure of mortality, then a random effects meta-analysis in R 3.6.2/RStudio 1.2.1335 with the package *meta* and function *metagen* was used to summarize data by reporting a summary OR (sOR) [22–24]. If the same measure of LOS was reported in at least two studies, then a random-effects meta-analysis in R 3.6.2/RStudio 1.2.1335 with the package *meta* and function *metacont* was used to report a summary MD (sMD) [22–24]. The inverse variance method and Hartung-Knapp adjustment for random effects models were used. I^2^ was used to assess heterogeneity. If substantial to considerable heterogeneity was present (I^2^ ≥ 50%), the summary measure was not presented [25]. However, in deviation from the protocol, one of our main outcomes had an I^2^ of 56.0% and the sOR was presented. The sOR was presented because it was a critical outcome and was slightly above our arbitrary cut-off for I^2^. In general, due to the small number of studies included in the meta-analyses, I^2^ was prioritized over Cochrane’s Q for the assessment of heterogeneity. If I^2^ ≥ 50% with at least three studies and sufficient variation in the source of heterogeneity, then clinical and methodological heterogeneity were explored using subgroup meta-analysis. Potentially relevant sources of clinical heterogeneity included the type of *E. coli* infection, mean age, proportion female, country income status, and the type of comparator group (pansusceptible vs. susceptible to the antimicrobial of interest). Possibly relevant sources of methodological heterogeneity included level of bias due to confounding and overall level of risk of bias. Forest plots were produced in R 3.6.2/RStudio 1.2.1335 with the package *meta* and function *forest* to visualize the results of the meta-analyses [22–24]. A narrative synthesis was used to summarize data for secondary outcomes and for primary outcomes when there was only one article reporting the outcome, where calculation of a summary measure using meta-analysis was not appropriate, or where subgroup meta-analysis was not possible.

### Risk of bias across studies

For each outcome synthesized using a meta-analysis with least 10 studies included, publication bias/small study effects were assessed using funnel plots produced in R 3.6.2/RStudio 1.2.1335 with the package metaviz and function viz_funnel [22, 23, 26].

### Additional analyses

Where meta-analyses were performed at the outcome level, the confidence in the cumulative evidence for primary outcomes was assessed using the Grading of Recommendations Assessment, Development, and Evaluation (GRADE) methodology [27, 28]. GRADE assessment was performed collaboratively by two authors. With GRADE, observational studies start at low level of confidence [28]. The criteria evaluated which could result in downgrading the quality of the evidence to very low were risk of bias (based on previously described risk of bias assessment), indirectness, inconsistency, publication bias, and imprecision [29–33]. The criteria evaluated which could result in upgrading the quality of evidence were large magnitude of effect, dose response and confounders likely minimized the effect [34]. The threshold for evidence of a large magnitude of effect were sOR ≥ 2 or sMD ≥ 5 days. GRADE summary of findings tables were prepared using GRADEpro [35]. The baseline risk was calculated from the overall risk in the antimicrobial susceptible group from the studies included in the meta-analysis for each mortality outcome. Using GRADEpro for each mortality outcome, the sOR from the meta-analysis and calculated baseline risk were used to estimate the risk difference, which was presented as the absolute effect in the GRADE summary of findings table [36].

## Results

### Study selection

**Fig. 1:**
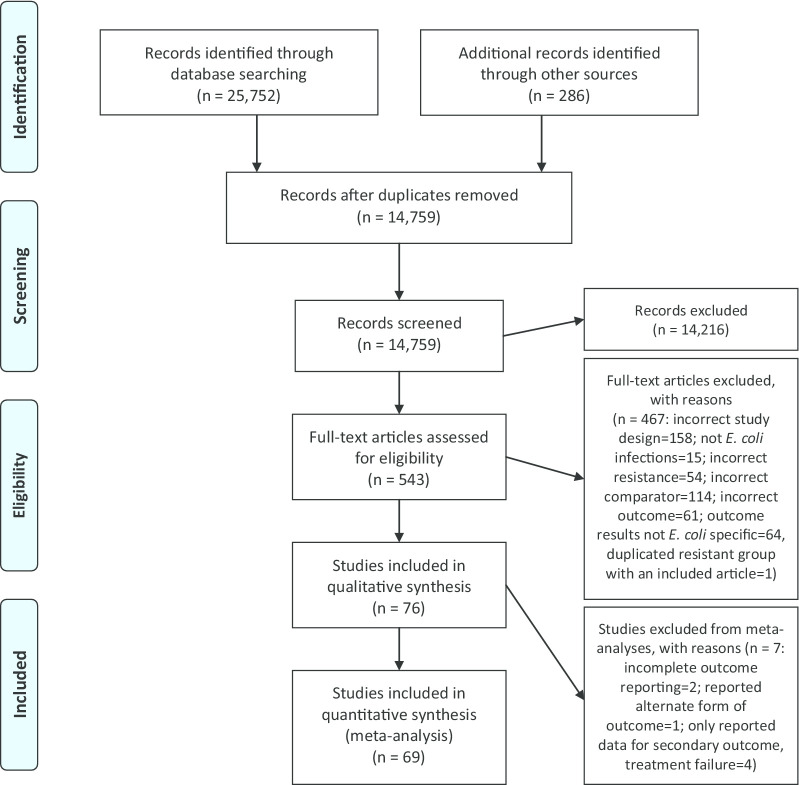
PRISMA Flow Diagram demonstrating progression of articles through identification, screening and synthesis for systematic review

After duplicates were removed, there were 14,759 records for primary screening of the title and abstract (Fig. 1) [12]. There were 14,216 records excluded during primary screening, which included 13 records with full-text articles not published in English (5 published in Spanish; 3 in Chinese; 2 in Turkish; 1 in Korean; 1 in French; and 1 in German). These non-English articles were deemed potentially relevant based on screening of their title and abstract and would have proceeded to secondary screening if full-text was available in English. Secondary screening of full-text articles was performed on 543 articles and 76 articles that met the inclusion criteria were included in the systematic review (Additional file 4). No additional potentially relevant articles were identified when the reference lists of the articles included in the systematic review were reviewed.

### Study characteristics

#### Methods of included study

Detailed individual study and participant characteristics are available in Additional files 5 and 6. In the context of the research question for this systematic review, the studies were all cohort study designs. Using the World Bank Income Status classification, there were four studies performed in lower-middle income countries (5.3%) [37–40], eight studies from upper-middle income countries (10.5%) [41–48], one multinational study that included a combination of upper-middle and high income countries (1.3%) [49], and 63 studies from high income countries (82.9%). Included studies were performed in Europe, Asia, North America, and Oceania. There were no studies from Africa or South America. All of the studies were hospital or laboratory-based. The majority of studies (57, 75%) collected data from a single site, and 25% of studies (19 studies) collected data from at least two sites, which included 3 national studies [50–52], and 2 multi-national studies [49, 53]. Most studies included patients with both community-onset and hospital-onset *E. coli* infections (35 studies, 46.1%) or only community-onset *E. coli* infection (19 studies, 25.0%). Five studies (6.6%) included only hospital-onset *E. coli* infections [48, 53–56], and 17 studies (22.4%) did not report the timing of the *E.* coli infections. Studies included different types of *E. coli* infections, however, the majority of studies (46, 60.5%) only included *E. coli* BSI.

#### Exposure and comparator

Of the 76 studies included, 96.1% provided outcome data related to a single or two types of AMR of interest to this review (69 studies and 4 studies [50, 57–59], respectively), and 3.9% (3 studies) addressed all three types of AMR of interest [41, 60, 61], There were 57 studies (75.0%) that provided outcome data related to third-generation cephalosporin resistance, 21 studies (27.6%) that provided outcome data related to quinolone resistance, and 8 studies (10.5%) that provided multidrug resistant outcome data [39, 41, 45, 55, 60–63]. The total percentages for types of AMR reported are more than 100% since 7 studies reported outcome data for two or three types of resistance. Five of the MDR studies (62.5%) [45, 55, 61–63] defined MDR using the proposed standardized definition from Magiorakos et al. in 2012 [16]. The other three MDR studies defined MDR as an isolate that was resistant to at least one antimicrobial in three or more antimicrobial classes and one of these [60] was published prior to the proposed standardized MDR definition [16]. The comparator group in all studies included patients with *E. coli* infections that were susceptible to the defined antimicrobial of interest; there were no studies with pansusceptible comparator groups.

#### Population

Studies that addressed third-generation cephalosporin resistance included 6192 third-generation cephalosporin-resistant *E. coli* infections out of a total of 42,543 third-generation cephalosporin-resistant and third-generation cephalosporin-susceptible *E. coli* infections. Studies that addressed quinolone resistance included 7556 quinolone-resistant *E. coli* infections out of a total of 40,054 quinolone-resistant and quinolone-susceptible *E. coli* infections. Studies that addressed MDR included 3171 multidrug-resistant *E. coli* infections out of a total of 7632 multidrug-resistant and non-multidrug-resistant *E. coli* infections. Most studies (48, 63.2%) did not have selection criteria related to resistant and susceptible infections, instead they enrolled all patients with the specified type of *E. coli* infection during the study period. The second most common approach to selection used in the studies was enrolment of all patients with resistant *E. coli* infections during the study period and matching to a given number of patients with susceptible *E. coli* infections (24, 31.2%). The criteria used for matching varied between studies. Almost all of the studies either did not have age restriction for sampling (43, 56.6%) or enrolled adults and elderly patients (30, 39.5%). Both, the enrolment criteria based on sex and the reporting of the sex included varied between studies, and therefore it is difficult to provide a generalized statement regarding sex.

#### Outcomes

The studies included reported between one and four burden of disease measures (outcomes). The most frequently and consistently reported burden of disease measures were all-cause mortality, followed by 30-day mortality and treatment failure (Table 1). Bacterium-attributable mortality, hospital costs and post-infection LOS were reported least frequently and inconsistently (Table 1).

**Table 1:** Number of studies in qualitative synthesis and meta-analysis for each burden of disease measure by AMR type

Burden of disease measure (outcome)	Number of studies in the qualitative synthesis (number of studies included in the meta-analysis)		
	Third-generation cephalosporin resistance	Quinolone resistance	Multidrug resistance
30-day mortality	23 (23)	9 (8)	4 (4)
All-cause mortality	51 (51)	17 (16)	5 (5)
Bacterial-attributable mortality	3 (3)	0 (0)	0 (0)
Treatment failure	15 (n/a)	7 (n/a)	2 (n/a)
Total LOS	13 (5)	4 (3)	1 (0)
Post-infection LOS	7 (2)	0 (0)	0 (0)
Hospital costs	4 (n/a)	0 (n/a)	2 (n/a)

LOS, length of hospital stay; n/a, not applicable, meta-analysis not planned as per protocol; AMR, antimicrobial resistance

### Risk of bias within studies

Summaries of the risk of bias analysis using ROBINS-I for each combination of type of AMR and burden of disease outcome are available in Additional file 7. In general, across all types of resistance and burden of disease outcomes, moderate and serious were the most common overall risk of bias levels assigned to the studies. The confounding domain of bias generally had the most influence on the overall risk of bias level; it was the same level as the overall risk of bias in 96.4% of study level assessments (161/167).

## Results of individual studies, synthesis of results, risk of bias across studies, and additional analyses

### 30-day mortality

#### Third-generation cephalosporin resistance

**Fig. 2:**
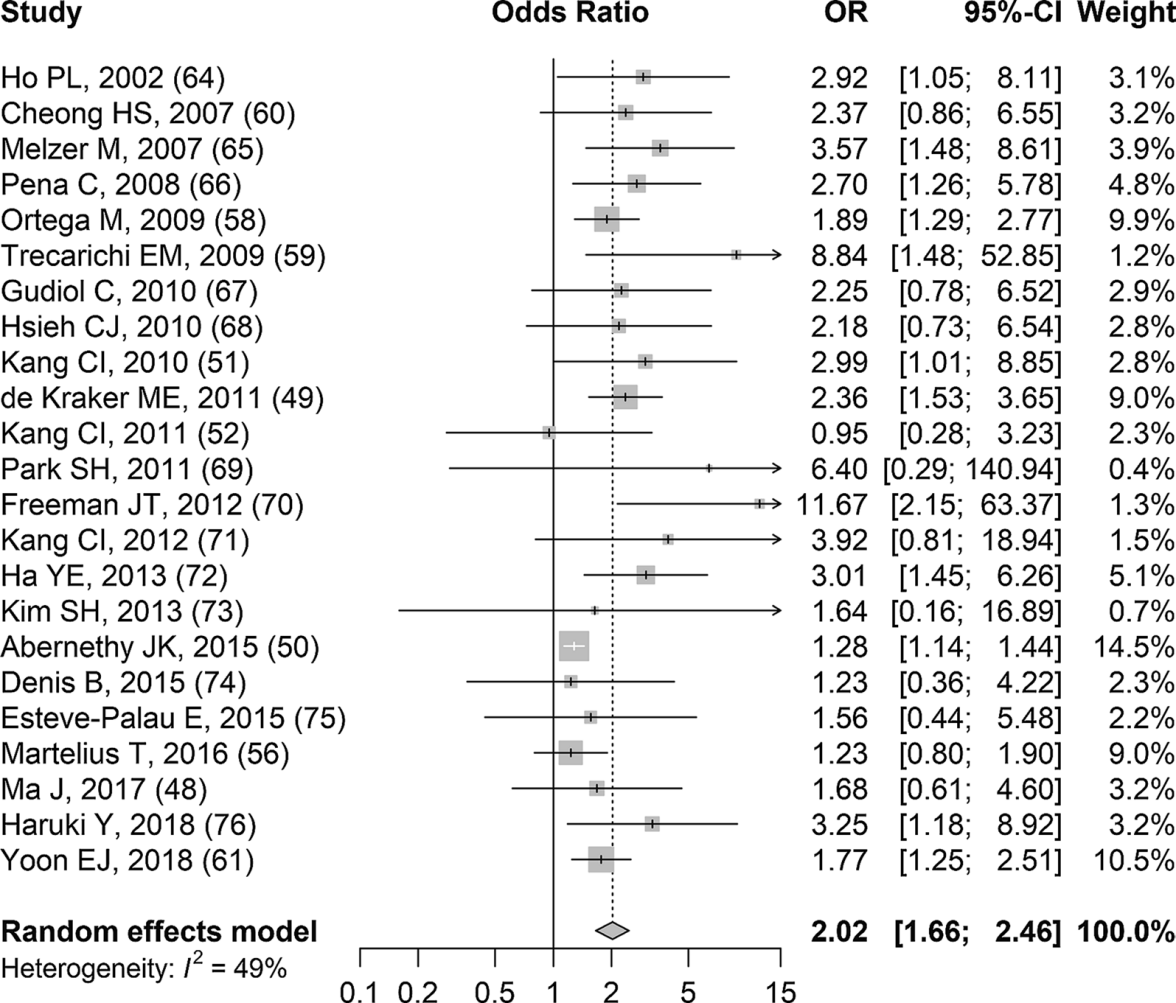
Forest plot of random-effects meta-analysis assessing impact of third-generation cephalosporin-resistant *E. coli* infections on 30-day mortality

Results related to 30-day mortality among third-generation cephalosporin-resistant *E. coli* infections were reported in 23 studies and the detailed study-level results are available in Additional file 8a [48–52, 56, 58–61, 64–76]. Based on random effects meta-analysis of 23 studies, patients with third-generation cephalosporin-resistant *E. coli* infections had significantly increased odds of dying within 30 days of the onset of their infection compared to patients with third-generation cephalosporin-susceptible *E. coli* infections (sOR 2.02, 95% CI 1.66–2.46, *p* < 0.001; Fig. 2). The results from the studies were relatively consistent and there was moderate heterogeneity (I^2^ 49.3%). The funnel plot had evidence of publication bias/small study effects because there was an absence of smaller studies with null associations or associations less than one (located in the lower left corner of the funnel plot; Additional file 9). However, there were few small studies in general. Due to the strong measure of association from the meta-analysis, the confidence in the evidence was moderate based on GRADE (Table 2).

**Table 2:** Summary of findings for mortality outcomes [35]

Burden of disease measure	Type of antimicrobial resistance	Number of participants (studies^a^)	Relative effect sOR (95% CI)	Absolute effect Risk difference (95% CI)	Certainty of the evidence (GRADE)^b^	Comment^c^
30-day mortality	Third-generation cephalosporin	31,934 (23 studies)	2.02 (1.66–2.46)	112 more deaths per 1000 (from 76 to 151 more)		Evidence to support upgrading due to strong association and no evidence to support downgrading
	Quinolone	27,703 (8 studies)	1.49 (1.23–1.82)	58 more deaths per 1000 (from 28 to 93 more)		No evidence to support downgrading or upgrading
	MDR	6506 (4 studies)	1.63 (1.54–1.71)	96 more deaths per 1000 (from 83 to 106 more)		No evidence to support downgrading or upgrading
All-cause mortality	Third-generation cephalosporin	40,623 (51 studies)	2.27 (1.92–2.70)	130 more deaths per 1000 (from 98 to 166 more)		Evidence to support upgrading due to strong association and no evidence to support downgrading
	Quinolone	31,324 (16 studies)	1.72 (1.40–2.12)	82 more deaths per 1000 (from 48 to 121 more)^d^		No evidence to support downgrading or upgrading
	MDR	6814 (5 studies)	1.63 (1.55–1.70)	92 more deaths per 1000 (from 81 to 100 more)		No evidence to support downgrading or upgrading
Bacterium-attributable mortality	Third-generation cephalosporin	327 (3 studies)	1.76 (0.84–3.70)	78 more deaths per 1000 (from 18 fewer to 225 more)		Downgraded due to serious inconsistency and imprecision. No evidence to support upgrading.
	Quinolone	–	–	–	–	Not reported
	MDR	–	–	–	–	Not reported

sOR, summary odds ratio

^a^All studies were observational

^b^GRADE assessment began at low instead of high, since studies were observational

^c^Details of GRADE assessment available in Additional file 23

^d^Raw data not available from one study and therefore did not contribute to calculation of baseline risk

#### Quinolone resistance

**Fig. 3:**
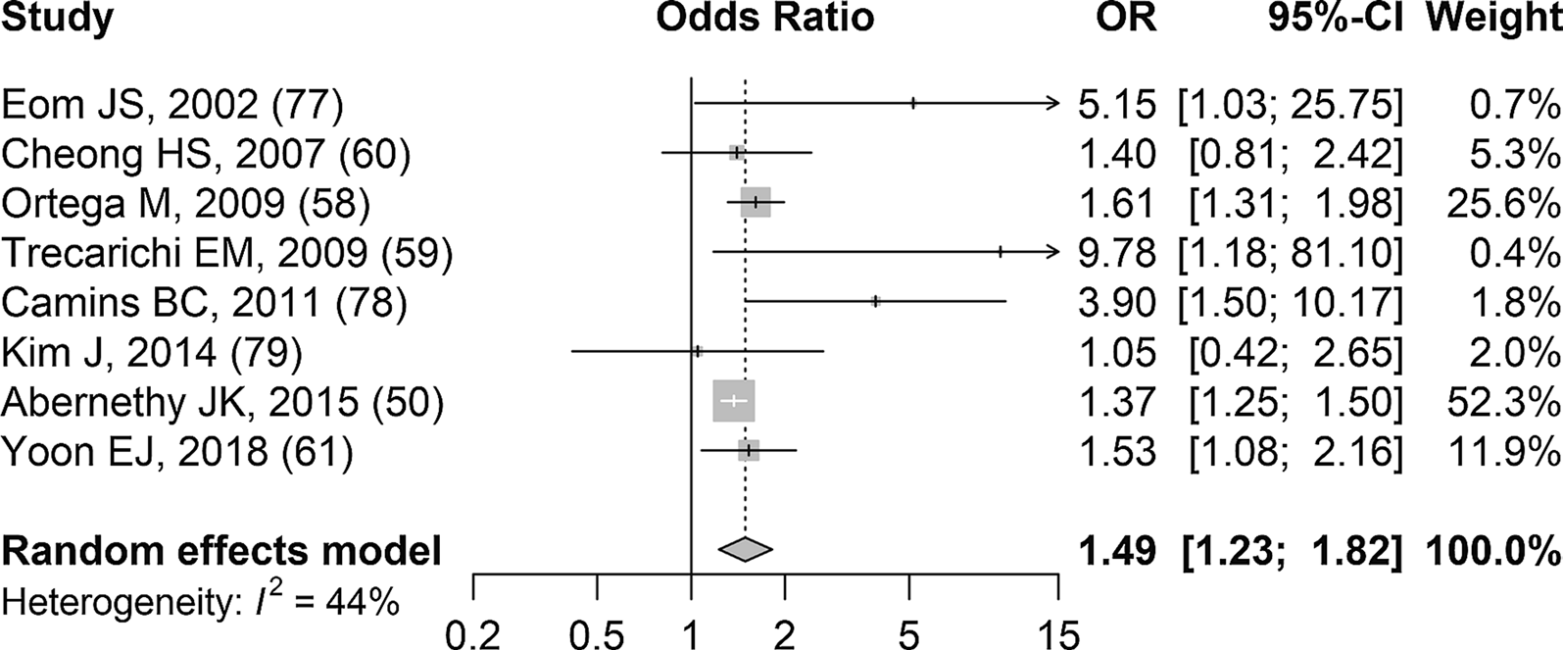
Forest plot of random-effects meta-analysis assessing impact of quinolone-resistant *E. coli* infections on 30-day mortality

Results related to 30-day mortality among quinolone-resistant *E. coli* infections were reported in nine studies and the detailed study-level results are available in Additional file 8b [50, 54, 58–61, 77–79]. The results from one study could not be included in the meta-analysis (details in Additional file 8b) [54]. Based on random effects meta-analysis of eight studies, compared to patients with quinolone-susceptible *E. coli* infections those with quinolone-resistant *E. coli* infections had significantly increased odds of dying within 30 days of the onset of their infection (sOR 1.49, 95% CI 1.23–1.82, *p* = 0.002; Fig. 3). The results from the studies were relatively consistent and there was moderate heterogeneity (I^2^ 44.1%). The confidence in the evidence was low, based on GRADE (Table 2).

#### Multidrug resistance

Results related to 30-day mortality and multidrug-resistant *E. coli* infections were reported in four studies and the detailed study-level results are available in Additional file 8c [39, 45, 60, 61]. Based on random effects meta-analysis of four studies, patients with multidrug-resistant *E. coli* infections had significantly higher odds of dying within 30 days of the onset of their infection compared to patients with non-multidrug resistant *E. coli* infections (sOR 1.63, 95% CI 1.54–1.71, *p* < 0.001; Additional file 10). The results from the studies were very consistent and there was no heterogeneity (I^2^ 0.0%). GRADE assessment revealed the confidence in the evidence was low (Table 2).

### All-cause mortality

#### Third-generation cephalosporin resistance

**Fig. 4:**
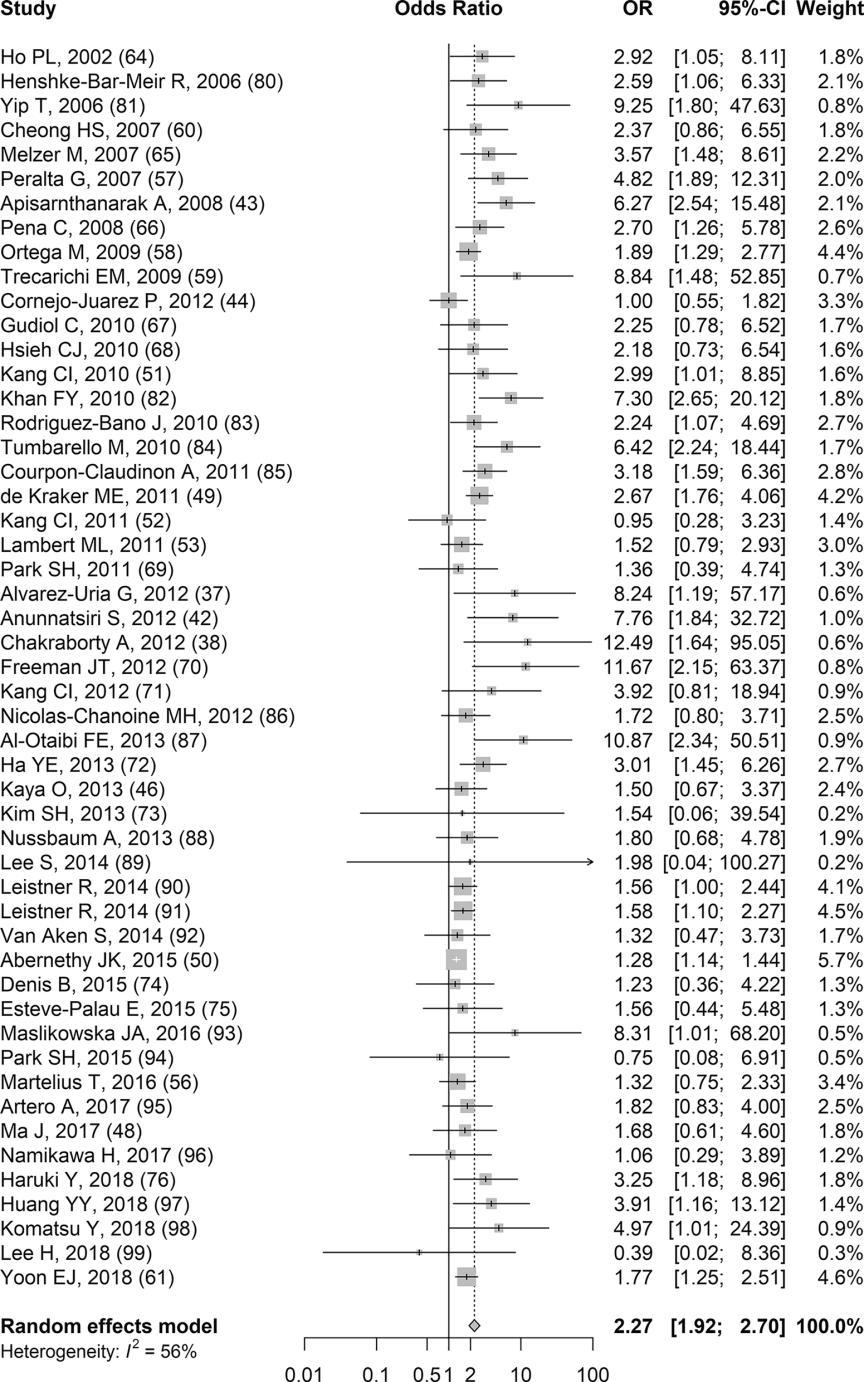
Forest plot of random-effects meta-analysis assessing impact of third-generation cephalosporin-resistant *E. coli* infections on all-cause mortality

Results related to all-cause mortality and third-generation cephalosporin-resistant *E. coli* infections were reported in 51 studies (Additional file 11a) and all of the studies were included in the random effects meta-analysis [37, 38, 42–44, 46, 48–53, 56–61, 64–76, 80–99]. Based on the sOR, patients with third-generation cephalosporin-resistant *E. coli* infections had significantly higher odds of dying from any cause compared to patients with third-generation cephalosporin-susceptible *E. coli* infections (sOR 2.27, 95% CI 1.92–2.70, *p* < 0.001; Fig. 4). The results from the studies were relatively consistent and there was moderate to substantial heterogeneity (I^2^ 56.0%). Subgroup meta-analyses were unable to determine a possible explanation for the moderate to substantial heterogeneity. The possible sources that were explored, but did not explain the heterogeneity, were the type of *E. coli* infection, whether the study defined exposure positive as ESBL *E. coli* or more broadly as third-generation cephalosporin-resistant *E. coli*, the timing of the *E. coli* infection, the number of sites included in the study, the definition used for all-cause mortality, the level of confounding bias in the study, and the overall risk of bias in the study. The funnel plot was relatively symmetric but there were few small studies in general (Additional file 12). There were several small studies in the lower left corner but an absence of small studies in the lower right corner. The confidence in the evidence was moderate with GRADE and this was due to the strong association from the meta-analysis (Table 2).

#### Quinolone resistance

**Fig. 5:**
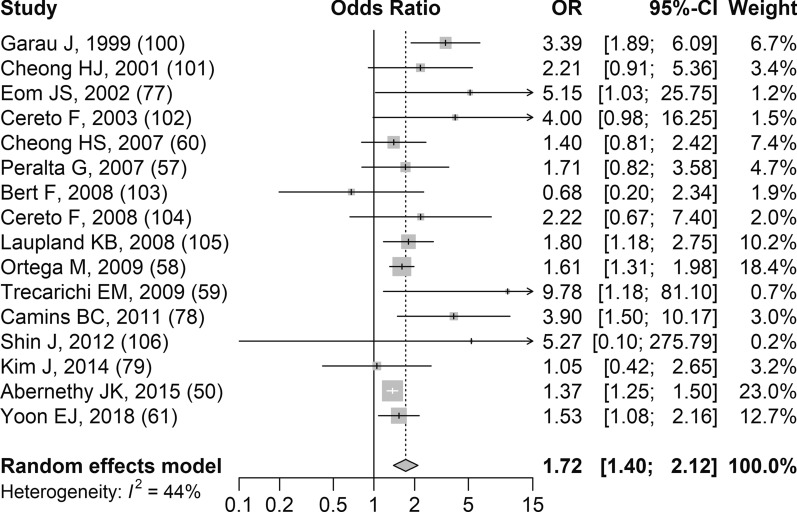
Forest plot of random-effects meta-analysis assessing impact of quinolone-resistant *E. coli* infections on all-cause mortality

There were 17 studies that reported results related to quinolone-resistant *E. coli* infections and all-cause mortality and the detailed study-level results are available in Additional file 11b [50, 54, 57–61, 77–79, 100–106]. One study could not be included in the meta-analysis (details in Additional file 11b) [54]. Based on random effects meta-analysis of 16 studies, compared to patients with quinolone-susceptible *E. coli* infections those with quinolone-resistant *E. coli* infections had significantly higher odds of dying (sOR 1.72, 95% CI 1.40–2.12, *p* < 0.001; Fig. 5). The results from the studies were relatively consistent and there was moderate heterogeneity (I^2^ 44.5%). There was evidence of publication bias/small study effects based on the funnel plot (Additional file 13). There were few small studies overall and an absence of small studies in the lower left corner of the funnel plot. GRADE assessment revealed the confidence in the evidence was low (Table 2).

#### Multidrug resistance

Results related to multidrug-resistant *E. coli* infections and all-cause mortality were reported in five studies and all five studies were included in the random effects meta-analysis (Additional file 11c) [39, 45, 60–62]. Based on random effects meta-analysis of five studies, patients with multidrug-resistant *E. coli* infections had significantly higher odds of dying compared to patients with non-multidrug-resistant *E. coli* infections (sOR 1.63, 95% CI 1.55–1.70, *p* < 0.001; Additional file 14). The results from the studies were very consistent and there was no heterogeneity (I^2^ 0.0%). The confidence in evidence was low based on GRADE assessment (Table 2).

### Bacterium-attributable mortality

#### Third-generation cephalosporin resistance

Results related to bacterium-attributable mortality and third-generation cephalosporin-resistant *E. coli* infections were reported in three studies and the detailed study-level results are available in Additional file 15 [44, 47, 93]. Random effects meta-analysis of three studies demonstrated a non-significant summary odds ratio (sOR 1.76, 95% CI 0.84–3.70, *p* = 0.082; Additional file 16). The results from the studies were very consistent and there was no heterogeneity (I^2^ 0.0%). Due to strong imprecision and inconsistency, the confidence in the evidence was very low based on GRADE assessment (Table 2).

### Treatment failure

#### Third-generation cephalosporin resistance

Results related to treatment failure and third-generation cephalosporin resistance were reported in 15 studies and the detailed results are available in Additional file 17a. Clinical treatment failure was reported in 14 studies [38, 40–42, 47, 51, 75, 81, 84, 93, 94, 107–109] and microbiological treatment failure was reported in 4 studies [40, 89, 93, 94]. The common elements included in the definitions of clinical treatment failure included persistence of clinical signs, or absence of improvement or resolution (9 studies [40–42, 51, 75, 84, 93, 107, 108]), requirement of a second antimicrobial prescription (5 studies [41, 75, 107–109]), and discontinuation of peritoneal dialysis (2 studies [47, 81]). Death was explicitly included as clinical treatment failure in seven studies [38, 47, 51, 75, 81, 84, 94], and one study excluded deaths [42] (within 72 h) from treatment failure analysis. The timeframes considered for clinical treatment failure were variable. Seven studies assessed clinical treatment failure with 3–14 days after starting antimicrobial therapy [41, 42, 51, 75, 84, 107–109], three studies assessed it within 2–4 weeks after completion of antimicrobial therapy [93, 94, 107], and four studies did not provide a timeframe [38, 40, 47, 81]. Related to clinical treatment failure, eight studies reported a significant increase with third-generation cephalosporin-resistant *E. coli* infections [41, 42, 75, 81, 84, 107–109], two studies reported a non-significant increase with resistant infections [51, 93], two studies did not statistically test the difference between the resistant and susceptible groups [38, 40] and two studies reported a non-significant increase with susceptible infections [47, 94]. Microbiological treatment failure definitions were based on bacterial growth on follow up culture and the length of follow up ranged from 3 days after starting antimicrobial therapy to 4 weeks after completing antimicrobial therapy.

#### Quinolone resistance

Treatment failure results for quinolone resistance were reported in seven studies (Additional file 17b). Clinical treatment failure was reported in all 7 studies [41, 77, 78, 104, 106, 110, 111] and one study also reported microbiological treatment failure [110]. The definitions used for clinical treatment failure included these common elements: persistence, worsening or recurrence of clinical signs (6 studies [41, 77, 78, 104, 106, 110]), and microbiological treatment failure (positive growth on follow-up culture, studies [77, 78, 111]). The timeframe for the assessment of clinical treatment failure varied from 3 days to 30 days after starting antimicrobial therapy to 10 days to 2 weeks after completing antimicrobial therapy. Related to clinical treatment failure, five studies reported a significant increase with quinolone-resistant *E. coli* infections [41, 77, 78, 106, 111], and two studies reported a non-significant increase with resistant infections [104, 110]. The study that specifically addressed microbiological treatment failure defined it as, “identification of infection with a new pathogen or persistence of the original pathogen from culture during a follow-up visit” [110].

#### Multidrug resistance

Two studies reported results for treatment failure and MDR (Additional file 17c) [41, 62]. Both of the studies reported clinical treatment failure. One study reported a significant increase in clinical treatment failure with multidrug-resistant *E. coli* infections [41], and one study reported a non-significant increase with multidrug-resistant infections [62].

### Total length of hospital stay

#### Third-generation cephalosporin resistance

Results related to total LOS and third-generation cephalosporin-resistant *E. coli* infections were reported in 13 studies (Additional file 18a). However, only five studies could be included in the meta-analysis; they reported total LOS results as a mean with standard deviation [40, 75, 89, 92, 95]. Eight studies could not be included in the meta-analysis because they reported total LOS using a median and interquartile range [74, 90, 94, 97, 99], or did not consistently report both a measure of central tendency and variability [43, 88, 108]. The five included studies reported an increase in the mean LOS in the third-generation cephalosporin-resistant patients compared to third-generation cephalosporin-susceptible patients, and the mean difference was significant in four of the five studies. The results were not consistent between studies with the magnitude of the mean difference being considerably larger in one of the studies (Additional file 19). The random effects meta-analysis had considerable heterogeneity and therefore the sMD is not reported (I^2^ 97.0%). An explanation for the considerable heterogeneity was not discovered using subgroup meta-analysis. The possible sources explored included type of infection, percentage of females in the study, World Bank income status of the country, timing of infection, and number of sites included in the study. Due to serious imprecision and inconsistency, the confidence in the evidence was very low with GRADE assessment (Table 3).

**Table 3:** Summary of findings for length of hospital stay outcomes [35]

Burden of disease measure	Type of antimicrobial resistance	Number of participants (studies^a^)	Absolute effect, sMD (95% CI)	Certainty of the evidence (GRADE)^b^	Comment^c^
Total LOS	Third-generation cephalosporin	888 (5 studies)	sMD not calculated due to considerable heterogeneity		Downgraded due to serious inconsistency and imprecision
	Quinolone	646 (8 studies)	sMD not calculated due to substantial to considerable heterogeneity		Downgraded due to serious inconsistency and imprecision
	MDR	–	–	–	Only reported in 1 study, meta-analysis and GRADE assessment not performed
Post-infection LOS	Third-generation cephalosporin	538 (2 studies)	7.16 days higher (2.76 higher to 11.57 higher)		Downgraded due to serious imprecision. Evidence to support upgrading outweighed by evidence to support downgrading.
	Quinolone	–	–	–	Not reported
	MDR	–	–	–	Not reported

LOS, length of hospital stay; sMD, summary mean difference

^a^All studies were observational

^b^GRADE assessment began at low instead of high, since studies were observational

^c^Details of GRADE assessment available in Additional file 23

#### Quinolone resistance

Results related to total LOS and quinolone-resistant *E. coli* infections were reported in four studies (Additional file 18b) [54, 78, 106, 110]. Three studies provided the data required for inclusion in the meta-analysis [78, 106, 110]. An increase in the mean LOS in the quinolone-resistant patients compared to quinolone-susceptible patients was reported in all three studies, but the mean difference was only significant in two of the three studies. The magnitude of the mean difference was larger in one of the studies compared to the other two studies (Additional file 20). The random effects meta-analysis had substantial to considerable heterogeneity so the sMD is not reported (I^2^ 78.3%). Subgroup meta-analysis did not provide insight into the reasons for the substantial to considerable heterogeneity. The percentage of females in the study, number of sites included in the study, and level of confounding bias or overall risk of bias were explored as possible sources of heterogeneity. The confidence in the evidence based on GRADE assessment was very low and this was due to serious imprecision and inconsistency (Table 3).

#### Multidrug resistance

Only one study reported results for total LOS and multidrug-resistant *E. coli* infections (Additional file 18c) [62]. The LOS results were reported as medians and interquartile range (IQR) for each group and there was a non-significant increase in the median LOS for the multidrug-resistant infection group.

### Post-infection length of hospital stay

#### Third-generation cephalosporin resistance

**Fig. 6:**
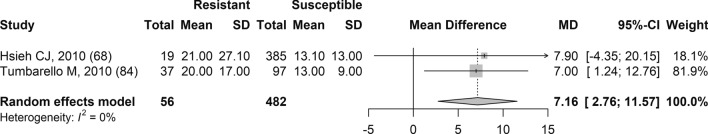
Forest plot of random-effects meta-analysis assessing impact of third-generation cephalosporin-resistant *E. coli* infections on post-infection LOS (length of hospital stay, in days)

Results related to third-generation cephalosporin-resistant *E. coli* infections and post-infection LOS were reported in 7 studies (Additional file 21). Two studies provided the data required to be included in the meta-analysis [68, 84]. Five studies could not be included in the meta-analysis because they reported a median LOS with interquartile range [49, 53, 112], or did not report both a measure of central tendency and variability [42, 65]. Based on the sMD, patients with third-generation cephalosporin-resistant *E. coli* infections had significantly longer post-infection LOS compared to patients with third-generation cephalosporin-susceptible *E. coli* infections (sMD 7.16, 95% CI 2.76–11.57, *p* = 0.031; Fig. 6). The results from the two studies were consistent and there was no heterogeneity (I^2^ 0.0%). Due to serious imprecision, the confidence in the evidence was very low with GRADE assessment (Table 3).

### Healthcare cost

#### Third-generation cephalosporin resistance

Results related to third-generation cephalosporin-resistant *E. coli* infections and healthcare cost were reported in 4 studies (Additional file 22a) [43, 75, 84, 112]. Total cost of the episode was reported in all studies; however, the definitions of total cost varied. In three studies, both direct and indirect or other costs were mentioned [43, 84, 112]. Specific examples of the types of costs considered for inclusion in direct and indirect costs were listed in two of the studies but varied between the two studies [84, 112]. In one study, it was difficult to determine if the cost of the episode included direct and indirect costs or only direct costs [75]. Two studies reported median costs with IQR [75, 112], one study reported median costs with ranges [43], and one study reported mean costs with standard deviations [84]. Euros were the currency used in three studies [75, 84, 112], and the United States dollar was used in one study [43]. The year used for the currency was only reported in one study [84]. Related to total cost: three studies reported a significant increase in total costs when third-generation cephalosporin-resistant *E. coli* infections were compared to third-generation cephalosporin-susceptible *E. coli* infections; and one study reported a non-significant increase in total costs when third-generation cephalosporin-susceptible *E. coli* infections were compared to third-generation cephalosporin-resistant *E. coli* infections. In addition to total cost, one study reported the sub-costs of the total cost of hospitalization and cost of parenteral outpatient antimicrobial therapy [75], and one study reported the average cost per day [112].

#### Multidrug resistance

Two studies reported results related to multidrug-resistant *E. coli* infections and healthcare cost (Additional file 22b) [55, 63]. Total cost was reported in both studies, however, the definitions of total cost varied. In one study, fixed and variable direct costs were mentioned with specific examples of the types of costs considered for inclusion listed [63]. The definition provided in the other study was not explicit [55]. One study reported mean costs with standard deviations and median costs with IQR [63], and the other study reported mean costs without standard deviations [55]. The currency used in one study was Euros [55], and the United States dollar was used in the other study [63]. Both studies reported the year used for the currency. One study reported a significant increase in healthcare cost with multidrug-resistant *E. coli* infections, and one study did not statistically test the difference between the multidrug-resistant and non-multidrug-resistant groups.

## Discussion

### Summary of evidence

We conducted a systematic review and meta-analyses of studies that assessed the impact of antimicrobial-resistant *E. coli* infections compared to susceptible *E. coli* infections on measures of health or healthcare system burden. Resistance to third-generation cephalosporins, resistance to quinolones, and MDR were the types of resistance of interest. The primary outcomes of interest used to assess the health and healthcare system burden were measures of mortality and LOS, respectively. Treatment failure and healthcare costs were used as the secondary outcomes of interest to assess the health and healthcare system burden, respectively. Notably, based on the meta-analyses, for 30-day and all-cause mortality, regardless of the type of AMR, there were significantly higher odds of dying with antimicrobial-resistant *E. coli* infections when compared to susceptible infections. The strength of the association using a sOR was fairly consistent and ranged from 1.49 to 2.27 (95% CI, 1.23–1.82, 1.92–2.70, respectively). These results translated into an estimate of the absolute risk of mortality that ranged from 58 to 130 more people per 1000 (95% CI, 28–93, 98–166, respectively) dying due to resistant *E. coli* infections. There was substantial to considerable heterogeneity when the results from the total LOS studies were combined, and this precluded presentation of a summary mean difference. Small numbers of studies contributed results for bacterium-attributable mortality and post-infection length of stay and therefore, the summary results should be considered with caution and the strength of the evidence was very low. Across the studies, there was considerable variability in definitions and reporting for treatment failure and healthcare costs.

There was an increase in the volume of literature included in the current systematic review (76 studies) compared to the 2014 WHO systematic review (34 studies) [1], which underscores the need for the current systematic review. Compared to the WHO systematic review, we added MDR as a type of resistance. However, there were only six studies included that only addressed MDR without an additional type of resistance, and, therefore, the inclusion of MDR was not the explanation for the increased volume of literature in our review. All of the articles included in the WHO systematic review were based on data from high and upper-middle income countries [1]. This is generally consistent with most of the studies in our systematic review, however, 5.3% of the articles (4/76) were performed in lower-middle income countries. This is an improvement, but there is still a need for more articles from lower-middle and low income countries to allow global generalization. Another factor that limits generalization of the findings is that there were no studies performed in countries in Africa or South America. This is another gap in the literature that deserves future research. Most of the studies included in the current systematic review (75%) were based on data from single centres, which limits their generalizability when the studies are considered individually and not synthesized in a systematic review. The most common type of AMR reported was third-generation cephalosporin resistance (57/76 articles) and this is consistent with the WHO systematic review [1].

For all-cause and 30-day mortality, there was a significant increase in mortality demonstrated with the meta-analyses regardless of the type of AMR considered. The summary measures from previous meta-analyses based on combining smaller numbers of studies are consistent with our results. In the WHO systematic review, the associations between third-generation cephalosporin and fluoroquinolone-resistant *E. coli* infections, and all-cause and 30-day mortality resulted in summary risk ratios (sRR) that ranged from 2.11 to 2.19 (95% CI 1.64–3.71, 1.78–2.68, respectively) [1]. Previous systematic reviews that included all Enterobacteriacea BSI, and were not restricted to *E. coli* BSI, also demonstrated a similar impact of ESBL Enterobacteriacea on all-cause mortality, sRR 1.85 (95% CI 1.39–2.47) [113] and sOR 2.35 (95% CI 1.90–2.91; sOR based on crude OR from the studies) [114].

The mortality measure that would be the most informative regarding the impact of resistance is bacterium-attributable mortality. However, there was a lack of literature reporting that mortality measure with only three studies for third-generation cephalosporin resistance and none for quinolone resistance or MDR. It is understandable why all-cause and 30-day mortality are reported more commonly; it is difficult to fully attribute the cause of the mortality to the bacterial infection when the patients commonly have co-morbidities and the assessment is often being performed based on medical record review. Therefore, we are left with robust information regarding all-cause and 30-day mortality but have to consider that not all of the deaths are likely to be related to the bacterial infection and/or AMR.

An issue highlighted with studies contributing to the LOS analyses is that the distribution of the data needs to be considered. The LOS results were commonly presented as either mean or median LOS for both the susceptible and resistant groups with the appropriate measure of variability. We would need information on the distribution of the data to determine which measure of central tendency was appropriate and that is rarely reported in published studies. Based on the lack of information, we assumed that the studies reported the correct measure of central tendency for the distribution of the LOS data. All of the studies that reported LOS as a median and IQR were excluded from the meta-analyses, which greatly reduced the number of studies contributing data to the meta-analyses. In the three LOS meta-analyses performed, a total of only ten studies were included. Another factor that led to several studies being excluded from the meta-analyses was failure to consistently report both a measure of central tendency and variability. Minimal evidence demonstrating the impact that resistant *E. coli* infections have on LOS was generated by the meta-analyses in this systematic review. There was a significant increase in the post-infection LOS with third-generation cephalosporin-resistant infections, however, this sMD was only based on two studies. The results of the meta-analyses related to total LOS for both third-generation cephalosporin resistance and quinolone resistance had substantial to considerable heterogeneity; therefore, the sMD were not presented. Measures of LOS are important representations of the healthcare system burden [8] and future research in this area is needed. The results of the systematic review highlighted several areas where studies with LOS outcomes could benefit from improved reporting, including: providing details on the distribution of the LOS data to ensure that the appropriate data are being combined in meta-analyses and consistently reporting both the appropriate measure of central tendency and associated measure of variability.

A narrative synthesis was planned and undertaken for the treatment failure and healthcare costs outcomes due to the anticipated substantial variability between study methodologies. Evaluation of the definitions for treatment failure and healthcare costs used in the studies confirmed the substantial variability and lack of standardized definitions. Ideally, we would have standardized definitions that are developed collaboratively by researchers working in the area, and are adopted and utilized by both researchers preparing manuscripts and publishers. Then, meaningful comparisons between studies could be performed, including meta-analyses. In the meantime, we encourage authors to be explicit and fully define how they have used treatment failure and healthcare costs in their studies. It is also important that authors reliably report the currency and year used for healthcare cost outcomes. Consistent with the findings from previous systematic reviews, studies addressing the impact of antimicrobial-resistant *E. coli* infections on healthcare costs are lacking [1, 8].

Assessment of risk of bias is an important aspect of systematic reviews and it provides the end-user with critical information on the strengths and limitations related to bias of the studies included in the systematic review. The domain of bias that had the largest impact on the level of overall risk of bias was bias due to confounding, in which the studies failed to appropriately consider and manage all of the potentially important confounders. Previous review studies have been critical of the lack of appropriate control of confounders and have demonstrated the impact of both failure to adjust for confounders, and inappropriately adjusting for intermediate variables when they should not be included in the multivariable regression model (improperly considering an intervening variable as a confounder) [114, 115]. When crude and adjusted measures of association were compared, the crude measures of association consistently demonstrated a stronger strength of association than the adjusted measures of association [114]. When measures of association were compared with and without adjustment for intervening variables, the measure of association with adjustment for intervening variables demonstrated a weaker strength of association compared to those without adjustment for intervening variables [114, 115]. The intervening variables that are relevant to the relationship between resistant *E. coli* infections and burden of disease outcomes are inappropriate initial antimicrobial therapy, severity of disease, and septic shock and severe sepsis [114, 115]. All three of these intervening variables can be influenced by the presence of resistant *E. coli* infections and lie on the causal pathway between the resistant *E. coli* infections and burden of disease outcomes; therefore, if intervening variables are improperly considered as confounders, the resulting measure of association will likely be biased towards the null value [114, 115]. An important aspect that is not routinely discussed when considering bias due to confounding, is that appropriate control of confounding can be achieved through restricting enrolment to one level of a confounder and matching patients with resistant and susceptible infections based on confounders, not solely through analytical methods to control confounding. Therefore, for each study included in a systematic review, the intricacies of their study design and approach to analysis need to be carefully considered during assessment of bias due to confounding and other domains of bias. During assessment of bias due to confounding for studies in this systematic review, we identified the potentially relevant confounders and assessed whether they were appropriately controlled for using restricted enrolment, matching or analytic methods. We also noted if any intervening variables were controlled for, which could bias the reported measure of association.

GRADE is an accepted method for characterizing the confidence in the cumulative evidence, which is another important feature of systematic reviews [27]. Due to the observational nature of the study designs included in this systematic review, the assessment of quality of evidence for each outcome begins at low. Interestingly, since the completion of this systematic review, GRADE has released guidance that if ROBINS-I is used for risk of bias then the assessment of quality of evidence for each outcome can begin at high [116]. They report that in most cases whether an assessment of a body of evidence from non-randomized studies begins at low or high ultimately they will arrive at the same overall level due to issues with risk of bias [116]. When we re-examined our GRADE assessments following the new guidance, we arrived at the same overall levels in each case. In the context of the research question for this systematic review and many research questions in public health, randomized controlled trials are not performed for valid ethical reasons. When observational study designs are the appropriate and ethical study design, then it would be ideal to compare the body of evidence to a well performed collection of observational studies instead of a well performed collection of randomized controlled trials. However, since evidence from randomized controlled trials is common in other areas of healthcare research, it is not realistic or reasonable to detach observational studies from comparison to randomized controlled trials [116]. Confusion and communication issues may be created when the quality of evidence for outcomes are overwhelmingly or entirely low and very low, such as in this systematic review and the WHO systematic review [1]. Therefore, continued consideration and guidance from the GRADE working group on the value of evidence from observational studies and how to effectively communicate the confidence in evidence from observational studies is needed. In the future, we encourage researchers to design individual observational studies rigorously with particular attention to minimizing selection and confounding bias because this will help to increase the level of the quality of evidence for systematic reviews based on data from observational studies.

### Limitations

There were some limitations both at the study and systematic review level. We did have some deviations from the protocol. They were all minor changes, which were clearly documented in the final systematic review. As previously noted, the domain of bias that was the most problematic for the studies included was bias due to confounding and this limitation was conveyed in the summary of the risk of bias assessments. Unfortunately, a number of studies with LOS data could not be included in the associated meta-analyses because they did not report the LOS data using a mean and standard deviation. As a result, our LOS meta-analyses were based on fewer studies and had high heterogeneity and decreased precision. We only had three meta-analyses that combined more than ten studies (among a total of ten meta-analyses performed) and therefore, allowed assessment of publication bias/small study effects using funnel plots. For the three meta-analyses where funnel plots were constructed, there was some evidence of publication bias/small study effects, but there was a general lack of studies with small sample sizes. The global generalizability of the results from our systematic review is limited because there were no studies from Africa or South America, and none from low income countries.

## Conclusions

Using rigorous systematic review methodology, this manuscript comprehensively synthesized the literature evaluating the impact of select antimicrobial-resistant *E. coli* infections on measures of health and healthcare system burden. All of the meta-analyses for 30-day and all-cause mortality were fairly consistent and, regardless of the type of AMR considered (third-generation cephalosporin, quinolone or MDR), there was a significant increase in the odds of dying when patients had antimicrobial-resistant *E. coli* infections compared to susceptible infections. Of particular public health importance, it was estimated that an extra 58 to 130 people per 1000 (95% CI, 28–93, 98–166, respectively) die because they have resistant *E. coli* infections. There is a need for future *E. coli* infection research addressing the impact of AMR on bacterium-attributable mortality, post-infection LOS, total LOS, and hospital costs. Evaluation of the studies that contributed data for the treatment failure and healthcare costs outcomes revealed a lack of consistency in the definitions used and highlighted opportunities for development of standardized collaborative definitions. In order to facilitate quantitative synthesis of results, and, therefore, more definitive statements on the impact antimicrobial-resistant *E. coli* infections on measures of health and healthcare system burden beyond 30-day and all-cause mortality, we challenge researchers to improve reporting of their studies. By using the appropriate reporting guideline for their study design such as STROBE (Strengthening the Reporting of Observational Studies in Epidemiology) [117], the data necessary to support risk of bias assessment and meta-analysis will be present in their studies. This systematic review provides important evidence of the impact of resistant *E. coli* infections on mortality, and highlights areas that could benefit from future research, standardized collaborative definitions, and improved reporting.

## Supplementary information

**Additional file 1:** A protocol for a systematic review and meta-analysis of the health and healthcare system burden due to human *Escherichia coli* infections resistant to third/fourth/fifth generation cephalosporins or quinolones, or with multidrug resistance (Signed and timestamped)

**Additional file 2:** Ovid MEDLINE^®^ search strategy for systematic review evaluating burden of disease in antimicrobial-resistant *E. coli* infections

**Additional file 3:** Screening questions using for the systematic review

**Additional file 4:** Reference list for studies that met inclusion criteria and were included in the systematic review

**Additional file 5:** Characteristics of the studies and participants included in the systematic review

**Additional file 6:** Characteristics of *E. coli* infections in studies included in the systematic review

**Additional file 7:** Summaries of risk of bias for studies assessing specified burden of disease measure and type of antimicrobial resistance

**Additional file 8:** Results for 30-day mortality and the type of antimicrobial resistance of interest for the systematic review

**Additional file 9:** Funnel plot of the results from the random-effects meta-analysis assessing the impact of third-generation cephalosporin-resistant *E. coli* infections on 30-day mortality

**Additional file 10:** Forest plot for the random-effects meta-analysis assessing the impacts of MDR *E. coli* infections on 30-day mortality

**Additional file 11:** Results for all-cause mortality and the type of antimicrobial resistance of interest for the systematic review

**Additional file 12:** Funnel plot of the results from the random-effects meta-analysis assessing the impact of third-generation cephalosporin-resistant *E. coli* infections on all-cause mortality

**Additional file 13:** Funnel plot of the results from the random-effects meta-analysis assessing the impact of quinolone-resistant *E. coli* infections on all-cause mortality

**Additional file 14:** Forest plot for the random-effects meta-analysis assessing the impacts of MDR *E. coli* infections on all-cause mortality

**Additional file 15:** Results for bacterium-attributable mortality and third-generation cephalosporin-resistant *E. coli* infections for the systematic review

**Additional file 16:** Forest plot for the random-effects meta-analysis assessing the impacts of third-generation cephalosporin-resistant *E. coli* infections on bacterium-attributable mortality

**Additional file 17:** Results for treatment failure and the type of antimicrobial resistance of interest for the systematic review

**Additional file 18:** Results for total length of hospital stay and the type of antimicrobial resistance of interest for the systematic review

**Additional file 19:** Forest plot for the random-effects meta-analysis assessing the impacts of third-generation cephalosporin-resistant *E. coli* infections on total length of hospital stay

**Additional file 20:** Forest plot for the random-effects meta-analysis assessing the impacts of quinolone-resistant *E. coli* infections on total length of hospital stay

**Additional file 21:** Results for post-infection length of hospital stay and third-generation cephalosporin-resistant *E. coli* infections for the systematic review

**Additional file 22:** Results for healthcare costs and the type of antimicrobial resistance of interest for the systematic review

**Additional file 23:** Detailed results of GRADE assessment of primary outcome for the systematic review

## Data Availability

All data generated and analysed during this study are included in this published article or its’ supplementary information files.

## Abbreviations

AMR: Antimicrobial resistance
BSI: Bloodstream infection
CDC: Center for Disease Control and Prevention
CI: Confidence interval
DALYs: Disability-adjusted life years
ECDC: European Centre for Disease Prevention and Control
EMA: European Medicines Agency
ESBL: Extended spectrum β-lactamases
GRADE: Grading of Recommendations Assessment, Development and Evaluation
IQR: Interquartile range
LOS: Length of hospital stay
MD: Mean difference
MeSH: Medical subject headings
MDR: Multidrug resistance
OR: Odds ratio
PHAC: Public Health Agency of Canada
PRISMA: Preferred Reporting Items for Systematic Reviews and Meta-analyses
PROSPERO: International Prospective Register of Systematic Reviews
QALYs: Quality-adjusted life years
R: Resistant
ROBINS-I: Risk of Bias in Non-randomized Studies of Interventions
S: Susceptible
sMD: Summary mean difference
sOR: Summary odds ratio
sRR: Summary risk ratio
WHO: World Health Organization
