# Supplementary material for: Evaluation of the health and healthcare system burden due to antimicrobial-resistant Escherichia coli infections in humans: a systematic review and meta-analysis
Source: Antimicrob Resist Infect Control. 2020 Dec 10;9:200. doi: 10.1186/s13756-020-00863-x (PMC7726913; doi:10.1186/s13756-020-00863-x)
Supplement: Supplementary file 1 — Additional file 1: A protocol for a systematic review and meta-analysis of the health and healthcare system burden due to human Escherichia coli infections resistant to third/fourth/fifth generation cephalosporins or quinolones, or with multidrug resistance (Signed and timestamped) [file 13756_2020_863_MOESM1_ESM.pdf]

Melissa MacKinnon 8:30am
Melissa MacKinnon 2018-10-04

A protocol for a systematic review and meta-analysis of the health and healthcare system burden due to human *Escherichia coli* infections resistant to third/fourth/fifth generation cephalosporins or quinolones, or with multidrug resistance

Registration

The systematic review protocol will be registered with the International Prospective Register of Systematic Reviews (PROSPERO).

Authors

MacKinnon MC[1], Sargeant JM[1,2], Pearl DL[1], Reid-Smith RJ[3], Carson CA[3], Parmley EJ[3], McEwen SA[1].

*Corresponding author: Melissa C MacKinnon mmacki05@uoguelph.ca

Author affiliations

[1] Department of Population Medicine, University of Guelph, Guelph, Ontario, Canada
[2] Centre for Public Health and Zoonoses, University of Guelph, Guelph, Ontario, Canada
[3] Canadian Integrated Program for Antimicrobial Resistance Surveillance (CIPARS), Public Health Agency of Canada, Guelph, Ontario, Canada

Email: Melissa C MacKinnon mmacki05@uoguelph.ca Jan M Sargeant sargeanj@uoguelph.ca – David L Pearl dpearl@uoguelph.ca – Richard J Reid-Smith richard.reid-smith@canada.ca – Carolee A Carson carolee.carson@canada.ca – E Jane Parmley jane.parmley@canada.ca – Scott A McEwen smcewen@uoguelph.ca

Authors' contributions

The review leader is MCM and she will coordinate all aspects of the systematic review. The protocol was developed and written by MCM with input and revisions from all other authors. JMS is the methodological expert and will serve as the third reviewer when necessary. DLP will provide statistic expertise. RJRS, CAC, EJP and SAM are the content experts. The final systematic review manuscript will be written by MCM. All other authors will provide revisions and approve the final version.

Abstract

Background: *Escherichia coli* (*E. coli*) is an important cause of infections in humans, in both community and hospital settings. It is not uncommon for the *E. coli* isolated to be resistant to critically important antimicrobials, which can make treatment challenging. In order to understand the impact of resistant *E. coli* infections on humans, various measures of burden of disease must be evaluated. This protocol describes a systematic review and meta-analysis of the health and healthcare system burden from resistance to third/fourth/fifth generation cephalosporins or quinolones, or multidrug resistance in human *E. coli* infections.

Methods/Design: In order for a study to be included in the systematic review, it must contain the following elements. The population of interest is humans with confirmed *E. coli* infections. Resistance to third/fourth/fifth generation cephalosporins or quinolones, or multidrug resistance are the exposures of interest. There must be a comparator group without the exposure of interest. The outcomes of interest with prioritization for health burden are mortality (primary 1°) and treatment failure (secondary 2°), and for healthcare system burden are length of hospital stay (1°) and costs (2°). The study design must be an analytic observational study. Literature searches will be restricted to 1999 to present. Primary databases to be searched include: MEDLINE®, Embase, Web of Science Current Contents Collection, and Global Health. Grey literature will also be searched. Risk of bias will be assessed using the Risk of Bias in Non-randomized Studies of Interventions (ROBINS-I) from Cochrane. The six domains relevant to exposure studies will be evaluated. If sufficient data are available, primary outcomes will be synthesized by meta-analyses and sources of heterogeneity will be explored using subgroup meta-analyses.

Discussion: The current evidence for the burden of disease from resistance in human *E. coli* infections will be synthesized by the proposed systematic review and meta-analysis.

Keywords: Systematic review, protocol, *E. coli*, human, antimicrobial resistance, burden of disease

<u>Introduction</u>

Rationale

Antimicrobial resistance (AMR) is a critically important global public health issue that could threaten the advances of modern medicine (1, 2). *Escherichia coli (E. coli)* is a commensal organism in humans, but it can also be pathogenic. It is a common cause of community- and hospital-acquired infections (1). Resistance to critically important antimicrobials, including third/fourth/fifth generation cephalosporins and quinolones, is widespread and can complicate the treatment of infections (1, 3). To fully understand the impact of antimicrobial resistance in humans with *E. coli* infection, multiple different aspects of burden of disease must be analyzed (4).

In 2014, the World Health Organization (WHO) released the Antimicrobial Resistance: A Global Report on Surveillance, which included a systematic review and meta-analysis evaluating the health and economic burden of resistance to third generation cephalosporins and fluoroquinolones in human *E. coli* infections (1). Some of the key findings related to third generation cephalosporin resistance when compared to susceptible infections were a significant twofold increase in the three measures of mortality: all-cause; bacterium-attributable; and 30-day mortality (1). Pertaining to fluoroquinolone resistance for all-cause and 30-day mortality there was also a significant twofold increase (1). For the WHO systematic review, the literature searches were performed in March 2013, therefore justifying the current systematic review which will identify literature published since the previous literature search (1). Another important aspect of resistance is multidrug resistance; to our knowledge there is not systematic review evaluating the burden associated with multidrug resistance in human *E. coli* infections.

Objectives

The objective of this protocol is to describe the methods for a systematic review evaluating whether the measures of health or healthcare system burden increase in humans with *E. coli* infections that are resistant to third/fourth/fifth generation cephalosporins, or quinolones or are multidrug resistant when compared to those with susceptible infections.

<u>Methods</u>

Guidelines for preparation

The manuscript for the systematic review will be prepared using Preferred Reporting Items for Systematic review and Meta-analysis [PRISMA] (5, 6). This systematic review protocol was prepared using PRISMA-Protocol [PRISMA-P] (7, 8).

Amendments

During completion of the review, any deviations from this published protocol will be clearly stated and justified in the final systematic review manuscript. The date of the amendment will be included.

Eligibility criteria

Study designs

Any analytic observational study, including appropriate theses or dissertations will be included. Study designs that will be excluded from the review are descriptive observational studies, review articles, commentary, opinion pieces, editorials, newspaper articles, books, and conference proceedings. Due to the nature of the research question, controlled trials are not performed.

Participants

Studies that evaluate humans of any age with an *E. coli* infection (confirmed by culture) will be included. Non-human studies, studies evaluating bacterial infections other than *E. coli*, studies evaluating colonization with *E. coli* instead of infection, and studies evaluating *E. coli* infections that are not confirmed by culture will be excluded.

Exposures

A – Resistance to third/fourth/fifth generation cephalosporins
Studies evaluating resistance to third/fourth/fifth generation cephalosporins (Table 1) or the impact of extended spectrum beta-lactamases will be included.
B – Resistance to quinolones
Studies evaluating resistance to quinolones (Table 1) will be included.
C – Multidrug resistance

Studies evaluating combined resistance to at least three antimicrobial categories or classes will be included (9).
For A and B, studies will also be included if they evaluate combined resistance to A or B and one additional antimicrobial category or class. Studies that evaluate antimicrobial resistance that does not meet the above criteria will be excluded.

Table 1 – List of third/fourth/fifth generation cephalosporins and quinolones (3).

Third/fourth/ fifth generation cephalosporins	cefcapene<br>cefepime<br>cefmenoxime<br>cefoselis<br>cefpiramide<br>cefsulodin<br>ceftazidime-avibactam<br>ceftibuten<br>latamoxef<br>ceftriaxone-sublactam	cefdinir<br>cefetamet<br>cefodizime<br>cefotaxime<br>cefpirome<br>ceftaroline fosamil<br>ceftizoxime<br>ceftolozane<br>tazobactam	cefditoren<br>cefixime<br>cefoperazone<br>cefozopran<br>cefpodoxime<br>ceftazidime<br>ceftobiprole<br>ceftriaxone<br>cefoperazone-sublactam
Quinolones	cinoxacin<br>fleroxacin<br>gatifloxacin<br>levofloxacin<br>nalidixic acid<br>oxolinic acid<br>pipemidic acid<br>rosoxacin<br>sparfloxacin<br>nadifloxacin	ciprofloxacin<br>flumequine<br>gemifloxacin<br>lomefloxacin<br>norfloxacin<br>pazufloxacin<br>piromidic acid<br>rufloxacin<br>temafloxacin	enoxacin<br>garenoxacin<br>grepafloxacin<br>moxifloxacin<br>ofloxacin<br>pefloxacin<br>prulifloxacin<br>sitafloxacin<br>delafloxacin

Comparators

For studies to be included, they must have an appropriate comparator group, which would be humans with *E. coli* infections that are not resistant to third/fourth/fifth generation cephalosporins, or quinolones or are not multidrug resistant. Humans with *E. coli* infections that are pansusceptible are also an acceptable comparator group. Studies that do not have a comparator group or one that meets the criteria listed above will be excluded.

Outcomes

A study evaluating at least one of the following outcomes will be included:

Primary outcome of interest for health burden:

-Mortality (bacterial attributable, all-cause and/or 30-day mortality)

Secondary outcome of interest for health burden:

-Treatment failure as a measure of morbidity

Primary outcome of interest for healthcare system burden:

-Length of hospital stay (LOS and post-infection LOS)

Secondary outcome of interest for healthcare system burden:

-Costs to healthcare system

If a study does not evaluate one of the outcomes above, then it will be excluded.

Publication language

Studies published in English will be included in the review. Although no language restrictions will be placed on the literature search, non-English articles will be identified and excluded during eligibility screening.

Publication dates

The WHO systematic review performed comprehensive literature searches without publication date restrictions and only identified relevant studies that were published from 1999 to 2013 (1). Therefore, literature searches for the current review will be restricted to studies published after December 31st, 1998.

Country

There will be no restrictions on the country where the study was performed.

Information sources

Four literature databases will be searched: MEDLINE®; Embase; Web of Science Current Contents Connect; and Global Health (Table 2). Other resources including grey literature will be searched from the World Health Organization (WHO, including Global Index Medicus), Centers for Disease Control and Prevention (CDC), European Centre for Disease Prevention and Control (ECDC), European Medicines Agency (EMA), Public Health Agency of Canada (PHAC), and Health Canada. The first 250 results sorted based on relevance from Google Scholar will also be screened for eligibility. The lists of references from the studies included in the review will be reviewed to identify any additional potentially relevant articles.

Table 2 – List of databases and platforms for primary literature search

Database	Platform
MEDLINE®	Ovid
Embase	Ovid
Current Contents Connect	Web of Science
Global Health	CAB Direct

Search strategy

The search strategy in Table 3 was developed in consultation with librarians with expertise in systematic reviews. Search terms related to *E. coli* (population), cephalosporins, quinolones and multidrug resistance (exposure), and the outcomes of interest were included in the search strategy. A combination of MeSH terms and keywords were used as the search terms. The search

was restricted to articles published from 1999 to the date the search was performed. There were no search limits imposed related to human studies or English language. The search strategy for MEDLINE in Ovid is included in Table 3. The MEDLINE® search was validated to ensure that 36 articles from the WHO systematic review that were indexed in MEDLINE® were retrieved by the search (1). The search strategy will be modified as required for each database.

Table 3 – Search strategy for Ovid MEDLINE® and In-Process & Other Non-Indexed Citations and Daily.

#	Searches	Results	Annotations
1	Escherichia coli.sh,xs. or Escherichia coli.ab,kf,ti. or e coli.ab,kf,ti.	366819	Population - E. coli terms
2	Cephalosporins.sh,xs. or cephalosporin$.ab,kf,ti. or beta-Lactamases.sh,xs. or b lactamase$.ab,kf,ti. or beta lactamase$.ab,kf,ti. or ESBL.ab,kf,ti. or cefcapene.ab,kf,ti. or cefdinir.ab,kf,ti. or cefditoren.ab,kf,ti. or cefepime.ab,kf,ti. or cefetamet.ab,kf,ti. or cefixime.ab,kf,ti. or cefmenoxime.ab,kf,ti. or cefodizime.ab,kf,ti. or cefoperazone.ab,kf,ti. or cefoselis.ab,kf,ti. or cefotaxime.ab,kf,ti. or cefozopran.ab,kf,ti. or cefpiramide.ab,kf,ti. or cefpirome.ab,kf,ti. or cefpodoxime.ab,kf,ti. or cefsulodin.ab,kf,ti. or ceftaroline.ab,kf,ti. or ceftazidime.ab,kf,ti. or ceftizoxime.ab,kf,ti. or ceftobiprole.ab,kf,ti. or ceftibuten.ab,kf,ti. or ceftolozane.ab,kf,ti. or ceftriaxone.ab,kf,ti. or latamoxef.ab,kf,ti. or tazobactam.ab,kf,ti.	71682	Exposure - Cephalosporin and beta-lactamase terms
3	Quinolones.sh,xs. or fluoroquinolone$.ab,kf,ti. or quinolone$.ab,kf,ti. or cinoxacin$.ab,kf,ti. or ciprofloxacin$.ab,kf,ti. or enoxacin$.ab,kf,ti. or fleroxacin$.ab,kf,ti. or flumequine.ab,kf,ti. or garenoxacin$.ab,kf,ti. or gatifloxacin$.ab,kf,ti. or gemifloxacin$.ab,kf,ti. or grepafloxacin$.ab,kf,ti. or levofloxacin$.ab,kf,ti. or lomefloxacin$.ab,kf,ti. or moxifloxacin$.ab,kf,ti. or nalidixic acid.ab,kf,ti. or norfloxacin$.ab,kf,ti. or ofloxacin$.ab,kf,ti. or oxolinic acid.ab,kf,ti. or pazufloxacin$.ab,kf,ti. or pefloxacin$.ab,kf,ti. or pipemidic acid.ab,kf,ti. or piromidic acid.ab,kf,ti. or	62793	Exposure - Quinolone terms

	prulifloxacin$.ab,kf,ti. or rosoxacin$.ab,kf,ti. or rufloxacin$.ab,kf,ti. or sitafloxacin$.ab,kf,ti. or sparfloxacin$.ab,kf,ti. or temafloxacin$.ab,kf,ti. or delafloxacin$.ab,kf,ti. or nadifloxacin$.ab,kf,ti.		
4	Drug Resistance, Multiple, Bacterial.sh,xs. or (multidrug resistan$ or MDR or multi drug resistan$ or multiple drug resistan$ or extreme$ drug resistan$ or extensive$ drug resistan$ or XDR or pandrug resistan$ or PDR or highly resistan$ or important antimicrobial$ or important antibiotic$).ab,kf,ti.	75834	Exposure - MDR terms
5	(economics, hospital or "Costs and Cost Analysis" or economics, medical).sh,xs. or economics, pharmaceutical.sh. or "fees and charges".sh,xs. or budgets.sh,xs. or (health$care adj cost$).ab,kf,ti. or (cost$ adj variable).ab,kf,ti. or (low adj cost$).ab,kf,ti. or (high adj cost$).ab,kf,ti. or (cost$ adj estimat$).ab,kf,ti. or (unit adj cost$).ab,kf,ti. or (economic$ or pharmacoecomonic$ or pric$).ab,kf,ti.	411134	Outcome - Cost terms
6	hospitalization.sh,xs. or Health Resources.sh. or Utilization Review.sh,xs. or Mortality.sh,xs. or morbidity.sh. or treatment failure.sh,xs. or intensive care units.sh,xs. or "length of stay".ab,kf,ti. or hospital stay.ab,kf,ti. or "resource use".ab,kf,ti. or resource util$.ab,kf,ti. or burden.ab,kf,ti. or mortality.ab,kf,ti. or morbidity.ab,kf,ti. or clinical impact$.ab,kf,ti. or outcome$.ab,kf,ti. or prognos*s.ab,kf,ti. or hospitali$.ab,kf,ti. or fatalit$.ab,kf,ti. or death$.ab,kf,ti. or (ICU or intensive care or critical care).ab,kf,ti. or treatment failure.ab,kf,ti. or failed treatment.ab,kf,ti. or clinical failure.ab,kf,ti. or adverse consequence$.ab,kf,ti. or drug failure$.ab,kf,ti. or epidemiolog$.ab,kf,ti. or factor$.ab,kf,ti. or retreat$.ab,kf,ti.	5641890	Outcome – Non-cost terms
7	1 and (2 or 3 or 4) and (5 or 6)	6318	
8	limit 7 to yr="1999-Current"	**5416**	

Study Records

Data Management

For all articles identified through the searches, the bibliographic citation information including the abstract will be uploaded into EndNote X7 (10) and duplicates will be removed. The bibliographic citation information for all remaining articles will be uploaded to DistillerSR (11) and any remaining duplicates will be removed. DistillerSR (11) will be used to facilitate primary screening, secondary screening, data extraction and assessment of risk of bias.

Selection process

The primary screening will be conducted on the title and abstract of each article. The language of publication will be displayed along with the title and abstract to facilitate answering question 5. The following questions will be used in the primary screening process:

1) Does the title and/ or abstract indicate the study subjects are human?
2) Does the title and/or abstract describe an analytic observational study?
3) Does the title and/or abstract indicate the study participants have *E. coli* infections?
4) Does the title and/or abstract indicate at least some of the study participants have an *E. coli* infection that is resistant to third/fourth/fifth generation cephalosporins, or quinolones, or is multidrug resistant?
5) Is the study published in English?

The primary screening will be performed independently by two researchers. The possible answers are yes, no and unclear. The answers will be compared, and any disagreements will be discussed until consensus is achieved. If consensus cannot be reached, then a third researcher will be used to arbitrate. One or more answers of ‘no’ to the questions above will lead to exclusion of the article. Any combination of ‘yes’ or ‘unclear’ to the questions above will lead to the article proceeding to secondary screening. There will be an initial piloting performed for primary screening on a subset of articles (100 articles) retrieved from the search. Full text articles (PDF format) will be obtained for articles included in secondary screening.

The secondary screening will be performed on the full text articles. The following questions will be used for the secondary screening:

1) Are the study subjects human?
2) Is the study an analytic observational study?
3) Do the study participants have a confirmed infection with *E. coli*?
4) Do at least some of the study participants have an *E. coli* infection that is resistant to third/fourth/fifth generation cephalosporins, or quinolones, or is multidrug resistant?
5) Is there a comparator group that is susceptible to third/fourth/fifth generation cephalosporins, or quinolones, or is not multidrug resistant?
6) Does the study assess at least one outcome of interest?

The secondary screening will be performed independently by two researchers. The possible answers are yes and no. The answers will be compared and any disagreements will be discussed until consensus is achieved. If consensus cannot be reached, then a third researcher will be used

to arbitrate. One or more answers of ‘no’ to the questions above leads to exclusion of the article. Answers of ‘yes’ to all of the questions above leads to the article being included in the systematic review. Information regarding the reason for exclusion will be recorded and all eight questions will be answered for all articles during secondary screening. Five articles will be used for secondary screening piloting.

Data collection process

Data related to the characteristics of the study and study participants, and results for the health and healthcare system outcomes will be extracted. They will be extracted independently by two researchers from all articles included in the study. The data extraction results will be compared, and any disagreements will be resolved using the methods described for primary and secondary screening. The data extraction form will be piloted on 5 articles and revised prior to the extraction of data for the review.

If there is insufficient detail present in the study to facilitate data extraction, the corresponding author will be contacted for studies published within the previous 5 years. This timeframe was instituted to increase the chance of successful contact with the corresponding author. Two e-mails will be sent one month apart and if there is no response within one month after the second e-mail further contact will not be attempted. If the study was published more than 5 years ago or a response is not received from the corresponding author, then the inability to extract the data will be noted in the manuscript.

Data items

During data extraction, Distiller SR (11) will document the researcher performing the extraction, the date of extraction, the unique identifier for the article and the article citation. The data that will be extracted include:

- -Characteristics of the study (year of publication, type of document, author reported study design, year(s) data were collected, country or countries study was performed in, type of site for data collection (eg hospital, community clinic) and number of sites);
- -Characteristics of the study participants (underlying disease processes, definition of cases with resistant (R) infections, number of cases with R infections, definition of cases with susceptible (S) infections in comparator group, number of cases with S infections in comparator group, details of R and S group selection, mean age of R and S groups with measure of variability, distribution of sex in R and S group, source of samples, type of infection, source of participants’ infection, method used to summarize co-morbidities, method used to summarize disease severity, duration of follow-up, and method for antimicrobial susceptibility testing, minimum inhibitory concentration (MIC) interpretive criteria used);
- -Results for health and healthcare system outcomes of interest
    - -The approach to statistical analysis, details of adjustment for confounding and any loss to follow up will be extracted related to all outcome measures.
    - -For all-cause mortality, bacterial-attributable mortality, and 30-day mortality, an adjusted measure of association with measure of variability will be extracted, alternatively a crude measure of association or raw data will be extracted.

-For treatment failure, a description of the measure and associated raw data will be extracted.
-For LOS and post-infection LOS, the mean difference or mean LOS in the R and S groups with measure of variability will be extracted.
-For healthcare system costs, a description of the components included in the cost measure, the cost in the R and S groups with measure of variability, and year and currency for the cost will be extracted.

Outcomes and prioritization

Outcomes that were the most frequently and consistently reported in the previous systematic review were selected as the primary outcomes of interest for the current systematic review (1). Mortality (all-cause, bacterial attributable, and 30-day) and length of hospital stay (LOS and post-infection LOS) were also prioritized as primary outcomes of interest because they are important and meaningful outcomes for *E. coli* infections. Treatment failure was prioritized as a secondary health burden outcome because it is clinically relevant and allows specific assessment of the impact of resistance on treatment of *E. coli* infections. The secondary outcome for healthcare system burden was costs to healthcare system. This measure was selected because quantification of the economic impact of resistance is imperative for holistic characterization of the impact and importance of resistance to modern medicine.

All-cause mortality is when a patient dies due to any cause. There is also no restriction on the length of follow-up. When the cause of death is confirmed to be due to the bacterial (*E.* coli) infection, with no restriction on the length of follow-up, it is bacterial attributable mortality. When the follow-up period is 30-days, it is considered 30-day mortality and the deaths can be due to any cause. There is not a consistent definition of treatment failure in the literature. Treatment failure can include failure to improve or worsening of clinical signs within a given time period (eg 7 days) after initiation of treatment or relapse within a given time period. LOS is the period of time in days from admission to discharge from the hospital. Post-infection LOS is the period of time in days from collection of positive sample to discharge from the hospital. The costs to the healthcare system are calculated using different methods in different studies. In estimates of excess cost, some studies include the excess cost due to length of stay, direct costs (including antimicrobial therapy and diagnostic tests), secondary costs (isolation, staffing, biosecurity), or total cost.

Risk of bias in individual studies

Prior to assessment of risk of bias, the author reported study design will be verified or if a study design is not reported it will be determined. This will be done independently by two researchers and compared to ensure agreement. The assessment of risk-of-bias will be performed independently by two researchers individually for each study for the primary outcomes of interest. A pre-test for the assessment of risk-of-bias will be performed on 5 articles. The Cochrane tool for Risk of Bias in Non-randomized Studies of Interventions (ROBINS-I) modified for use with exposure studies instead of intervention studies will be used to assess risk of bias. The six domains being considered are: bias due to confounding; bias in selection of participants into the study; bias in measurement of exposures; bias due to missing data; bias in

measurement of outcomes; and bias in selection of the reported results. The domain of bias due to departures from intended intervention will not be assessed because it is not relevant in the context of exposure studies. The ROBINS-I signalling questions and criteria for judgement of domain risk of bias will be used to determine the overall risk of bias (low, moderate, serious, critical or no information).

Potential areas where the risk of bias could be high specifically related to this systematic review are as follows:

- -Bias in selection of participants into the study (selection bias), which could occur certain observational study designs due to:
    - -Loss to follow-up (cohort);
    - -Admission risk bias (case-control, secondary base); and
    - -Poor choice of comparison group (cohort or case-control).
- -Important confounders to adjust for:
    - -Co-morbidities;
    - -Severity of underlying illness;
    - -Length of stay prior to infection;
    - -Effectiveness of antimicrobial therapy;
    - -Consideration of exposure as time-dependant;
    - -Age; and
    - -Sex.

Data Synthesis

For all studies, we will determine the country's income status using the World Bank Country Income Classification (12). Data synthesis will be performed separately for studies assessing the impact of resistance to third/fourth/fifth generation cephalosporins, quinolones, and multidrug resistance. Each type of primary outcome will be synthesized independently. If outcome data for mortality are extracted as raw data, then a crude odds ratio will be calculated to allow consideration for inclusion in meta-analysis. If there are at least two studies reporting the same type of mortality outcome using the same measure of association then random effects meta-analysis will be used to summarize data by reporting a summary measure of association. If the same type of LOS is reported by at least two studies, then a random-effects meta-analysis will be used to report a summary mean difference. Cochran's Q test and I^2 will be used to assess heterogeneity. If the Cochran's Q test is significant (p-value ≤ 0.05) and I^2 is $\geq 50\%$, then moderate to substantial heterogeneity will be present and the summary measure will not be presented. If moderate to substantial heterogeneity is present, clinical and methodological heterogeneity will be explored. Clinical heterogeneity is due to the different characteristics of the PECO elements between studies. Potential sources of clinical heterogeneity include the type of *E. coli* infection, mean age in exposure groups, proportion female in exposure groups, country income status, and the type of comparator group (pansusceptible vs susceptible to the antimicrobial of interest). Methodological heterogeneity is due to differences in the study design or execution. Possible sources of methodological heterogeneity include adjusting for confounding bias (co-morbidities, severity of underlying illness, length of stay prior to infection, effectiveness of antimicrobial therapy, consideration of exposure as time-dependant, age, and sex). If there are sufficient data, at least three studies and moderate to substantial heterogeneity,

subgroup analysis based on the above sources of heterogeneity will be performed to explore each possible source of the heterogeneity. A narrative synthesis will be used to summarize data for secondary outcomes. Narrative synthesis will also be used for primary outcomes when there is only one article reporting the outcome, where calculation of a summary measure using meta-analysis could was not appropriate, or where subgroup meta-analysis was not possible.

Meta-bias

For each outcome synthesized, where there are as least 10 studies included in the met-analysis, publication bias will be assessed using funnel plots.

Confidence in cumulative evidence

The confidence in the cumulative evidence for primary outcomes where meta-analysis and/or sub-group meta-analysis will be assessed using GRADE methodology. The criteria used to evaluate the quality of the evidence are risk of bias (based on previously described risk of bias assessment), indirectness, inconsistency, publication bias, and imprecision.

Presentation of results

The characteristics of the studies and case details will be summarized in tables. For each study, the approach to statistical analysis, confounders controlled for, and outcomes assessed will be summarized in a table. The forest plots for meta-analyses and sub-group meta-analyses will be presented. Effect direction plots will be constructed for narratively synthesized data. Risk-of-bias assessment tables will be prepared for each outcome with entries for each study. GRADE summary of findings tables will be prepared using GRADEpro (13) and presented for each primary outcome.

Support

Sources

MC MacKinnon’s PhD program is supported by a Banting and Charles Best Canadian Graduate Scholarships Doctoral Award from the Canadian Institutes of Health Research, a Brock Doctoral Scholarship from the University of Guelph, and an OVC Scholarship from the Ontario Veterinary College (OVC) at the University of Guelph.

Sponsor

There are no sponsors for the review.

Role of sponsor/funder

The organizations providing support for MC MacKinnon’s PhD program, are not involved in any stage of the review.

Acknowledgements

The authors would like to acknowledge University of Guelph Librarians, Ali Versluis and Lucia Costanzo, for their input during the development of the search strategy.

References

1. World Health Organization. Antimicrobial Resistance: Global Report on Surveillance. France 2014. p. 1-256.

2. Gandra S, Barter DM, Laxminarayan R. Economic burden of antibiotic resistance: how much do we really know? Clin Microbiol Infect. 2014;20(10):973-80.

3. World Health Organization. Critically important antimicrobials for human medicine, 5th Revision. 2016.

4. Naylor NR, Atun R, Zhu N, Kulasabanathan K, Silva S, Chatterjee A, et al. Estimating the burden of antimicrobial resistance: a systematic literature review. Antimicrobial resistance and infection control. 2018;7:58.

5. Liberati A, Altman DG, Tetzlaff J, Mulrow C, Gotzsche PC, Ioannidis JP, et al. The PRISMA statement for reporting systematic reviews and meta-analyses of studies that evaluate health care interventions: explanation and elaboration. PLoS medicine. 2009;6(7):e1000100.

6. Moher D, Liberati A, Tetzlaff J, Altman DG. Preferred reporting items for systematic reviews and meta-analyses: the PRISMA statement. PLoS medicine. 2009;6(7):e1000097.

7. Moher D, Shamseer L, Clarke M, Ghersi D, Liberati A, Petticrew M, et al. Preferred reporting items for systematic review and meta-analysis protocols (PRISMA-P) 2015 statement. Systematic reviews. 2015;4:1.

8. Shamseer L, Moher D, Clarke M, Ghersi D, Liberati A, Petticrew M, et al. Preferred reporting items for systematic review and meta-analysis protocols (PRISMA-P) 2015: elaboration and explanation. BMJ (Clinical research ed). 2015;350:g7647.

9. Magiorakos AP, Srinivasan A, Carey RB, Carmeli Y, Falagas ME, Giske CG, et al. Multidrug-resistant, extensively drug-resistant and pandrug-resistant bacteria: an international expert proposal for interim standard definitions for acquired resistance. Clin Microbiol Infect. 2012;18(3):268-81.

10. Clarivate Analytics. EndNote X7. 2016.

11. Evidence Partners. DistillerSR. Ottawa, Canada.

12. The World Bank. World Bank Country and Lending Groups: Country Classification 2018 [Available from: https://datahelpdesk.worldbank.org/knowledgebase/articles/906519-world-bank-country-and-lending-groups.

13. Evidence Prime Inc. GRADEpro GDT: GRADEpro Guideline Development Tool. McMaster University; 2015.
