# Supplementary material for: Evaluation of the health and healthcare system burden due to antimicrobial-resistant Escherichia coli infections in humans: a systematic review and meta-analysis
Source: Antimicrob Resist Infect Control. 2020 Dec 10;9:200. doi: 10.1186/s13756-020-00863-x (PMC7726913; doi:10.1186/s13756-020-00863-x)
Supplement: Supplementary file 2 — Additional file 2: Ovid MEDLINE® search strategy for systematic review evaluating burden of disease in antimicrobial-resistant E. coli infections [file 13756_2020_863_MOESM2_ESM.pdf]

Additional file 2: Ovid MEDLINE® search strategy for systematic review evaluating burden of disease in antimicrobial-resistant *E. coli* infections.

#	Searches	Results	Annotations
1	Escherichia coli.sh,xs. or Escherichia coli.ab,kf,ti. or e coli.ab,kf,ti.	366819	Population - *E. coli* terms
2	Cephalosporins.sh,xs. or cephalosporin$.ab,kf,ti. or beta Lactamases.sh,xs. or b lactamase$.ab,kf,ti. or beta lactamase$.ab,kf,ti. or ESBL.ab,kf,ti. or cefcapene.ab,kf,ti. or cefdinir.ab,kf,ti. or cefditoren.ab,kf,ti. or cefepime.ab,kf,ti. or cefetamet.ab,kf,ti. or cefixime.ab,kf,ti. or cefmenoxime.ab,kf,ti. or cefodizime.ab,kf,ti. or cefoperazone.ab,kf,ti. or cefoselis.ab,kf,ti. or cefotaxime.ab,kf,ti. or cefozopran.ab,kf,ti. or cefpiramide.ab,kf,ti. or cefpirome.ab,kf,ti. or cefpodoxime.ab,kf,ti. or cefsulodin.ab,kf,ti. or ceftaroline.ab,kf,ti. or ceftazidime.ab,kf,ti. or ceftizoxime.ab,kf,ti. or ceftobiprole.ab,kf,ti. or ceftibuten.ab,kf,ti. or ceftolozane.ab,kf,ti. or ceftriaxone.ab,kf,ti. or latamoxef.ab,kf,ti. or tazobactam.ab,kf,ti.	71682	Exposure - Cephalosporin and beta-lactamase terms
3	Quinolones.sh,xs. or fluoroquinolone$.ab,kf,ti. or quinolone$.ab,kf,ti. or cinoxacin$.ab,kf,ti. or ciprofloxacin$.ab,kf,ti. or enoxacin$.ab,kf,ti. or fleroxacin$.ab,kf,ti. or flumequine.ab,kf,ti. or garenoxacin$.ab,kf,ti. or gatifloxacin$.ab,kf,ti. or gemifloxacin$.ab,kf,ti. or grepafloxacin$.ab,kf,ti. or levofloxacin$.ab,kf,ti. or lomefloxacin$.ab,kf,ti. or moxifloxacin$.ab,kf,ti. or nalidixic acid.ab,kf,ti. or norfloxacin$.ab,kf,ti. or ofloxacin$.ab,kf,ti. or oxolinic acid.ab,kf,ti. or pazufloxacin$.ab,kf,ti. or pefloxacin$.ab,kf,ti. or pipemidic acid.ab,kf,ti. or piromidic acid.ab,kf,ti. or prulifloxacin$.ab,kf,ti. or rosoxacin$.ab,kf,ti. or rufloxacin$.ab,kf,ti. or sitafloxacin$.ab,kf,ti. or sparfloxacin$.ab,kf,ti. or temafloxacin$.ab,kf,ti. or delafloxacin$.ab,kf,ti. or nadifloxacin$.ab,kf,ti.	62793	Exposure - Quinolone terms
4	Drug Resistance, Multiple, Bacterial.sh,xs. or (multidrug resistan$ or MDR or multi drug resistan$ or multiple drug resistan$ or extreme$ drug resistan$ or extensive$ drug resistan$ or XDR or pandrug resistan$ or PDR or highly resistan$ or important antimicrobial$ or important antibiotic$).ab,kf,ti.	75834	Exposure - MDR terms
5	(economics, hospital or "Costs and Cost Analysis" or economics, medical).sh,xs. or economics, pharmaceutical.sh. or "fees and charges".sh,xs. or budgets.sh,xs. or (health$care adj cost$).ab,kf,ti. or (cost$ adj variable).ab,kf,ti. or (low adj cost$).ab,kf,ti. or (high adj cost$).ab,kf,ti. or (cost$ adj estimat$).ab,kf,ti. or (unit adj cost$).ab,kf,ti. or (economic$ or pharmacoecomonic$ or pric$).ab,kf,ti.	411134	Outcome - Cost terms
6	hospitalization.sh,xs. or Health Resources.sh. or Utilization Review.sh,xs. or Mortality.sh,xs. or morbidity.sh. or treatment failure.sh,xs. or intensive care units.sh,xs. or "length of stay".ab,kf,ti. or hospital stay.ab,kf,ti. or "resource use".ab,kf,ti. or resource util$.ab,kf,ti. or burden.ab,kf,ti. or mortality.ab,kf,ti. or morbidity.ab,kf,ti. or clinical impact$.ab,kf,ti. or outcome$.ab,kf,ti. or prognos*s.ab,kf,ti. or hospitali$.ab,kf,ti. or fatalit$.ab,kf,ti. or death$.ab,kf,ti. or (ICU or intensive care or critical care).ab,kf,ti. or treatment failure.ab,kf,ti. or failed treatment.ab,kf,ti. or clinical failure.ab,kf,ti. or adverse consequence$.ab,kf,ti. or drug failure$.ab,kf,ti. or epidemiolog$.ab,kf,ti. or factor$.ab,kf,ti. or retreat$.ab,kf,ti.	5641890	Outcome – Non-cost terms
7	1 and (2 or 3 or 4) and (5 or 6)	6318	
8	limit 7 to yr="1999-Current"	**5416**	
