# Supplementary material for: Evaluation of the health and healthcare system burden due to antimicrobial-resistant Escherichia coli infections in humans: a systematic review and meta-analysis
Source: Antimicrob Resist Infect Control. 2020 Dec 10;9:200. doi: 10.1186/s13756-020-00863-x (PMC7726913; doi:10.1186/s13756-020-00863-x)
Supplement: Supplementary file 3 — Additional file 3: Screening questions using for the systematic review [file 13756_2020_863_MOESM3_ESM.pdf]

Additional file 3 – Screening questions for a systematic review evaluating whether the measures of health or healthcare system burden increase in humans with antimicrobial-resistant *E. coli* infections.

Primary Screening

Primary screening was performed on titles and abstracts with possible answers of 'yes,' 'no' or 'unclear.'

1) Does the title and/or abstract indicate the study subjects are human?

2) Does the title and/or abstract describe an analytic observational study?

3) Does the title and/or abstract indicate the study participants have *E. coli* infections?

4) Does the title and/or abstract indicate at least some of the study participants have an *E. coli* infection that is resistant to third/fourth/fifth generation cephalosporins, or quinolones, or is multidrug resistant?

5) Is the study published in English?

Secondary Screening

Secondary screening was performed on full text articles with possible answers of 'yes' or 'no.'

1) Are the study subjects human?

2) Is the study an analytic observational study?

3) Do the study participants have a confirmed infection with *E. coli*?

4) Do at least some of the study participants have an *E. coli* infection that is resistant to third/fourth/fifth generation cephalosporins, or quinolones, or is multidrug resistant?

5) Is there a comparator group that is susceptible to third/fourth/fifth generation cephalosporins, or quinolones, or is not multidrug resistant?

6) Does the study assess at least one outcome of interest?

7) Does the study have outcome data specific to *E. coli* infections?
