# Supplementary material for: Evaluation of the health and healthcare system burden due to antimicrobial-resistant Escherichia coli infections in humans: a systematic review and meta-analysis
Source: Antimicrob Resist Infect Control. 2020 Dec 10;9:200. doi: 10.1186/s13756-020-00863-x (PMC7726913; doi:10.1186/s13756-020-00863-x)
Supplement: Supplementary file 4 — Additional file 4: Reference list for studies that met inclusion criteria and were included in the systematic review [file 13756_2020_863_MOESM4_ESM.pdf]

Additional file 4 - Reference list for studies that met inclusion criteria and were included in the systematic review evaluating whether the measures of health or healthcare system burden increase in humans with antimicrobial-resistant *E. coli* infections.

Note: List is in alphabetical order by first author's last name and to facilitate use with the systematic review manuscript the reference number from the manuscript is in brackets after the bibliographic information.

Abernethy JK, Johnson AP, Guy R *et al.* Thirty day all-cause mortality in patients with *Escherichia coli* bacteraemia in England. *Clin Microbiol Infect* 2015; **21**: 251.e1-8. (49)

Al-Otaibi FE, Bukhari EE. Clinical and laboratory profiles of urinary tract infections caused by extended-spectrum beta-lactamase-producing *Escherichia coli* in a tertiary care center in central Saudi Arabia. *Saudi Med J* 2013; **34**: 171-6. (86)

Alvarez-Uria G, Priyadarshini U, Naik PK *et al*. Mortality associated with community-acquired cephalosporin-resistant *Escherichia coli* in patients admitted to a district hospital in a resource-limited setting. *Clin Pract* 2012; **2**: e76. (36)

Anunnatsiri S, Towiwat P, Chaimanee P. Risk factors and clinical outcomes of extended spectrum beta-lactamase (ESBL)-producing *Escherichia coli* septicemia at Srinagarind University Hospital, Thailand. *Southeast Asian J Trop Med Public Health* 2012; **43**: 1169-77. (41)

Apisarnthanarak A, Kiratisin P, Saifon P *et al*. Predictors of mortality among patients with community-onset infection due to extended-spectrum beta-lactamase-producing *Escherichia coli* in Thailand. *Infect Control Hosp Epidemiol* 2008; **29**: 80-2. (42)

Artero A, Esparcia A, Alberola J *et al*. Prospective cohort study of risk factors for extended-spectrum s-lactamase-producing *Escherichia coli* urinary tract infections in elderly patients admitted to hospital. *Int J Clin Pract* 2017; **71**: e13001. (94)

Bert F, Panhard X, Johnson J *et al*. Genetic background of *Escherichia coli* isolates from patients with spontaneous bacterial peritonitis: relationship with host factors and prognosis. *Clin Microbiol Infect* 2008; **14**: 1034-40. (102)

Bollestad M, Grude N, Solhaug S *et al*. Clinical and bacteriological efficacy of pivmecillinam treatment for uncomplicated urinary tract infections caused by ESBL-producing *Escherichia coli*: a prospective, multicentre, observational cohort study. *J Antimicrob Chemother* 2018; **73**: 2503-9. (106)

Camins BC, Marschall J, DeVader SR *et al*. The clinical impact of fluoroquinolone resistance in patients with *E. coli* bacteremia. *J Hosp Med* 2011; **6**: 344-9. (77)

Can F, Azap OK, Seref C *et al*. Emerging *Escherichia coli* O25b/ST131 clone predicts treatment failure in urinary tract infections. *Clin Infect Dis* 2015; **60**: 523-7. (40)

Cereto F, Herranz X, Moreno E *et al*. Role of host and bacterial virulence factors in *Escherichia coli* spontaneous bacterial peritonitis. *Eur J Gastroenterol Hepatol* 2008; **20**: 924-9. (103)

Cereto F, Molina I, Gonzalez A *et al*. Role of immunosuppression in the development of quinolone-resistant *Escherichia coli* spontaneous bacterial peritonitis and in the mortality of *E. coli* spontaneous bacterial peritonitis. *Aliment Pharmacol Ther* 2003; **17**: 695-701. (101)

Chakraborty A, Adhikari P, Shenoy S *et al*. Extra-intestinal beta-lactamase producing *Escherichia coli* infection-an emerging infection in a South Indian tertiary care hospital. *J Clin Diagn Res* 2012; **6**: 1210-4. (37)

Chauhan S. Evaluation of risk factors and clinical outcome related to ESBL-producing *E. coli* infection among hospitalized patients. *IOSR-JDMS* 2015; **14**: 100-4. (39)

Cheong HJ, Yoo CW, Sohn JW *et al*. Bacteremia due to quinolone-resistant *Escherichia coli* in a teaching hospital in South Korea. *Clin Infect Dis* 2001; **33**: 48-53. (100)

Cheong HS, Kang CI, Kwon KT *et al*. Clinical significance of healthcare-associated infections in community-onset *Escherichia coli* bacteraemia. *J Antimicrob Chemother* 2007; **60**: 1355-60. (59)

Cornejo-Juarez P, Perez-Jimenez C, Silva-Sanchez J *et al*. Molecular analysis and risk factors for *Escherichia coli* producing extended-spectrum beta-lactamase bloodstream infection in hematological malignancies. *PLoS One* 2012; **7**: e35780. (43)

Courpon-Claudinon A, Lefort A, Panhard X *et al*. Bacteraemia caused by third-generation cephalosporin-resistant *Escherichia coli* in France: Prevalence, molecular epidemiology and clinical features. *Clinical Microbiol Infect* 2011; **17**: 557-65. (84)

de Kraker ME, Wolkewitz M, Davey PG *et al*. Burden of antimicrobial resistance in European hospitals: excess mortality and length of hospital stay associated with bloodstream infections due to *Escherichia coli* resistant to third-generation cephalosporins. *J Antimicrob Chemother* 2011; **66**: 398-407. (48)

Denis B, Lafaurie M, Donay JL *et al*. Prevalence, risk factors, and impact on clinical outcome of extended-spectrum beta-lactamase-producing *Escherichia coli* bacteraemia: a five-year study. *Int J Infect Dis* 2015; **39**: 1-6. (73)

Eom JS, Hwang BY, Sohn JW *et al*. Clinical and molecular epidemiology of quinolone-resistant *Escherichia coli* isolated from urinary tract infection. *Microb Drug Resist* 2002; **8**: 227-34. (76)

Esteve-Palau E, Solande G, Sanchez F *et al*. Clinical and economic impact of urinary tract infections caused by ESBL-producing *Escherichia coli* requiring hospitalization: a matched cohort study. *J Infect* 2015; **71**: 667-74. (74)

Fan NC, Chen HH, Chen CL *et al*. Rise of community-onset urinary tract infection caused by extended-spectrum beta-lactamase-producing *Escherichia coli* in children. *J Microbiol Immunol Infect* 2014; **47**: 399-405. (107)

Feng XR, Yang X, Yi CY *et al*. *Escherichia coli* peritonitis in peritoneal dialysis: the prevalence, antibiotic resistance and clinical outcomes in a south China dialysis center. *Perit Dial Int* 2014; **34**: 308-16. (46)

Freeman JT, McBride SJ, Nisbet MS *et al*. Bloodstream infection with extended-spectrum beta-lactamase-producing Enterobacteriaceae at a tertiary care hospital in New Zealand: risk factors and outcomes. *Int J Infect Dis* 2012; **16**: e371-4. (69)

Gagliotti C, Buttazzi R, Sforza S *et al*. Resistance to fluoroquinolones and treatment failure/short-term relapse of community-acquired urinary tract infections caused by *Escherichia coli*. *J Infect* 2008; **57**: 179-84. (110)

Garau J, Xercavins M, Rodriguez-Carballeira M *et al*. Emergence and dissemination of quinolone-resistant *Escherichia coli* in the community. *Antimicrob Agents Chemother* 1999; **43**: 2736-41. (99)

Gudiol C, Calatayud L, Garcia-Vidal C *et al*. Bacteraemia due to extended-spectrum beta-lactamase-producing *Escherichia coli* (ESBL-EC) in cancer patients: clinical features, risk factors, molecular epidemiology and outcome. *J Antimicrob Chemother* 2010; **65**: 333-41. (66)

Ha YE, Kang CI, Cha MK *et al*. Epidemiology and clinical outcomes of bloodstream infections caused by extended-spectrum beta-lactamase-producing *Escherichia coli* in patients with cancer. *Int J Antimicrob Agents* 2013; **42**: 403-9. (71)

Haruki Y, Hagiya H, Haruki M *et al*. Clinical characteristics and outcome of critically ill patients with bacteremia caused by extended-spectrum beta-lactamase-producing and non-producing *Escherichia coli*. *J Infect Chemother* 2018; **24**: 944-7. (75)

Henshke-Bar-Meir R, Yinnon AM, Rudensky B *et al*. Assessment of the clinical significance of production of extended-spectrum beta-lactamases (ESBL) by Enterobacteriaceae. *Infection* 2006; **34**: 66-74. (79)

Ho PL, Chan WM, Tsang KWT *et al*. Bacteremia caused by *Escherichia coli* producing extended-spectrum beta-lactamase: a case-control study of risk factors and outcomes. *Scand J Infect Dis* 2002; **34**: 567-73. (63)

Hsieh CJ, Shen YH, Hwang KP. Clinical implications, risk factors and mortality following community-onset bacteremia caused by extended-spectrum beta-lactamase (ESBL) and non-ESBL producing *Escherichia coli*. *J Microbiol Immunol Infect* 2010; **43**: 240-8. (67)

Huang YY, Alleyne A, Leung V *et al*. Urosepsis due to extended-spectrum beta-lactamase-producing *Escherichia coli*: a retrospective, single-centre review of risk factors and clinical outcomes. *Can J Hosp Pharm* 2018; **71**: 119-27. (96)

Huotari K, Tarkka E, Valtonen V *et al*. Incidence and risk factors for nosocomial infections caused by fluoroquinolone-resistant *Escherichia coli*. *Eur J Clin Microbiol Infect Dis* 2003; **22**: 492-5. (53)

Jeon JH, Kim K, Han WD *et al*. Empirical use of ciprofloxacin for acute uncomplicated pyelonephritis caused by *Escherichia coli* in communities where the prevalence of fluoroquinolone resistance is high. *Antimicrob Agents Chemother* 2012; **56**: 3043-6. (109)

Kang CI, Chung DR, Ko KS *et al*. Risk factors for mortality and impact of broad-spectrum cephalosporin resistance on outcome in bacteraemic intra-abdominal infections caused by Gram-negative bacilli. *Scand J Infect Dis* 2011; **43**: 202-8. (51)

Kang CI, Song JH, Chung DR *et al*. Risk factors and treatment outcomes of community-onset bacteraemia caused by extended-spectrum beta-lactamase-producing *Escherichia coli*. *Int J Antimicrob Agents* 2010; **36**: 284-7. (50)

Kang CI, Wi YM, Lee MY *et al*. Epidemiology and risk factors of community onset infections caused by extended-spectrum beta-lactamase-producing *Escherichia coli* strains. *J Clin Microbiol* 2012; **50**: 312-7. (70)

Kaya O, Akcam FZ, Gonen I *et al*. Risk factors for bacteremia due to extended-spectrum beta-lactamase-producing *Escherichia coli* in a Turkish hospital. *J Infect Dev Ctries* 2013; **7**: 507-12. (45)

Khan FY, Elshafie SS, Almaslamani M *et al*. Epidemiology of bacteraemia in Hamad general hospital, Qatar: A one year hospital-based study. *Travel Med Infect Dis* 2010; **8**: 377-87. (81)

Kim J, Kang CI, Joo EJ *et al*. Risk factor of community-onset spontaneous bacterial peritonitis caused by fluoroquinolone-resistant *Escherichia coli* in patients with cirrhosis. *Liver Int* 2014; **34**: 695-9. (78)

Kim SH, Kwon JC, Choi SM *et al*. *Escherichia coli* and *Klebsiella pneumoniae* bacteremia in patients with neutropenic fever: factors associated with extended-spectrum beta-lactamase production and its impact on outcome. *Ann Hematol* 2013; **92**: 533-41. (72)

Komatsu Y, Kasahara K, Inoue T *et al*. Molecular epidemiology and clinical features of extended-spectrum beta-lactamase- or carbapenemase-producing *Escherichia coli* bacteremia in Japan. *PLoS One* 2018; **13**: e0202276. (97)

Lambert ML, Suetens C, Savey A *et al*. Clinical outcomes of health-care-associated infections and antimicrobial resistance in patients admitted to European intensive-care units: a cohort study. *Lancet Infect Dis* 2011; **11**: 30-8. (52)

Laupland KB, Gregson DB, Church DL *et al*. Incidence, risk factors and outcomes of *Escherichia coli* bloodstream infections in a large Canadian region. *Clin Microbiol Infect* 2008; **14**: 1041-7. (104)

Lee H, Han SB, Kim JH *et al*. Risk factors of urinary tract infection caused by extended spectrum beta-lactamase-producing *Escherichia coli* in emergency department. *Am J Emerg Med* 2018; **36**: 1608-12. (98)

Lee S, Song DY, Cho SH *et al*. Impact of extended-spectrum beta-lactamase on acute pyelonephritis treated with empirical ceftriaxone. *Microb Drug Resist* 2014; **20**: 39-44. (88)

Leistner R, Bloch A, Sakellariou C *et al*. Costs and length of stay associated with extended-spectrum beta-lactamase production in cases of *Escherichia coli* bloodstream infection. *J Glob Antimicrob Resist* 2014; **2**: 107-9. (111)

Leistner R, Gurntke S, Sakellariou C *et al*. Bloodstream infection due to extended-spectrum beta-lactamase (ESBL)-positive *K. pneumoniae* and *E. coli*: an analysis of the disease burden in a large cohort. *Infection* 2014; **42**: 991-7. (90)

Leistner R, Sakellariou C, Gurntke S *et al*. Mortality and molecular epidemiology associated with extended-spectrum beta-lactamase production in *Escherichia coli* from bloodstream infection. *Infect Drug Resist* 2014; **7**: 57-62. (89)

Lim C, Takahashi E, Hongsuwan M *et al*. Epidemiology and burden of multidrug-resistant bacterial infection in a developing country. *elife* 2016; **5**: e18082. (44)

Ma J, Li N, Liu YJ *et al*. Antimicrobial resistance patterns, clinical features, and risk factors for septic shock and death of nosocomial *E. coli* bacteremia in adult patients with hematological disease A monocenter retrospective study in China. *Medicine (Baltimore)* 2017; **96**: E6959. (47)

Martelius T, Jalava J, Karki T *et al*. Nosocomial bloodstream infections caused by *Escherichia coli* and *Klebsiella pneumoniae* resistant to third-generation cephalosporins, Finland, 1999-2013: trends, patient characteristics and mortality. *Infect Dis (Lond)* 2016; **48**: 229-34. (55)

Maslikowska JA, Walker SAN, Elligsen M *et al*. Impact of infection with extended-spectrum beta-lactamase-producing *Escherichia coli* or *Klebsiella* species on outcome and hospitalization costs. *J Hosp Infect* 2016; **92**: 33-41. (92)

Melzer M, Petersen I. Mortality following bacteraemic infection caused by extended spectrum beta-lactamase (ESBL) producing *E. coli* compared to non-ESBL producing *E. coli*. *J Infect* 2007; **55**: 254-9. (64)

Namikawa H, Yamada K, Fujimoto H *et al*. Clinical characteristics of bacteremia caused by extended-spectrum beta-lactamase-producing *Escherichia coli* at a tertiary hospital. *Intern Med* 2017; **56**: 1807-15. (95)

Nicolas-Chanoine MH, Jarlier V, Robert J *et al*. Patient's origin and lifestyle associated with CTX-M-producing *Escherichia coli*: a case-control-control study. *PLoS One* 2012; **7**: e30498. (85)

Nussbaum A, Mariano N, Colon-Urban R *et al*. Microbiologic and clinical comparison of catients harboring *Escherichia coli* blood isolates with and without extended-spectrum β-lactamases. *Adv Infect Dis* 2013; **3**: 50-4. (87)

Ortega M, Marco F, Soriano A *et al*. Analysis of 4758 *Escherichia coli* bacteraemia episodes: predictive factors for isolation of an antibiotic-resistant strain and their impact on the outcome. *J Antimicrob Chemother* 2009; **63**: 568-74. (57)

Park SH, Choi SM, Lee DG *et al*. Impact of extended-spectrum beta-lactamase production on treatment outcomes of acute pyelonephritis caused by *Escherichia coli* in patients without health care-associated risk factors. *Antimicrob Agents Chemother* 2015; **59**: 1962-8. (93)

Park SH, Choi SM, Lee DG *et al*. Emergence of extended-spectrum beta-lactamase-producing *Escherichia coli* as a cause of community-onset bacteremia in South Korea: risk factors and clinical outcomes. *Microb Drug Resist* 2011; **17**: 537-44. (68)

Parveen A, Sultan F, Raza A *et al*. Bacteraemia caused by *Escherichia coli* in cancer patients at a specialist center in Pakistan. *J Pak Med Assoc* 2015; **65**: 1271-6. (38)

Pena C, Gudiol C, Calatayud L *et al*. Infections due to *Escherichia coli* producing extended-spectrum beta-lactamase among hospitalised patients: factors influencing mortality. *J Hosp Infect* 2008; **68**: 116-22. (65)

Peralta G, Sanchez MB, Garrido JC *et al*. Impact of antibiotic resistance and of adequate empirical antibiotic treatment in the prognosis of patients with *Escherichia coli* bacteraemia. *J Antimicrob Chemother* 2007; **60**: 855-63. (56)

Riu M, Chiarello P, Terradas R *et al*. Cost attributable to nosocomial bacteremia. Analysis according to microorganism and antimicrobial sensitivity in a university hospital in Barcelona. *PLoS One* 2016; **11**: e0153076. (54)

Rodriguez-Bano J, Picon E, Gijon P *et al*. Community-onset bacteremia due to extended-spectrum beta-lactamase-producing *Escherichia coli*: risk factors and prognosis. *Clin Infect Dis* 2010; **50**: 40-8. (82)

Shin J, Kim J, Wie SH *et al*. Fluoroquinolone resistance in uncomplicated acute pyelonephritis: epidemiology and clinical impact. *Microb Drug Resist* 2012; **18**: 169-75. (105)

Soraas A, Sundsfjord A, Jorgensen SB *et al*. High rate of per oral mecillinam treatment failure in community-acquired urinary tract infections caused by ESBL-producing *Escherichia coli*. *PLoS One* 2014; **9**: e85889. (108)

Thaden JT, Li YH, Ruffin F *et al*. Increased costs associated with bloodstream infections caused by multidrug-resistant gram-negative bacteria are due primarily to patients with hospital-acquired infections. *Antimicrob Agents Chemother* 2017; **61**: e01709-16. (62)

Trecarichi EM, Tumbarello M, Spanu T *et al*. Incidence and clinical impact of extended-spectrum-beta-lactamase (ESBL) production and fluoroquinolone resistance in bloodstream infections caused by *Escherichia coli* in patients with hematological malignancies. *J Infect* 2009; **58**: 299-307. (58)

Tumbarello M, Spanu T, Di Bidino R *et al*. Costs of bloodstream infections caused by *Escherichia coli* and influence of extended-spectrum-beta-lactamase production and inadequate initial antibiotic therapy. *Antimicrob Agents Chemother* 2010; **54**: 4085-91. (83)

Uzodi AS, Lohse CM, Banerjee R. Risk factors for and outcomes of multidrug-resistant *Escherichia coli* infections in children. *Infect Dis Ther* 2017; **6**: 245-57. (61)

Van Aken S, Lund N, Ahl J *et al*. Risk factors, outcome and impact of empirical antimicrobial treatment in extended-spectrum beta-lactamase-producing *Escherichia coli* bacteraemia. *Scand J Infect Dis* 2014; **46**: 753-62. (91)

Yip T, Tse KC, Lam MF *et al*. Risk factors and outcomes of extended-spectrum beta-lactamase-producing *E. coli* peritonitis in CAPD patients. *Perit Dial Int* 2006; **26**: 191-7. (80)

Yoon EJ, Choi MH, Park YS *et al*. Impact of host-pathogen-treatment tripartite components on early mortality of patients with *Escherichia coli* bloodstream infection: prospective observational study. *EBioMedicine* 2018; **35**: 76-86. (60)
