# Supplementary material for: Evaluation of the health and healthcare system burden due to antimicrobial-resistant Escherichia coli infections in humans: a systematic review and meta-analysis
Source: Antimicrob Resist Infect Control. 2020 Dec 10;9:200. doi: 10.1186/s13756-020-00863-x (PMC7726913; doi:10.1186/s13756-020-00863-x)
Supplement: Supplementary file 5 — Additional file 5: Characteristics of the studies and participants included in the systematic review [file 13756_2020_863_MOESM5_ESM.pdf]

Additional file 5 - Characteristics of the studies and participants included in systematic review evaluating whether the measures of health or healthcare system burden increase in humans with antimicrobial-resistant *E. coli* infections

Study Characteristics										Demographics (Age and Sex)				BoD / Overall
Citation (Reference # in manuscript)	Type of Resistance	Author reported study design	Study design in the context of systematic review outcomes	Prospective or retrospective	Country	World Bank income status	Data Collection Years	Number of Sites	Details of Site(s)	General age(s) included in	Overall % female	% female in R group	% female in S	BoD Outcome
Abernethy, J. K., *et al.* 2015. (50)	Third/fourth/fifth generation cephalosporins or ESBLs	Surveillance	Cohort	Retrospective	England	High	07/2011 - 06/2012	≥3	National mandatory surveillance in England	No age restriction in sampling	53.2	nr	nr	1) 30-day mortality; 2) All-cause mortality
Abernethy, J. K., *et al.* 2015. (50)	Quinolones	Surveillance	Cohort	Retrospective	England	High	07/2011 - 06/2012	≥3	National mandatory surveillance in England	No age restriction in sampling	53.2	nr	nr	1) 30-day mortality; 2) All-cause mortality
Al-Otaibi, F. E., *et al.* 2013. (87)	Third/fourth/fifth generation cephalosporins or ESBLs	Clinical and laboratory study	Cohort	Retrospective	Saudi Arabia	High	06/2009 - 06/2011	1	Tertiary care hospital, King Khalid University Hospital, Riyadh, KSA	No age restriction in sampling	71.7	56	nr	1) All-cause mortality
Alvarez-Uria, G., *et al.* 2012. (37)	Third/fourth/fifth generation cephalosporins or ESBLs	Observational	Cohort	Unable to determine	India	Lower-middle	03/2011 - 03/2012	1	Bathalapalli Rural Development Trust Hospital, non-profit private district hospital in Andhra Pradesh, India (resource limited district hospital in India)	No age restriction in sampling	37.1	nr	nr	1) All-cause mortality
Anunnatsiri, S., *et al.* 2012. (42)	Third/fourth/fifth generation cephalosporins or ESBLs	Not reported	Cohort	Retrospective	Thailand	Upper-middle	01/2005 - 12/2006	1	Tertiary care hospital, Srinagarind Hospital, Thailand	≥15	nr	46.9	57.5	1) All-cause mortality, 2) Treatment failure, 3) Post-infection length of hospital stay
Apisarnthanarak, A., *et al.* 2008. (43)	Third/fourth/fifth generation cephalosporins or ESBLs	Case-control	Cohort	Retrospective	Thailand	Upper-middle	07/2003 - 06/2004	1	Tertiary care hospital, Thammasart University Hospital, in Praturmthani, Thailand	≥ 16	nr	71	74	1) All-cause mortality, 2) Length of hospital stay, 3) Healthcare system costs
Artero, A., *et al.* 2017. (95)	Third/fourth/fifth generation cephalosporins or ESBLs	Cohort	Cohort	Prospective	Spain	High	01/2013 - 12/2015	1	Tertiary-care hospital, University Hospital Dr. Peset, Valencia, Spain	≥65	64	62.4	64.5	1) All-cause mortality, 2) Length of hospital stay

Bert, F., *et al.* 2008. (103)	Quinolones	Not reported	Cohort	Retrospective	France	High	01/1997 - 12/2005	1	Hospital AP-HP Beaujon, Clichy, France	No age restriction in sampling	21.1	nr	nr	1) All-cause mortality
Bollestad, M., *et al.* 2018. (107)	Third/fourth/fifth generation cephalosporins or ESBLs	Cohort	Cohort	Prospective	Norway	High	04/2013 - 08/2016	≥3	8 microbiology laboratories (patients included from 76 different geographically diverse municipalities in Norway)	≥16	100	100	100	1) Treatment failure
Camins, B. C., *et al.* 2011. (78)	Quinolones	Case-control	Cohort	Retrospective	USA	High	01/2000 - 12/2005	1	Barnes-Jewish Hospital, St. Louis, Missouri	No age restriction in sampling	nr	66	53	1) 30-day mortality, 2) All-cause mortality, 3) Treatment failure, 4) Length of hospital stay
Can, F., *et al.* 2015. (41)	Third/fourth/fifth generation cephalosporins or ESBLs	Cohort	Cohort	Prospective	Turkey	Upper-middle	01/2011 - 12/2011	1	Outpatient clinic, Baskent University Hospital, Ankara, Turkey	No age restriction in sampling	nr	nr	nr	1) Treatment failure
Can, F., *et al.* 2015. (41)	Quinolones	Cohort	Cohort	Prospective	Turkey	Upper-middle	01/2011 - 12/2011	1	Outpatient clinic, Baskent University Hospital, Ankara, Turkey	No age restriction in sampling	nr	nr	nr	1) Treatment failure
Can, F., *et al.* 2015. (41)	Multidrug resistance	Cohort	Cohort	Prospective	Turkey	Upper-middle	01/2011 - 12/2011	1	Outpatient clinic, Baskent University Hospital, Ankara, Turkey	No age restriction in sampling	nr	nr	nr	1) Treatment failure
Cereto, F., *et al.* 2008. (104)	Quinolones	Not reported	Cohort	Prospective	Spain	High	01/2004 - 12/2005	≥3	Three tertiary hospitals from the Barcelona area	No age restriction in sampling	nr	34	28	1) All-cause mortality, 2) Treatment failure
Cereto, F., *et al.* 2008. (104)	Quinolones	Not reported	Cohort	Retrospective	Spain	High	01/1996 - 06/2002	1	Hospital Universitari Vall d'Hebron, Barcelona, Spain	No age restriction in sampling	nr	50	29	1) All-cause mortality
Chakraborty, A., *et al.* 2012. (38)	Third/fourth/fifth generation cephalosporins or ESBLs	Not reported	Cohort	Retrospective	India	Lower-middle	05/2010 - 07/2011	1	Tertiary care hospital at Mangalore	No age restriction in sampling	nr	37.9	51.5	1) All-cause mortality, 2) Treatment failure

Chauhan, S., 2015. (40)	Third/fourth/fifth generation cephalosporins or ESBLs	Case-control	Cohort	Unable to determine	India	Lower-middle	01/2012-08/2012	1	One hospital in Northern India, Shri Mahant Indresh Hospital and Shri Guru Ram rai Institute of Health and medical Sciences, Dehradun city - teaching hospital in north India.	No age restriction in sampling		60	59	1) Treatment failure, 2) Length of stay
Cheong, H. J., *et al.* 2001. (101)	Quinolones	Case-control	Cohort	Retrospective	Republic of Korea	High	09/1993 - 08/1998	1	Korea University Guro Hospital	No age restriction in sampling	nr	30	58.8	1) All-cause mortality
Cheong, H. S., *et al.* 2007. (60)	Third/fourth/fifth generation cephalosporins or ESBLs	Cohort	Cohort	Retrospective	Republic of Korea	High	02/2002 - 12/2005	1	Samsung Medical Centre, Seoul, Korea	≥ 15	nr	nr	nr	1) 30-day mortality, 2) All-cause mortality
Cheong, H. S., *et al.* 2007. (60)	Quinolones	Cohort	Cohort	Retrospective	Republic of Korea	High	02/2002 - 12/2005	1	Samsung Medical Centre, Seoul, Korea	≥ 15	nr	nr	nr	1) 30-day mortality, 2) All-cause mortality
Cheong, H. S., *et al.* 2007. (60)	Multidrug resistance	Cohort	Cohort	Retrospective	Republic of Korea	High	02/2002 - 12/2005	1	Samsung Medical Centre, Seoul, Korea	≥ 15	nr	nr	nr	1) 30-day mortality, 2) All-cause mortality
Cornejo-Juarez, P., *et al.* 2012. (44)	Third/fourth/fifth generation cephalosporins or ESBLs	Case-control	Cohort	Retrospective	Mexico	Upper-middle	01/2004 - 12/2009	1	Instituto Nacional de Cancerologia, tertiary oncology teaching hospital in Mexico	No age restriction in sampling	49	55	42	1) All-cause mortality, 2) Bacterium-attributable mortality
Courpon-Claudinon, A. *et al.* 2011. (85)	Third/fourth/fifth generation cephalosporins or ESBLs	Not reported	Cohort	Prospective	France	High	01/2005 - 12/2005	≥3	15 hospitals across France, all except one were university hospitals	≥ 18	nr	41	58.1	1) All-cause mortality
de Kraker, M. E., *et al.* 2011. (49)	Third/fourth/fifth generation cephalosporins or ESBLs	Cohort	Cohort	Prospective	13 European Countries	High; Upper-middle	07/2007 - 06/2008	≥3	13 tertiary care centres in 13 European countries: Austria, Belgium, Croatia, England, Germany, Greece, Ireland, Italy, Latvia, Malta, Romania (upper-middle), Scotland, Slovenia	≥ 18	nr	52	56	1) 30-day mortality, 2) All-cause mortality, 3) Post-infection length of stay
Denis, B., *et al.* 2015. (74)	Third/fourth/fifth generation cephalosporins or ESBLs	Case-control	Cohort	Retrospective	France	High	01/2005 - 12/2008	1	Saint Louis Hospital, tertiary hospital	No age restriction in sampling	nr	34	32	1) 30-day mortality, 2) All-cause mortality, 3) Length of stay

Eom, J. S., *et al.* 2002. (77)	Quinolones	Case-control	Cohort	Retrospective	Republic of Korea	High	01/2000 - 12/2000	1	Korea University Guro Hospital	No age restriction in sampling	nr	73.3	70	1) 30-day mortality, 2) All-cause mortality, 3) Treatment failure
Esteve-Palau, E., *et al.* 2015. (75)	Third/fourth/fifth generation cephalosporins or ESBLs	Cohort	Cohort	Retrospective	Spain	High	08/2010 - 07/2013	1	Hospital del Mar, Barcelona, Spain	≥ 18	43	43	43	1) 30-day mortality, 2) All-cause mortality, 3) Treatment failure, 4) Length of stay, 5) Healthcare system costs
Fan, N. C., *et al.* 2014. (108)	Third/fourth/fifth generation cephalosporins or ESBLs	Case-control	Cohort	Retrospective	Taiwan	High	01/2002 - 12/2006	1	Chang Gung Children's Hospital	< 15 yr old	nr	nr	nr	1) Treatment failure, 2) Length of stay
Feng, X. R., *et al.* 2014. (47)	Third/fourth/fifth generation cephalosporins or ESBLs	Not reported	Cohort	Prospective	China	Upper-middle	01/2006 - 12/2011	1	First Affiliated Hospital of Sun Yat-sen University, Guangzhou, China	No age restriction in sampling	39.7	nr	nr	1) Bacterium-attributable mortality, 2) Treatment failure
Freeman, J. T., *et al.* 2012. (70)	Third/fourth/fifth generation cephalosporins or ESBLs	Case-control	Cohort	Retrospective	New Zealand	High	05/2003 - 03/2007	1	Middlemore Hospital (University-affiliated tertiary hospital) Auckland	No age restriction in sampling	nr	nr	nr	1) 30-day mortality, 2) All-cause mortality
Gagliotti, C., *et al.* 2008. (111)	Quinolones	Cohort	Cohort	Prospective	Italy	High	01/2006 - 12/2006	≥ 3	Database of Antimicrobial Resistance Surveillance System of Emilia-Romagna (includes laboratories belonging to 13 of 17 Local health trusts of the region)	≥ 18	100	100	100	1) Treatment failure
Garau, J., *et al.* 1999. (100)	Quinolones	Not reported	Cohort	Retrospective	Spain	High	01/1992 - 12/1997	1	The Hospital Mutua de Terrassa, Terrassa, Spain	No age restriction in sampling	nr	37.1	47.4	1) All-cause mortality
Gudiol, C., *et al.* 2010. (67)	Third/fourth/fifth generation cephalosporins or ESBLs	Observational	Cohort	Prospective	Spain	High	01/2006 - 10/2008	1	University cancer centre for adults	≥ 18	41	71	37	1) 30-day mortality, 2) All-cause mortality
Ha, Y. E., *et al.* 2013. (72)	Third/fourth/fifth generation cephalosporins or ESBLs	Cohort	Cohort	Retrospective	Republic of Korea	High	01/2010 - 05/2012	1	Samsung Medical Center, Seoul, Korea	≥ 16	45.1	42.1	46.3	1) 30-day mortality, 2) All-cause mortality

Haruki, Y., *et al.* 2018. (76)	Third/fourth/fifth generation cephalosporins or ESBLs	Cohort	Cohort	Retrospective	Japan	High	01/2006 - 12/2006	1	Tsuyama Chou Hospital, Okayama, Japan	No age restriction in sampling	nr	54	54.5	1) 30-day mortality, 2) All-cause mortality
Henshke-Bar-Meir, R., *et al.* 2006. (80)	Third/fourth/fifth generation cephalosporins or ESBLs	Cohort	Cohort	Retrospective	Isreal	High	2000 - 2001	1	University-affiliated general hospital, Jerusalem	No age restriction in sampling	nr	nr	nr	1) All-cause mortality
Ho, P. L., *et al.* 2002. (64)	Third/fourth/fifth generation cephalosporins or ESBLs	Case-control	Cohort	Retrospective	Hong Kong	High	01/1996 - 12/1998	1	Tertiary care hospital, Queen Mary Hospital, Hong Kong	No age restriction in sampling	60	60	60	1) 30-day mortality, 2) All-cause mortality
Hsieh, C. J.,S *et al.* 2010. (68)	Third/fourth/fifth generation cephalosporins or ESBLs	Not reported	Cohort	Retrospective	Taiwan	High	01/2005 - 12/2006	1	Chang Gung Memorial Hospital, Kaohsiung, Taiwan	No age restriction in sampling	58.4	78.9	57.4	1) 30-day mortality, 2) All-cause mortality, 3) Post-infection length of hospital stay
Huang, Y. Y., *et al.* 2018. (97)	Third/fourth/fifth generation cephalosporins or ESBLs	Case-control	Cohort	Retrospective	Canada	High	01/2011 - 06/2013	1	Surrey Memorial Hospital, Surrey, BC	≥ 19	59.1	43.1	66.9	1) All-cause mortality, 2) Length of hospital stay
Huotari, K., *et al.* 2003. (54)	Quinolones	Case-control	Cohort	Retrospective	Finland	High	01/1997 - 12/1999	1	Helsinki University Central Hospital, Helsinki, Finland	≥ 18	nr	58.8	69.6	1) 30-day mortality, 2) All-cause mortality, 3) Length of hospital stay
Jeon, J. H., *et al.* 2012. (110)	Quinolones	Observational	Cohort	Prospective	Republic of Korea	High	03/2005 - 12/2008	1	Seoul National University Bundang Hospital, Seoul, Korea	≥ 15	100	100	100	1) Treatment failure, 2) Length of stay
Kang, C. I., *et al.* 2010. (51)	Third/fourth/fifth generation cephalosporins or ESBLs	Cohort	Cohort	Retrospective	Republic of Korea	High	10/2006 - 09/2007 and 09/2008 - 04/2009	≥ 3	18 hospitals in South Korea (nationwide surveillance)	No age restriction in sampling	nr	51.2	63.6	1) 30-day mortality, 2) All-cause mortality, 3) Treatment failure
Kang, C. I., *et al.* 2011. (52)	Third/fourth/fifth generation cephalosporins or ESBLs	Surveillance	Cohort	Retrospective	Republic of Korea	High	10/2006 - 09/2007 and 10/2008 - 04/2009	≥ 3	18 hospitals in South Korea (nationwide surveillance)	≥ 16	nr	nr	nr	1) 30-day mortality, 2) All-cause mortality
Kang, C. I., *et al.* 2012. (71)	Third/fourth/fifth generation cephalosporins or ESBLs	Case-control	Cohort	Retrospective	Republic of Korea	High	09/2010- 06/2011	2	Samsung Medical Center (Tertiary), Seoul, Korea and Samsung Changwon Hospital (community), Changwon, Korea	>15 years	nr	64.8	79.6	1) 30-day mortality, 2) All-cause mortality

Kaya, O., *et al.* 2013. (46)	Third/fourth/fifth generation cephalosporins or ESBLs	Not reported	Cohort	Retrospective	Turkey	Upper-middle	01/2007 - 10/2011	1	Sleyman Demirel University Research Hospital, Isparta, Turkey	≥ 18	48.7	45.5	50.7	1) All-cause mortality
Khan, F. Y., *et al.* 2010. (82)	Third/fourth/fifth generation cephalosporins or ESBLs	observational, surveillance	Cohort	Prospective	Qatar	High	07/2007 - 06/2008	≥ 3	3 Hospitals that comprise the Hamad Medical Corporation in Qatar	No age restriction in sampling	nr	nr	nr	1) All-cause mortality
Kim, J., *et al.* 2014. (79)	Quinolones	Case-control	Cohort	Retrospective	Republic of Korea	High	06/2000 - 08/2011	1	Samsung Medical Center, Seoul, Korea	≥ 18	nr	26.1	28.6	1) 30-day mortality, 2) All-cause mortality
Kim, S. H., *et al.* 2013. (73)	Third/fourth/fifth generation cephalosporins or ESBLs	Not reported	Cohort	Retrospective	Republic of Korea	High	01/2007 - 12/2008	1	Hematology ward of a tertiary hospital in Seoul, South Korea	No age restriction in sampling	nr	nr	nr	1) 30-day mortality, 2) All-cause mortality
Komatsu, Y., *et al.* 2018. (98)	Third/fourth/fifth generation cephalosporins or ESBLs	Case-control	Cohort	Retrospective	Japan	High	01/2008 - 05/2013	1	Nara Medical University, Tertiary Care Hospital, Nara, Japan	≥ 18	nr	36.7	56.5	1) All-cause mortality
Lambert, M. L., *et al.* 2011. (53)	Third/fourth/fifth generation cephalosporins or ESBLs	Cohort	Cohort	Prospective	10 European Countries	High	01/2005 - 12/2008	≥ 3	10 European countries - Austria, Belgium, Croatia, France, Italy, Latvia, Portugal, Slovakia, Scotland and Spain 537 ICUs	No age restriction in sampling	nr	nr	nr	1) All-cause mortality, 2) Post-infection length of stay
Laupland, K. B., *et al.* 2008. (105)	Quinolones	Cohort	Cohort	Prospective	Canada	High	01/2000 - 12/2006	≥ 3	Calgary Health Region, population-based study	No age restriction in sampling	nr	nr	nr	1) All-cause mortality
Lee, H.,*et al.* 2018. (99)	Third/fourth/fifth generation cephalosporins or ESBLs	Case-control	Cohort	Retrospective	Republic of Korea	High	06/2015 - 12/2006	1	Tertiary hospital	≥ 18	nr	75	nr	1) All-cause mortality, 2) Length of stay
Lee, S., *et al.* 2014. (89)	Third/fourth/fifth generation cephalosporins or ESBLs	Cohort	Cohort	Retrospective	Republic of Korea	High	01/2009 - 12/2011	1	secondary care hospital, Daegu, South Korea	No age restriction in sampling	nr	85	90	1) All-cause mortality, 2) Treatment failure, 3) Length of stay
Leistner, R., *et al.* 2014. (112)	Third/fourth/fifth generation cephalosporins or ESBLs	Case-control	Cohort	Retrospective	Germany	High	01/2008 - 12/2010	1	Charite University Hospital, Berlin, Germany	No age restriction in sampling	nr	38	38	1) Post-infection length of stay, 2) Healthcare system costs
Leistner, R., *et al.* 2014. (90)	Third/fourth/fifth generation cephalosporins or ESBLs	Cohort	Cohort	Retrospective	Germany	High	01/2008 - 12/2010	1	Charite University Hospital, Berlin, Germany	No age restriction in sampling	nr	36	47	1) All-cause mortality, 2) Length of stay

Leistner, R., *et al.* 2014. (91)	Third/fourth/fifth generation cephalosporins or ESBLs	Cohort	Cohort	Retrospective	Germany	High	01/2008 - 12/2011	1	Charite University Hospital, Berlin, Germany	No age restriction in sampling	47.2	nr	nr	1) All-cause mortality
Lim, C., *et al.* 2016. (45)	Multidrug resistance	Not reported	Cohort	Retrospective	Thailand	Upper-middle	01/2004 - 12/2010	≥3	9 provincial hospitals in Northeast Thailand	No age restriction in sampling	nr	nr	nr	1) 30-day mortality, 2) All-cause mortality
Ma, J., *et al.* 2017. (48)	Third/fourth/fifth generation cephalosporins or ESBLs	Case-control	Cohort	Retrospective	China	Upper-middle	04/2012 - 07/2015	1	Hematological center at The First Affiliated Hospital of Zhengzhou University	≥ 14	nr	50	58.1	1) 30-day mortality, 2) All-cause mortality
Martelius, T., *et al.* 2016. (56)	Third/fourth/fifth generation cephalosporins or ESBLs	Surveillance	Cohort	Prospective	Finland	High	01/1999 - 12/2013	≥ 3	Started with 4 hospitals in 1999 and by 2013 there were 17 hospitals representing 12 health districts	No age restriction in sampling	nr	36.8	42.4	1) 30-day mortality, 2) All-cause mortality
Maslikowska, J. A., *et al.* 2016. (93)	Third/fourth/fifth generation cephalosporins or ESBLs	Case-control	Cohort	Retrospective	Canada	High	06/18/2010 - 04/05/2013	1	Sunnybrook Health Sciences Centre	No age restriction in sampling	nr	nr	rn	1) All-cause mortality, 2) Bacterium-attributable mortality, 3) Treatment failure,
Melzer, M., *et al.* 2007. (65)	Third/fourth/fifth generation cephalosporins or ESBLs	Not reported	Cohort	Prospective	UK	High	06/2003 - 11/2005	1	King George Hospital in Essex	≥ 16	61.9	39.1	65.3	1) 30-day mortality, 2) All-cause mortality, 3) Post-infection length of hospital stay
Namikawa, H., *et al.* 2017. (96)	Third/fourth/fifth generation cephalosporins or ESBLs	Not reported	Cohort	Retrospective	Japan	High	01/2011 - 06/2015	1	Osaka City University Hospital	No age restriction in sampling	nr	61.3	53.1	1) All-cause mortality
Nicolas-Chanoine, M. H., *et al.* 2012. (86)	Third/fourth/fifth generation cephalosporins or ESBLs	Case-case-control	Cohort	Prospective	France	High	11/2008-06/2009	≥ 3	10 hospitals in the Assistance Publique-Hopitaux de Paris system	No age restriction in sampling	nr	65	61	1) All-cause mortality
Nussbaum, A., et al. 2018. (88)	Third/fourth/fifth generation cephalosporins or ESBLs	Cohort	Cohort	Retrospective	USA	High	01/2007-12/2009	1	New York Hospital Queens	No age restriction in sampling	nr	44	42	1) All-cause mortality, 2) Length of hosptial stay
Ortega, M., *et al.* 2009. (58)	Third/fourth/fifth generation cephalosporins or ESBLs	Surveillance	Cohort	Prospective	Spain	High	01/1991 - 12/2007	1	Hospital Clinic in Barcelona	No age restriction in sampling	54	56	54	1) 30-day mortality, 2) All-cause mortality
Ortega, M., *et al.* 2009. (58)	Quinolones	Surveillance	Cohort	Prospective	Spain	High	01/1991 - 12/2007	1	Hospital Clinic in Barcelona	No age restriction in sampling	54	49	55	1) 30-day mortality, 2) All-cause mortality

Park, S. H., *et al.* 2011. (69)	generation cephalosporins or ESBLs	Cohort	Cohort	Retrospective	Republic of Korea	High	01/2005 - 12/2012	1	Daejeon St. Mary's Hospital	≥ 18	nr	76	70	1) 30-day mortality, 2) All-cause mortality
Park, S. H., *et al.* 2015. (94)	Third/fourth/fifth generation cephalosporins or ESBLs	Case-control	Cohort	Retrospective	Republic of Korea	High	01/2007 - 12/2013	1	Daejeon St. Mary's Hospital	> 15	nr	88	93.8	1) All-cause mortality, 2) Treatment failure, 3) Length of hospital stay
Parveen, A., *et al.* 2015. (39)	Multidrug resistance	Not reported	Cohort	Retrospective	Pakistan	Lower-middle	12/2012 - 11/2013	1	Shaukat Khanum Memorial Cancer Hospital and Research Centre, Lahore, Pakistan	No age restriction in sampling	34.8	25.5	41.2	1) 30-day mortality, 2) All-cause mortality
Pena, C., *et al.* 2008. (66)	Third/fourth/fifth generation cephalosporins or ESBLs	Cohort	Cohort	Retrospective	Spain	High	01/1996 - 12/2003	1	Hospital Universitari de Bellvitge, hospital for adult patients	≥ 18	nr	46	nr	1) 30-day mortality, 2) All-cause mortality
Peralta, G., *et al.* 2007. (57)	Third/fourth/fifth generation cephalosporins or ESBLs	Cohort	Cohort	Retrospective	Spain	High	01/1997 - 06/2005	1	Sierralllana Hospital in Torrelavega, Spain	≥ 14	48.9	nr	nr	1) All-cause mortality
Peralta, G., *et al.* 2007. (57)	Quinolones	Cohort	Cohort	Retrospective	Spain	High	01/1997 - 06/2005	1	Sierrallana Hospital, Torrelavega, Spain	≥ 14	48.9	nr	nr	1) All-cause mortality
Riu, M., *et al.* 2016. (55)	Multidrug resistance	Not reported	Cohort	Retrospective	Spain	High	01/2005 - 12/2012	1	Hospital del Mar, Barcelona	No age restriction in sampling	nr	nr	nr	1) Healthcare system costs
Rodriguez-Bano, J. *et al.* 2010. (83)	Third/fourth/fifth generation cephalosporins or ESBLs	Case-control	Cohort	Prospective	Spain	High	10/2004 - 01/2006	≥ 3	13 tertiary hospitals	≥ 14	nr	44	56	1) All-cause mortality
Shin, J., *et al.* 2012. (106)	Quinolones	Cohort	Cohort	Retrospective	Republic of Korea	High	01/2008- 12/2008	≥ 3	14 hospitals in South Korea	No age restriction in sampling	nr	100	98.8	1) All-cause mortality, 2) Treatment failure, 3) Length of hospital stay
Soraas, A., *et al.* 2014. (109)	Third/fourth/fifth generation cephalosporins or ESBLs	Population-based	Cohort	Prospective	Norway	High	02/2009- 05/2012	1	Vestre Viken Hospital Trust, Department of Medical Microbiology	≥ 18	87	nr	nr	1) Treatment failure
Thaden, J. T., *et al.* 2017. (63)	Multidrug resistance	Cohort	Cohort	Prospective	USA	High	01/2009 - 12/2015	1	Duke University Medical Centre	≥ 18	nr	nr	nr	1) Healthcare system costs
Trecarichi, E. M., *et al.* 2009. (59)	Third/fourth/fifth generation cephalosporins or ESBLs	Cohort	Cohort	Prospective	Italy	High	01/2000- 12/2007	1	The Catholic University Hospital, Rome, Italy	≥ 17	43.6	50	38.9	1) 30-day mortality, 2) All-cause mortality

Trecarichi, E. M., *et al.* 2009. (59)	Quinolones	Cohort	Cohort	Retrospective	Italy	High	01/2000-12/2007	1	Catholic University Hospital, Rome, Italy	≥ 17	43.6	46.2	39.2	1) 30-day mortality, 2) All-cause mortality
Tumbarello, M., *et al.* 2010. (84)	Third/fourth/fifth generation cephalosporins or ESBLs	Cohort	Cohort	Retrospective	Italy	High	01/2006-12/2006	1	The Catholic University Hospital, Rome, Italy	≥ 18	51.5	51.4	51.6	1) All-cause mortality, 2) Treatment failure, 3) Post-infection length of hospital stay, 4) Healthcare system costs
Uzodi, A. S., *et al.* 2017. (62)	Multidrug resistance	Cohort	Cohort	Retrospective	USA	High	01/2012-12/2012	1	Mayo Clinic Children's Hospital, Rochester, MN	≤ 18	nr	nr	nr	1) All-cause mortality, 2) Treatment failure, 3) Length of hospital stay
Van Aken, S., *et al.* 2014. (92)	Third/fourth/fifth generation cephalosporins or ESBLs	Case-control	Cohort	Retrospective	Sweden	High	01/2011-09/2012	≥ 3	All hospitals in Skane county, did not provide the specific number	No age restriction in sampling	49	42.9	52.1	1) All-cause mortality, 2) Length of hospital stay
Yip, T., *et al.* 2006. (81)	Third/fourth/fifth generation cephalosporins or ESBLs	Not reported	Cohort	Retrospective	Hong Kong	High	10/1994 - 08/2003	1	Tung Wah Hospital, Hong Kong	No age restriction in sampling	nr	72.7	64.9	1) All-cause mortality, 2) Treatment failure
Yoon, E. J., *et al.* 2018. (61)	Third/fourth/fifth generation cephalosporins or ESBLs	Observational	Cohort	Prospective	Republic of Korea	High	05/2016 - 04/2017	≥ 3	Six general hospitals in South Korea	No age restriction in sampling	60.3	nr	nr	1) 30-day mortality, 2) All-cause mortality
Yoon, E. J., *et al.* 2018. (61)	Quinolones	Observational	Cohort	Prospective	Republic of Korea	High	05/2016 - 04/2017	≥ 3	Six general hospitals in South Korea	No age restriction in sampling	60.3	nr	nr	1) 30-day mortality, 2) All-cause mortality
Yoon, E. J., *et al.* 2018. (61)	Multidrug resistance	Observational	Cohort	Prospective	Republic of Korea	High	05/2016 - 04/2017	≥ 3	Six general hospitals in South Korea	No age restriction in sampling	60.3	nr	nr	1) 30-day mortality, 2) All-cause mortality
