# Supplementary material for: Evaluation of the health and healthcare system burden due to antimicrobial-resistant Escherichia coli infections in humans: a systematic review and meta-analysis
Source: Antimicrob Resist Infect Control. 2020 Dec 10;9:200. doi: 10.1186/s13756-020-00863-x (PMC7726913; doi:10.1186/s13756-020-00863-x)
Supplement: Supplementary file 6 — Additional file 6: Characteristics of E. coli infections in studies included in the systematic review [file 13756_2020_863_MOESM6_ESM.pdf]

**Additional file 6 - Characteristics of *E. coli* infections in studies included in systematic review evaluating whether the measures of health or healthcare system burden increase in humans with antimicrobial-resistant *E. coli* infections**

| Resistant (R) and Susceptible (S) Group Details | | | | | | | | Type and Source of Infection | | | BoD / Overall |
|---|---|---|---|---|---|---|---|---|---|---|---|
| Citation (Reference # in manuscript) | Type of Resistance | Underlying common disease processes or common characteristics | Definition of cases with R | Number of cases with R | Definition of cases with S | Number of cases with S | Selection of R and S groups | Types of infections in study | Source/ timing of infections | Duration of follow-up | BoD Outcome |
| Abernethy, J. K., *et al.* 2015. (50) | Third/fourth/fifth generation cephalosporins or ESBLs | n/a | 3GCR EC BSI - 3CGR defined by resistance to cefotaxime and/or ceftazidime | 1838 | 3GCS EC BSI | 16641 | No selection all EC BSI during study period, but some were not tested or had missing data | Blood stream infection | Community-associated; Healthcare-associated; Nosocomial | 30-days | 1) 30-day mortality; 2) All-cause mortality |
| Abernethy, J. K., *et al.* 2015. (50) | Quinolones | n/a | CIP-R EC BSI - Ciprofloxacin resistance | 3647 | CIP-S EC BSI | 16828 | No selection all EC BSI during study period, but some were not tested or had missing data | Blood stream infection | Community-associated; Healthcare-associated; Nosocomial | 30-days | 1) 30-day mortality; 2) All-cause mortality |
| Al-Otaibi, F. E., *et al.* 2013. (87) | Third/fourth/fifth generation cephalosporins or ESBLs | n/a | ESBL EC UTI | 113 | non-ESBL EC UTI | 226 | No mention of selection | Urinary tract infection | Not reported | nr | 1) All-cause mortality |
| Alvarez-Uria, G., *et al.* 2012. (37) | Third/fourth/fifth generation cephalosporins or ESBLs | n/a - all community-acquired the definition is consistent with community-associated | 3GCR EC CA infection - 3GCR was based on resistance to cefotaxime | 154 | 3GCS EC CA infection | 40 | No selection all EC CA infections during study period were included | Blood stream infection; Urinary tract infection; normally sterile site infection; wound infection; pneumonia | Community-associated | 21 days | 1) All-cause mortality |
| Anunnatsiri, S., *et al.* 2012. (42) | Third/fourth/fifth generation cephalosporins or ESBLs | n/a | ESBL EC BSI | 32 | non-ESBL EC BSI | 113 | No selection, all EC BSI during study period that were not polymicrobial, transferred to another hospital and had to be the first BSI for each patient. | Blood stream infection | Community-associated; Heathcare-associated; nosocomial | in-hospital | 1) All-cause mortality, 2) Treatment failure, 3) Post-infection length of hospital stay |

| | | | | | | | | | | | |
|---|---|---|---|---|---|---|---|---|---|---|---|
| Apisarnthana-rak, A., *et al.* 2008. (43) | Third/fourth/fifth generation cephalosporins or ESBLs | All patients were community-onset (definition consistent with community-associated/acquired) | ESBL EC CO infection | 46 | non-ESBL EC CO infection | 138 | No selection of ESBL EC, all that occurred during study period were included. The non-ESBL EC controls were matched 3:1 to ESBLs. Matched based on site of infection, hospital unit and day of admission (+/- 7 days). | Blood stream infection; Urinary tract infection; skin/soft tissue infection; pneumonia | Community-associated | in-hospital | 1) All-cause mortality, 2) Length of hospital stay, 3) Healthcare system costs |
| Artero, A., *et al.* 2017. (95) | Third/fourth/fifth generation cephalosporins or ESBLs | All community-onset pyelonephritis or urosepsis in elderly | ESBL EC pyelonephritis or urosepsis | 85 | non-ESBL EC pyelonephritis or urosepsis | 225 | No selection all community-onset EC pyelonephritis or urosepsis in elderly during study period | pyelonephritis or urosepsis | Community-associated; Healthcare-associated | In-hospital | 1) All-cause mortality, 2) Length of hospital stay |
| Bert, F., *et al.* 2008. (103) | Quinolones | All patients had cirrhosis | Ciprofloxacin-resistant EC SPB | 11 | Ciprofloxacin-resistant EC SPB | 60 | No selection, all EC SBP cases during the study period | Spontaneous bacterial peritonitis | Community-associated; Healthcare-associated; nosocomial | in-hospital | 1) All-cause mortality |
| Bollestad, M., *et al.* 2018. (107) | Third/fourth/fifth generation cephalosporins or ESBLs | all treated with oral pivmecillinam, women with community-acquired UTI | monoculture of ESBL-EC in urine | 88 | monoculture of non-ESBL-EC in urine | 74 | Cases and controls identified at the micro labs, then registered by primary physicians. Initially controls matched to cases on age (+5 yr) but then that was stopped after 27 matched pairs. | Urinary tract infection | Community-associated | up to 2 weeks after end of treatment | 1) Treatment failure |
| Camins, B. C., *et al.* 2011. (78) | Quinolones | n/a | Fluoroquinolone-resistant EC bacteremia | 93 | Fluoroquinolone-susceptible EC bacteremia | 93 | All FQ-R EC BSI during the study period were included. The FQ-S E BSI controls were matched 1:1 to FQ-R. They were randomly matched by year of infection. | Blood stream infection | Community-associated; Healthcare-associated; Nosocomial | in-hospital up to 30 days | 1) 30-day mortality, 2) All-cause mortality, 3) Treatment failure, 4) Length of hospital stay |
| Can, F., *et al.* 2015. (41) | Third/fourth/fifth generation cephalosporins or ESBLs | n/a | ESBL EC UTI | 70 | non-ESBL EC UTI | 224 | No selection, all EC UTI during study period | Urinary tract infection | Not reported | 10 days | 1) Treatment failure |
| Can, F., *et al.* 2015. (41) | Quinolones | n/a | Quinolone-resistant EC UTI | 114 | Quinolone-susceptible EC UTI | 180 | No selection, all EC UTI during study period | Urinary tract infection | Not reported | 10 days | 1) Treatment failure |

| | | | | | | | | | | | |
|---|---|---|---|---|---|---|---|---|---|---|---|
| Can, F., *et al.* 2015. (41) | Multidrug resistance | n/a | MDR EC UTI - MRD defined as resistance to 3 or more different groups of antibiotics (B lactams, aminoglycosides, quinolones, TMP-SMX) | 116 | non-MDR EC UTI | 178 | No selection, all EC UTI during study period | Urinary tract infection | Not reported | 10 days | 1) Treatment failure |
| Cereto, F., *et al*. 2008. (104) | Quinolones | All patients had cirrhosis and were treated emipirically with cefotaxime or ceftriaxone | quinolone-resistant EC SBP, defined by resistance to ciprofloxacin | 18 | quinolone-susceptible EC SBP | 29 | no selection, all EC SBP cases during study period | Spontaneous bacterial peritonitis | Not reported | 3 months | 1) All-cause mortality, 2) Treatment failure |
| Cereto, F., *et al*. 2008. (104) | Quinolones | All patients had cirrhosis of the liver | quinolone-resistant EC SBP (spontaneous bacterial peritonitis) | 12 | quinolone-susceptible EC SBP | 35 | No selection all cases of EC SBP | Spontaneous bacterial peritonitis | Not reported | in-hospital | 1) All-cause mortality |
| Chakraborty, A., *et al*. 2012. (38) | Third/fourth/fifth generation cephalosporins or ESBLs | n/a | ESBL extra-intestinal EC infection | 132 | non-ESBL extra-intestinal EC infection | 68 | No selection all EC infections included as long as they met criteria excluded if received anti-microbials within 1 month, asymptomatic UTI, polymicrobial infections | Blood stream infection; Urinary tract infection; Wound infection, pneumonia, IV device infection, meningitis | Community-associated; Healthcare-associated; Nosocomial | not reported | 1) All-cause mortality, 2) Treatment failure |
| Chauhan, S., 2015. (40) | Third/fourth/fifth generation cephalosporins or ESBLs | n/a | ESBL-producing E. coli invasive infections | 85 | non-ESBL-producing E. coli invasive infections | 85 | All cases in time period, and the control closest in time that met eligibility criteria | Invaisive infections in hospital patients no further details | Nosocomial only type mentioned. | nr | 1) Treatment failure, 2) Length of stay |
| Cheong, H. J., *et al.* 2001. (101) | Quinolones | All had to have >= 2 positive blood samples | quinolone resistant EC bacteremia | 40 | quinolone susceptible EC bacteremia | 80 | All QR EC during study period and controls matched 2:1 to cases. Matched based on date of positive culture result | Blood stream infection | Community-associated; Healthcare-associated; Nosocomial | in-hospital | 1) All-cause mortality |
| Cheong, H. S., *et al.* 2007. (60) | Third/fourth/fifth generation cephalosporins or ESBLs | All community-onset | ceftriaxone-resistant EC bacteremia | 19 | ceftriaxone-susceptible EC bacteremia | 489 | no selection | Blood stream infection | Community-associated; Healthcare-associated | 30-day | 1) 30-day mortality, 2) All-cause mortality |
| Cheong, H. S., *et al.* 2007. (60) | Quinolones | All community-onset | ciprofloxacin-resistant EC bacteremia | 132 | ciprofloxacin-susceptible EC bacteremia | 376 | no selection | Blood stream infection | Community-associated; Healthcare-associated | 30-day | 1) 30-day mortality, 2) All-cause mortality |

| | | | | | | | | | | | |
|---|---|---|---|---|---|---|---|---|---|---|---|
| Cheong, H. S., *et al.* 2007. (60) | Multidrug resistance | All community-onset | MDR EC bacteremia MDR defined as not susceptible to three or more of the following antimicrobial agents: ampicillin, amikacin or gentamicin or tobramycin, aztreonam, cefotaxime or ceftriaxone or ceftazidime or cefepime, ciprofloxacin, imipenem, piperacillin/ tazobactam and trimethoprim/ sulfamethoxazole | 137 | non-MDR EC bacteremia | 371 | no selection | Blood stream infection | Community-associated; Healthcare-associated | 30-day | 1) 30-day mortality, 2) All-cause mortality |
| Cornejo-Juarez, P., *et al.* 2012. (44) | Third/fourth/fifth generation cephalosporins or ESBLs | all patients have a hematologic malignancy | ESBL EC BSI | 100 | non-ESBL EC BSI | 100 | All ESBL EC BSI included and matched to 1 non-ESBL EC BSI. Controls matched to be +/- 30 day prior to the date of isolate for the ESBL EC BSI case. | Blood stream infection | Community-associated; Healthcare-associated; Nosocomial | 60-days | 1) All-cause mortality, 2) Bacterium-attributable mortality |
| Courpon-Claudinon, A. *et al.* 2011. (85) | Third/fourth/fifth generation cephalosporins or ESBLs | n/a | 3GC-R EC bacteremia | 39 | 3GC-S EC bacteremia | 1012 | no selection | Blood stream infection | Community-associated; Healthcare-associated; Nosocomial | in-hospital | 1) All-cause mortality |
| de Kraker, M. E., *et al.* 2011. (49) | Third/fourth/fifth generation cephalosporins or ESBLs | n/a | 3GC-R EC BSI (REC) | 111 | 3GC-S EC BSI (SEC) | 1110 | all REC and SEC that could be matched to controls with no EC BSI Note: paper describes how both cohorts are matched with controls - these controls do not have E.coli BSI. | Blood stream infection | Community-associated; Healthcare-associated; Nosocomial | 30-days | 1) 30-day mortality, 2) All-cause mortality, 3) Post-infection length of stay |
| Denis, B., *et al.* 2015. (74) | Third/fourth/fifth generation cephalosporins or ESBLs | n/a - the hospital has major clinical areas in HIV, hematology and oncology | ESBL EC bacteremia | 41 | non-ESBL EC bacteremia | 41 | All ESBL EC bacteremia cases during study period. Controls (non-ESBL EC bacteremia) were matched 1:1 to cases, matched to the closest date of a case. | Blood stream infection | Community-associated; Healthcare-associated; Nosocomial | 30-day | 1) 30-day mortality, 2) All-cause mortality, 3) Length of stay |

| | | | | | | | | | | | |
|---|---|---|---|---|---|---|---|---|---|---|---|
| Eom, J. S., *et al.* 2002. (77) | Quinolones | n/a | quinolone-resistant EC UTI (used ciprofloxacin to determine) | 60 | quinolone-susceptible EC UTI (used ciprofloxacin to determine) | 80 | Controls were selected for each case according to the nearest date of a positive urine culture | Urinary tract infection | Community-associated; Healthcare-associated; Nosocomial | 30-day | 1) 30-day mortality, 2) All-cause mortality, 3) Treatment failure |
| Esteve-Palau, E., *et al.* 2015. (75) | Third/fourth/fifth generation cephalosporins or ESBLs | n/a | symptomatic UTI caused by ESBL-EC | 60 | symptomatic UTI caused by non-ESBL-EC | 60 | All ESBL EC UTI during study period and controls matched 1:1 to cases. Matched on sex, age, and date of admission. | Urinary tract infection | Community-associated; Healthcare-associated | 30-days | 1) 30-day mortality, 2) All-cause mortality, 3) Treatment failure, 4) Length of stay, 5) Healthcare system costs |
| Fan, N. C., *et al.* 2014. (108) | Third/fourth/fifth generation cephalosporins or ESBLs | all community-onset | ESBL EC UTI | 104 | non-ESBL EC UTI | 208 | All cases during study period, match 1:2 to controls by age and sex | Urinary tract infection | All community-onset - Community-associated; Healthcare-associated | not reported | 1) Treatment failure, 2) Length of stay |
| Feng, X. R., *et al.* 2014. (47) | Third/fourth/fifth generation cephalosporins or ESBLs | All patients on stable continuous ambulatory peritoneal dialysis (CAPD) | ESBL EC peritonitis | 32 | non-ESBL EC peritonitis | 58 | No selection all EC peritonitis in CAPD patients during the study period | Peritonitis | Not reported | After enrollment patients, were followed up until the cessation of PD (transfer to hemodialysis, transplantation or death) or the end of the study | 1) Bacterium-attributable mortality, 2) Treatment failure |
| Freeman, J. T., *et al.* 2012. (70) | Third/fourth/fifth generation cephalosporins or ESBLs | n/a | ESBL EC bacteremia | 16 | non- ESBL EC bacteremia | 16 | All cases during the study period, controls were matched 1:1 to cases. Matching criteria: species of organism, type of blood culture, timing of bacteremia, and temporally closest to the case | Blood stream infection | Not reported | 30-days | 1) 30-day mortality, 2) All-cause mortality |
| Gagliotti, C., *et al.* 2008. (111) | Quinolones | All female, community-acquired and at low risk of treatment failure / relapse | ciprofloxacin resistant EC UTI - community acquired | 900 | ciprofloxacin susceptible EC UTI - community acquired | 6820 | No selection all community acquired EC UTI | Urinary tract infection | Community-associated; Healthcare associated (all community-acquired) | 30-days | 1) Treatment failure |
| Garau, J., *et al.* 1999. (100) | Quinolones | n/a | quinolone resistant EC bacteremia (ciprofloxacin) | 70 | quinolone susceptible EC bacteremia (ciprofloxacin) | 502 | No selection, all EC bacteremia during the study period | Blood stream infection | Community-associated; Healthcare associated; Nosocomial | in hospital | 1) All-cause mortality |

| | | | | | | | | | | | |
|---|---|---|---|---|---|---|---|---|---|---|---|
| Gudiol, C., *et al.* 2010. (67) | Third/fourth/fifth generation cephalosporins or ESBLs | All cancer patients | ESBL EC bacteremia | 17 | non-ESBL EC bacteremia | 118 | No selection all EC bacteremia during study period | Blood stream infection | Called it healthcare acquisition with no definition | 30-days | 1) 30-day mortality, 2) All-cause mortality |
| Ha, Y. E., *et al.* 2013. (72) | Third/fourth/fifth generation cephalosporins or ESBLs | All have cancer | ESBL EC bacteremia | 95 | non-ESBL EC bacteremia | 255 | No selection all EC bacteremia during study period | Blood stream infection | Community-associated; Healthcare associated; Nosocomial | 30-day | 1) 30-day mortality, 2) All-cause mortality |
| Haruki, Y., *et al.* 2018. (76) | Third/fourth/fifth generation cephalosporins or ESBLs | critically ill patients from ICU | ESBL EC bacteremia | 24 | non-ESBL EC bacteremia | 77 | No selection, all EC bacteremia during study period | Blood stream infection | Community-assoicated; healthcare-assoicated; Nosocomial | 28-day | 1) 30-day mortality, 2) All-cause mortality |
| Henshke-Bar-Meir, R., *et al.* 2006. (80) | Third/fourth/fifth generation cephalosporins or ESBLs | n/a | ESBL enterobacteriaceae bacteremia | 39 | non-ESBL enterobacteriaceae bacteremia | 119 | No selection reported, no matching but aimed for ratio of 3 controls for each case, so there was selection | Blood stream infection | Not reported | in-hospital | 1) All-cause mortality |
| Ho, P. L., *et al.* 2002. (64) | Third/fourth/fifth generation cephalosporins or ESBLs | n/a | ESBL EC BSI | 50 | non-ESBL EC BSI | 100 | 74 episodes of ESBL EC BSI during study - 24 excluded (4 - polymicrobial and 20 incomplete medical records). Non-ESBL matched 2:1. Matched on specialty, age (+/- 10 yr), sex and closest date of isolation to ESBL. | Blood stream infection | Community-associated; Healthcare associated; Nosocomial | 30 days | 1) 30-day mortality, 2) All-cause mortality |
| Hsieh, C. J.,S *et al.* 2010. (68) | Third/fourth/fifth generation cephalosporins or ESBLs | n/a | ESBL EC community-onset bacteremia | 19 | non-ESBL EC community-onset bacteremia | 385 | No sampling, all EC community-onset bacteremia without missing data in the medical record during the study period | Blood stream infection | Community-associated; Healthcare-associated | 30-day | 1) 30-day mortality, 2) All-cause mortality, 3) Post-infection length of hospital stay |
| Huang, Y. Y., *et al.* 2018. (97) | Third/fourth/fifth generation cephalosporins or ESBLs | All patients had urosepsis (bacteremia confirmed from a urinary source and had two criteria for systemic inflammatory response syndrome) | ESBL EC Urosepsis | 58 | non-ESBL EC Urosepsis | 118 | All ESBL EC urosepsis cases during study period and random selection of 118 non-ESBL EC urosepsis controls during study period | Blood stream infection (All urosepsis) | Community-associated; Healthcare-associated; Nosocomial | in-hospital | 1) All-cause mortality, 2) Length of hospital stay |

| | | | | | | | | | | | |
|---|---|---|---|---|---|---|---|---|---|---|---|
| Huotari, K., *et al.* 2003. (54) | Quinolones | All nosocomial | nosocomial quinolone resistant EC infection | 51 | nosocomial quinolone susceptible EC infection | 102 | All nosocomial QREC cases from that hospital during the study period which had two appropriate controls. Two controls for each case, matched based on having the same type of infection. | Urinary tract infection, UTI with bacteremia, surgical site infection, skin and soft tissue, pneumonia | Nosocomial | 30-day | 1) 30-day mortality, 2) All-cause mortality, 3) Length of hospital stay |
| Jeon, J. H., *et al.* 2012. (110) | Quinolones | All from communities with a high prevalence of fluoroquinolone resistance and were treated with empirical ciprofloxacin | Ciprofloxacin-resistant EC acute uncomplicated pyelonephritis | 39 | Ciprofloxacin-susceptible EC acute uncomplicated pyelonephritis | 216 | No selection, all EC acute uncomplicated pyelonephritis that consented and met criteria | Acute uncomplicated pyelonephritis | Not reported | 14 to 21-days after completion of treatment but not for all patients | 1) Treatment failure, 2) Length of stay |
| Kang, C. I., *et al.* 2010. (51) | Third/fourth/fifth generation cephalosporins or ESBLs | all community-onset bacteremia | ESBL EC community-onset bacteremia | 82 | non-ESBL EC community-onset bacteremia | 783 | no selection | Blood stream infection | Community-associated; Healthcare-associated | 30-days | 1) 30-day mortality, 2) All-cause mortality, 3) Treatment failure |
| Kang, C. I., *et al.* 2011. (52) | Third/fourth/fifth generation cephalosporins or ESBLs | all bacteremic intra-abdominal infections | broad-spectrum cephalosporin-resistant EC bacteremic intra-abdominal infections. Broad-spectrum cephalosporin-resistance was defined as in vitro resistance to cefotaxime ceftriaxone or ceftazidime | 29 | broad-spectrum cephalosporin-susceptible EC bacteremic intra-abdominal infections | 175 | no selection | Blood stream infection | Community-associated; Healthcare-associated; Nosocomial | 30-days | 1) 30-day mortality, 2) All-cause mortality |
| Kang, C. I., *et al.* 2012. (71) | Third/fourth/fifth generation cephalosporins or ESBLs | all community-onset and from culture drawn in ER or outpatient clinics | ESBL EC | 108 | non-ESBL EC | 108 | 1:1, controls were randomly selected from a group of outpatients, the sample had to be cultured at the same lab during the week following and they were matched according to the culture specimen and acquisition unit | All types of EC infections | Community-associated; Healthcare-associated | 30-days | 1) 30-day mortality, 2) All-cause mortality |
| Kaya, O., *et al.* 2013. (46) | Third/fourth/fifth generation cephalosporins or ESBLs | n/a | ESBL EC BSI | 44 | non-ESBL EC BSI | 69 | No selection, all EC BSI during study period | Blood stream infection | Community-associated; Healthcare-associated; Nosocomial | in-hospital | 1) All-cause mortality |

| | | | | | | | | | | | |
|---|---|---|---|---|---|---|---|---|---|---|---|
| Khan, F. Y., *et al.* 2010. (82) | Third/fourth/fifth generation cephalosporins or ESBLs | n/a | ESBL EC bacteremia | 27 | non-ESBL EC bacteremia | 70 | No selection, all EC bacteremia during the study period | Blood stream infection | Community-associated; Healthcare-associated; Nosocomial | in-hospital | 1) All-cause mortality |
| Kim, J., *et al.* 2014. (79) | Quinolones | All patients had cirrhosis | Fluoroquinolone-resistant EC spontaneous bacterial peritonitis | 26 | Fluoroquinolone-susceptible EC spontaneous bacterial peritonitis | 56 | No selection, all EC SBP during study period | Spontaneous bacterial peritonitis | Community-associated; Healthcare-associated | 30-day | 1) 30-day mortality, 2) All-cause mortality |
| Kim, S. H., *et al.* 2013. (73) | Third/fourth/fifth generation cephalosporins or ESBLs | All patients had neutropenic fever and had received either chemotherapy or stem cell transplant | ESBL EC bacteremia | 15 | non-ESBL EC bacteremia | 72 | No selection, all EC bacteremia in patients with neutropenic fever during the study period | Blood stream infection | Not reported | 30-day | 1) 30-day mortality, 2) All-cause mortality |
| Komatsu, Y., *et al.* 2018. (98) | Third/fourth/fifth generation cephalosporins or ESBLs | n/a | ESBL EC bacteremia | 30 | non-ESBL EC bacteremia | 85 | no selection | Blood stream infection | Community-associated; Healthcare-associated; Nosocomial | 14-days | 1) All-cause mortality |
| Lambert, M. L., *et al.* 2011. (53) | Third/fourth/fifth generation cephalosporins or ESBLs | All nosocomial (from ICU) and all cases from ICU | 3GC-R E. coli BSI | 42 | 3GC-S E. coli BSI | 218 | no selection | Blood stream infection | Nosocomial | during ICU stay | 1) All-cause mortality, 2) Post-infection length of stay |
| Laupland, K. B., *et al.* 2008. (105) | Quinolones | n/a | Ciprofloxacin-resistant EC BSI | 257 | Ciprofloxacin-susceptible EC BSI | 1947 | No selection | Blood stream infection | Community-associated; Healthcare-associated; Nosocomial | in-hospital | 1) All-cause mortality |
| Lee, H.,*et al.* 2018. (99) | Third/fourth/fifth generation cephalosporins or ESBLs | All emergency department admissions | ESBL EC UTI | 50 | non-ESBL EC UTI | 100 | Randomly enrolled 50 adult patients with ESBL EC UTI. Then 2:1 matching controls to cases , matched by sex and age (+/- 5 years) | Urinary tract infection | Community-associated; Healthcare-associated; Nosocomial | in-hospital | 1) All-cause mortality, 2) Length of stay |
| Lee, S., *et al.* 2014. (89) | Third/fourth/fifth generation cephalosporins or ESBLs | All treated with empirical ceftriaxone and had a urine culture performed o the first day of hospitalization (all community-onset) | ESBL EC acute pyelonephritis | 26 | non-ESBL EC acute pyelonephritis | 52 | Controls matched to cases 2:1 based on matching variables of bacteremia, age, sex, CCI, SAPS II and modified APN score. This was done using a 16 point scale. If there were more than 2 controls with the same score then two were selected randomly. | acute pyelonephritis | Community-associated; Healthcare-associated | 2 weeks | 1) All-cause mortality, 2) Treatment failure, 3) Length of stay |

| | | | | | | | | | | | |
|---|---|---|---|---|---|---|---|---|---|---|---|
| Leistner, R., *et al.* 2014. (112) | Third/fourth/fifth generation cephalosporins or ESBLs | n/a | ESBL EC BSI | 92 | non-ESBL EC BSI | 92 | Matching of cases to controls 1:1, matched on age +/- 5 yr, sex, CCI +/- 2, discharge year and the LOS before BSI onset in the control had to be at least as long as the case 80% of cases could be matched and 9.4% of controls used | Blood stream infection | Not reported | in-hospital | 1) Post-infection length of stay, 2) Healthcare system costs |
| Leistner, R., *et al.* 2014. (90) | Third/fourth/fifth generation cephalosporins or ESBLs | n/a | ESBL EC BSI | 115 | non-ESBL EC BSI | 983 | no selection | Blood stream infection | Community-associated; Healthcare-associated; Nosocomial | In-hospital | 1) All-cause mortality 2) Length of stay |
| Leistner, R., *et al.* 2014. (91) | Third/fourth/fifth generation cephalosporins or ESBLs | n/a | ESBL EC BSI | 178 | non-ESBL EC BSI | 1321 | No selection all EC BSI during study period | Blood stream infection | Community-associated; Healthcare-associated; Nosocomial | in-hospital | 1) All-cause mortality |
| Lim, C., *et al.* 2016. (45) | Multidrug resistance | n/a | MDR EC BSI (MDR based on Magiorakos 2012, non-susceptible to at least three or more antimicrobial categories | 1717 | non-MDR EC BSI | 2562 | no selection | Blood stream infection | Community-associated; Healthcare-associated; Nosocomial | 30-day | 1) 30-day mortality, 2) All-cause mortality |
| Ma, J., *et al.* 2017. (48) | Third/fourth/fifth generation cephalosporins or ESBLs | All had hematologic disease, all nosocomial | ESBL E. coli bacteremia | 70 | non-ESBL E. coli bacteremia | 43 | No selection, all nosocomial E. coli bacteremias during study period<br>Had to be hematologic patients at least 14 years old | Blood stream infection | Nosocomial | 30 days | 1) 30-day mortality, 2) All-cause mortality |
| Martelius, T., *et al.* 2016. (56) | Third/fourth/fifth generation cephalosporins or ESBLs | n/a | EC BSI isolates that were resistant or intermediate to third-generation cephalosporins | 182 | EC BSI isolates that were susceptible to third-generation cephalosporins | 2035 | No selection, all the EC BSI during the study period | Blood stream infection | Nosocomial | 28 day | 1) 30-day mortality, 2) All-cause mortality |
| Maslikowska, J. A., *et al.* 2016. (93) | Third/fourth/fifth generation cephalosporins or ESBLs | n/a | ESBL-EC infection | 61 | non-ESBL-EC infection | 49 | Cases consecutively enrolled in reverse chronological order of hospital admission date. Controls matched to cases 1:1, on the basis of sex, age +/- 5 yr, type of infection, and bed allocation | Blood stream infection; Urinary tract infection; intra-abdominal; pneumonia; joint | Not reported | 14 days following discontinuation of antibiotics | 1) All-cause mortality, 2) Bacterium-attributable mortality, 3) Treatment failure, |

| | | | | | | | | | | | |
|---|---|---|---|---|---|---|---|---|---|---|---|
| Melzer, M., *et al.* 2007. (65) | Third/fourth/fifth generation cephalosporins or ESBLs | n/a | ESBL EC bacteremia | 46 | non-ESBL EC bacteremia | 308 | No selection all EC bacteremia during study period | Blood stream infection | Community-associated; Nosocomial No definitions of CA and Nos, not sure if HCa are included in CA | 30-days | 1) 30-day mortality, 2) All-cause mortality, 3) Post-infection length of hospital stay |
| Namikawa, H., *et al.* 2017. (96) | Third/fourth/fifth generation cephalosporins or ESBLs | n/a | ESBL EC bacteremia | 31 | non-ESBL EC bacteremia | 98 | No selection all EC bacteremia during the study period | Blood stream infection | Nosocomial, What the non-nosocomial represents is not reported | not reported | 1) All-cause mortality |
| Nicolas-Chanoine, M. H., *et al.* 2012. (86) | Third/fourth/fifth generation cephalosporins or ESBLs | | CTX-M producing ESBL EC | 152 | non-ESBL EC | 152 | The first inpatient with a clinical sample positive for a non-ESBL EC the same day or within 3 days of the case | All types of EC infections | Community-associated; Healthcare-associated; Nosocomial | in-hospital | 1) All-cause mortality |
| Nussbaum, A., et al. 2018. (88) | Third/fourth/fifth generation cephalosporins or ESBLs | n/a | ESBL E. coli BSI | 34 | non-ESBL E. coli BSI | 66 | Cases that met eligibility criteria during the study period and controls matched by age and gender | Blood stream infection | Community-associated; Healthcare-associated | in hospital | 1) All-cause mortality, 2) Length of hosptial stay |
| Ortega, M., *et al.* 2009. (58) | Third/fourth/fifth generation cephalosporins or ESBLs | n/a | ESBL EC bacteremia | 211 | non-ESBL EC bacteremia | 4547 | No selection, all EC bacteremia during study period | Blood stream infection | Community-associated; Healthcare-associated; Nosocomial | From diagnosis of bacteremia until 30-days afterwards, until death or until discharge | 1) 30-day mortality, 2) All-cause mortality |
| Ortega, M., *et al.* 2009. (58) | Quinolones | n/a | Fluoroquinolone resistant EC bacteremia | 1300 | Fluoroquinolone susceptible EC bacteremia | 3458 | No selection, all EC bacteremias during study period | Blood stream infection | Community-associated; Healthcare-associated; Nosocomial | Diagnosis of bacteremia until 30 days afterward, death or discharge from hospital | 1) 30-day mortality, 2) All-cause mortality |

| | | | | | | | | | | | |
|---|---|---|---|---|---|---|---|---|---|---|---|
| Park, S. H., *et al.* 2011. (69) | Third/fourth/fifth generation cephalosporins or ESBLs | All community-onset (CA and HCa) | ESBL EC bacteremia | 50 | non-ESBL EC bacteremia | 100 | All cases during study period and controls were selected by computer generated random number sampling using the culture register numbers in the microbiology laboratory of the hospital. The controls were selected during the same or following month as the corresponding case. Two controls per case. | Blood stream infection | Community-associated; Healthcare-associated | 30 days | 1) 30-day mortality, 2) All-cause mortality |
| Park, S. H., *et al.* 2015. (94) | Third/fourth/fifth generation cephalosporins or ESBLs | All community-associated | ESBL EC acute pyelonephritis | 75 | non-ESBL EC acute pyelonephritis | 225 | All cases during the study period, for each case, three controls were randomly selected from each risk set composed of all eligible control patients treated within a month of the time each case was identified | acute pyelonephritis | Community-associated | 4 weeks after the completion of antibiotic therapy | 1) All-cause mortality, 2) Treatment failure, 3) Length of hospital stay |
| Parveen, A., *et al.* 2015. (39) | Multidrug resistance | All cancer patients | MDR EC bacteremia, MDR defined as resistant to three or more classes of antimicrobial agents, including fluoroquinolones, third-generation cephalosorins, antipseudomonal penicillins + beta-lactamase inhibitors and carbapenems | 98 | non-MDR EC bacteremia | 129 | No selection, all EC bactermias during study period | Blood stream infection | Not reported | 30-day | 1) 30-day mortality, 2) All-cause mortality |
| Pena, C., *et al.* 2008. (66) | Third/fourth/fifth generation cephalosporins or ESBLs | n/a | ESBL EC infection | 100 | non-ESBL EC infection | 100 | All consecutive cases during the study period, and controls were matched 1:1 according to the same site of infection and closest date of admission | All types of EC infections | Not reported | 30-days | 1) 30-day mortality, 2) All-cause mortality |
| Peralta, G., *et al.* 2007. (57) | Third/fourth/fifth generation cephalosporins or ESBLs | n/a | EC bacteremia resistant to cefotaxime | 31 | EC bacteremia susceptible to cefotaxime | 632 | No selection all EC bacteremia during study period | Blood stream infection | Nosocomial only type mentioned, no details on the other 90.6% | in-hospital | 1) All-cause mortality |

| | | | | | | | | | | | |
|---|---|---|---|---|---|---|---|---|---|---|---|
| Peralta, G., *et al.* 2007. (57) | Quinolones | n/a | EC bacteremia resistant to ciprofloxacin | 125 | EC bacteremia susceptible to ciprofloxacin | 538 | No selection, all EC bacteremias during study period enrolled | Blood stream infection | Nosocomial only type mentioned, no details on the other 90.6% | in hospital | 1) All-cause mortality |
| Riu, M., *et al.* 2016. (55) | Multidrug resistance | All nosocomial | MDR EC bacteremia, MDR based on Magiorakos criteria (three or more antimicrobial categories) | 39 | non-MDR EC bacteremia | 145 | All nosocomial EC bacteremias during study period | Blood stream infection | Nosocomial | in hospital | 1) Healthcare system costs |
| Rodriguez-Bano, J. *et al.* 2010. (83) | Third/fourth/fifth generation cephalosporins or ESBLs | All community-onset | ESBL EC bacteremia | 95 | non-ESBL EC bacteremia | 188 | All cases during the study period, 2 controls per case were chosen from non-ESBL EC bacteremias diagnosed during the month following the corresponding case and had to be from the same hospital (randomly selected using a computerized method) | Blood stream infection | All community-onset - Community-associated; Healthcare-assoicated | 14 day, patients followed until death or discharge | 1) All-cause mortality |
| Shin, J., *et al.* 2012. (106) | Quinolones | All uncomplicated acute pyelonephritis all treated initially with ciprofoxacin or levofloxacin | CIP-resistant E coli UAPN | 32 | CIP-susceptible E coli UAPN | 173 | All UAPN E coli patients during time period | Uncomplicated acute pyelonephritis | Community-associated; Healthcare-associated | In hospital, follow-up visits if available | 1) All-cause mortality, 2) Treatment failure, 3) Length of hospital stay |
| Soraas, A., *et al.* 2014. (109) | Third/fourth/fifth generation cephalosporins or ESBLs | n/a | ESBL-producing E. coli UTI | 81 | non-ESBL-producing E. coli UTI | 262 | All cases in the time period, 2-5 controls were randomly selected using excel randomization everytime a case was identified. Both cases and controls had to meet eligibility criteria and provide consent. | Urinary tract infection | Community-associated | 2 weeks after initial prescription | 1) Treatment failure |
| Thaden, J. T., *et al.* 2017. (63) | Multidrug resistance | n/a | monomicrobial gram-negative BSI, MDR - Magiorakos, resistance to at least one antimicrobial in at least three categories | 165 | monomicrobial gram-negative BSI, non-MDR - resistance to at least one antimicrobial in less than three categories or pansusceptibility | 165 | No selection, all cases and controls since 01/2009 that provided consent | Blood stream infection | not reported | in-hospital | 1) Healthcare system costs |

| | | | | | | | | | | | |
|---|---|---|---|---|---|---|---|---|---|---|---|
| Trecarichi, E. M., *et al.* 2009. (59) | Third/fourth/fifth generation cephalosporins or ESBLs | All cases and controls have hematological malignancy | ESBL E. coli BSI | 26 | non-ESBL E. coli BSI | 36 | No selection,, all cases and controls in the time period | Blood stream infection | Community-associated; Healthcare-associated; Nosocomial | 30 days | 1) 30-day mortality, 2) All-cause mortality |
| Trecarichi, E. M., *et al.* 2009. (59) | Quinolones | Hematological malignancy in all cases and controls | Fluoroquinolone R E coli BSI | 39 | Fluoroquinolone S E coli BSI | 23 | No selection all cases and controls in study time period included | Blood stream infection | Community-associated; Healthcare-associated; Nosocomial | 30 days | 1) 30-day mortality, 2) All-cause mortality |
| Tumbarello, M., *et al.* 2010. (84) | Third/fourth/fifth generation cephalosporins or ESBLs | n/a | ESBL-producing E. coli BSI | 37 | non-ESBL-producing E. coli BSI | 97 | No selection, all cases and controls included from time period | Blood stream infection | Community-associated; Healthcare-associated; Nosocomial | In hospital, 21-day | 1) All-cause mortality, 2) Treatment failure, 3) Post-infection length of hospital stay, 4) Healthcare system costs |
| Uzodi, A. S., *et al.* 2017. (62) | Multidrug resistance | n/a | MDR EC infection is resistant to any antibiotic in 3 or more of the following drug classes : 1) ampicillin/ sulbactam, 2) piperacillin/ tazobactam, 3) TMS, 4) fluoroquinolones, 5) aminoglycosides, 6) 1st ir 2nd generation cephalosporins, 7) extended-spectrum cephalosporins, 8) carbapenems | 34 | non-MDR EC infection | 284 | No selection all cases and controls during the study period | Not reported, 90% from urine but that includes colonization | Not reported for infections only, including colonization Community-associated; Healthcare associated; Nosocomial | not reported | 1) All-cause mortality, 2) Treatment failure, 3) Length of hospital stay |
| Van Aken, S., *et al.* 2014. (92) | Third/fourth/fifth generation cephalosporins or ESBLs | n/a | ESBL-producing E. coli BSI | 70 | non-ESBL-producing E. coli BSI | 140 | The cases were enrolled if they met inclusion criteria and provided consent during the study period. The controls were matched for time period, bacterial species, occurrence of bacteremia, and study location. Controls selected by computer randomization of all possible controls. Cases to controls 1:2. | Blood stream infection | Community-associated; Healthcare-associated; Nosocomial | 14 days | 1) All-cause mortality, 2) Length of hospital stay |
| Yip, T., *et al.* 2006. (81) | Third/fourth/fifth generation cephalosporins or ESBLs | All end-stage renal disease treated by continuous ambulatory peritoneal dialysis | ESBL-producing E. coli | 11 | non-ESBL-producing E. coli | 77 | All  E. coli peritonitis cases in the study time period, then cases ESBL and controls non-ESBL | Peritonitis | Not reported | not reported | 1) All-cause mortality, 2) Treatment failure |

| | | | | | | | | | | | |
|---|---|---|---|---|---|---|---|---|---|---|---|
| Yoon, E. J., *et al.* 2018. (61) | Third/fourth/fifth generation cephalosporins or ESBLs | n/a | resistant to 3rd and 4th generation cephalosporins in E. coli BSI | 524 | susceptible to 3rd and 4th generation cephalosporins in E. coli BSI | 968 | No selection,, all E. coli BSI during study periods | Blood stream infection | Not reported | Until hospital discharge or at least for 30 days | 1) 30-day mortality, 2) All-cause mortality |
| Yoon, E. J., *et al.* 2018. (61) | Quinolones | n/a | E. coli BSI resistant to fluoroquinolones | 590 | E. coli BSI susceptible to fluoroquinolones | 902 | No selection, all E. coli BSI during the study period | Blood stream infection | Not reported | Until hospital discharge or at least for 30 days | 1) 30-day mortality, 2) All-cause mortality |
| Yoon, E. J., *et al.* 2018. (61) | Multidrug resistance | n/a | MDR, nonsusceptible to at least 3 drug classes , ref Magiorakos | 865 | non-MDR, nonsusceptible to less than 3 drug classes | 627 | No selection, all E. coli BSI during study period | Blood stream infection | Not reported | Until hospital discharge or at least 30 days | 1) 30-day mortality, 2) All-cause mortality |
