# Supplementary material for: Evaluation of the health and healthcare system burden due to antimicrobial-resistant Escherichia coli infections in humans: a systematic review and meta-analysis
Source: Antimicrob Resist Infect Control. 2020 Dec 10;9:200. doi: 10.1186/s13756-020-00863-x (PMC7726913; doi:10.1186/s13756-020-00863-x)

Additional file 7 - Summaries of risk of bias for studies assessing specified burden of disease measure and type of antimicrobial resistance in *E. coli* infections.

Summary of risk of bias for studies assessing 30-day mortality and third-generation cephalosporin resistance (n=23 studies).

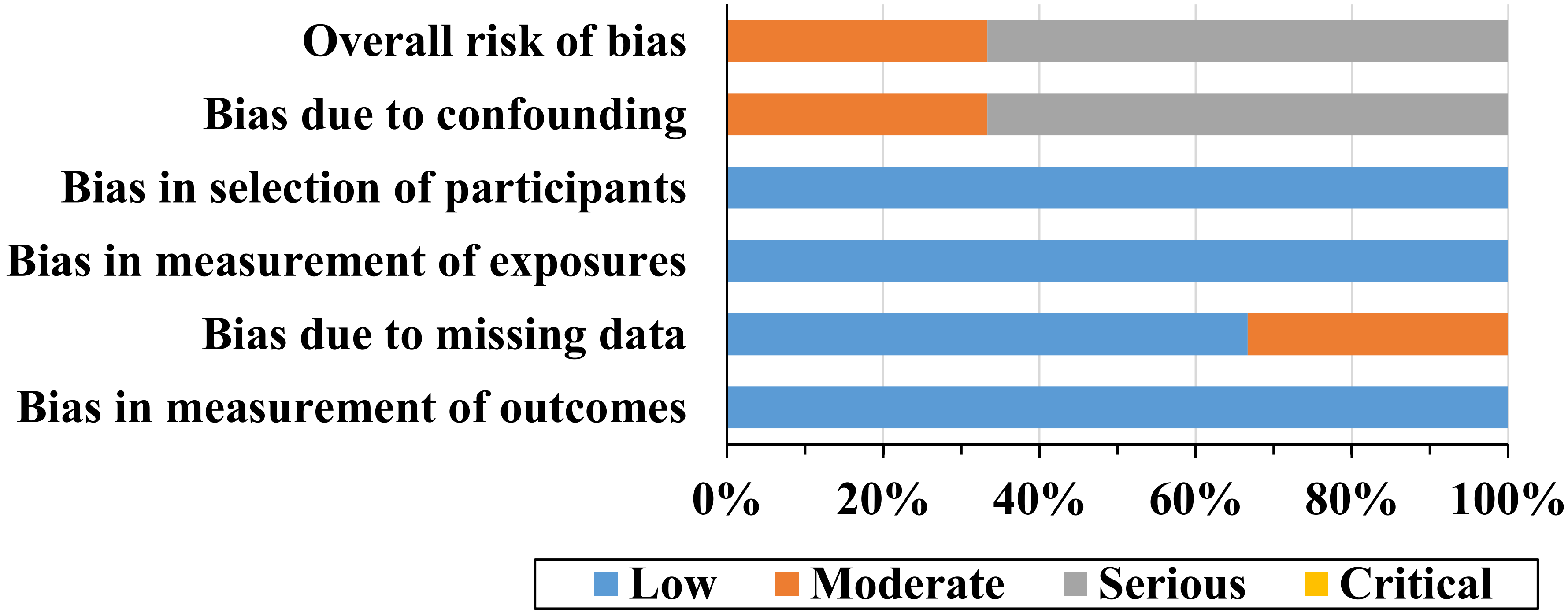

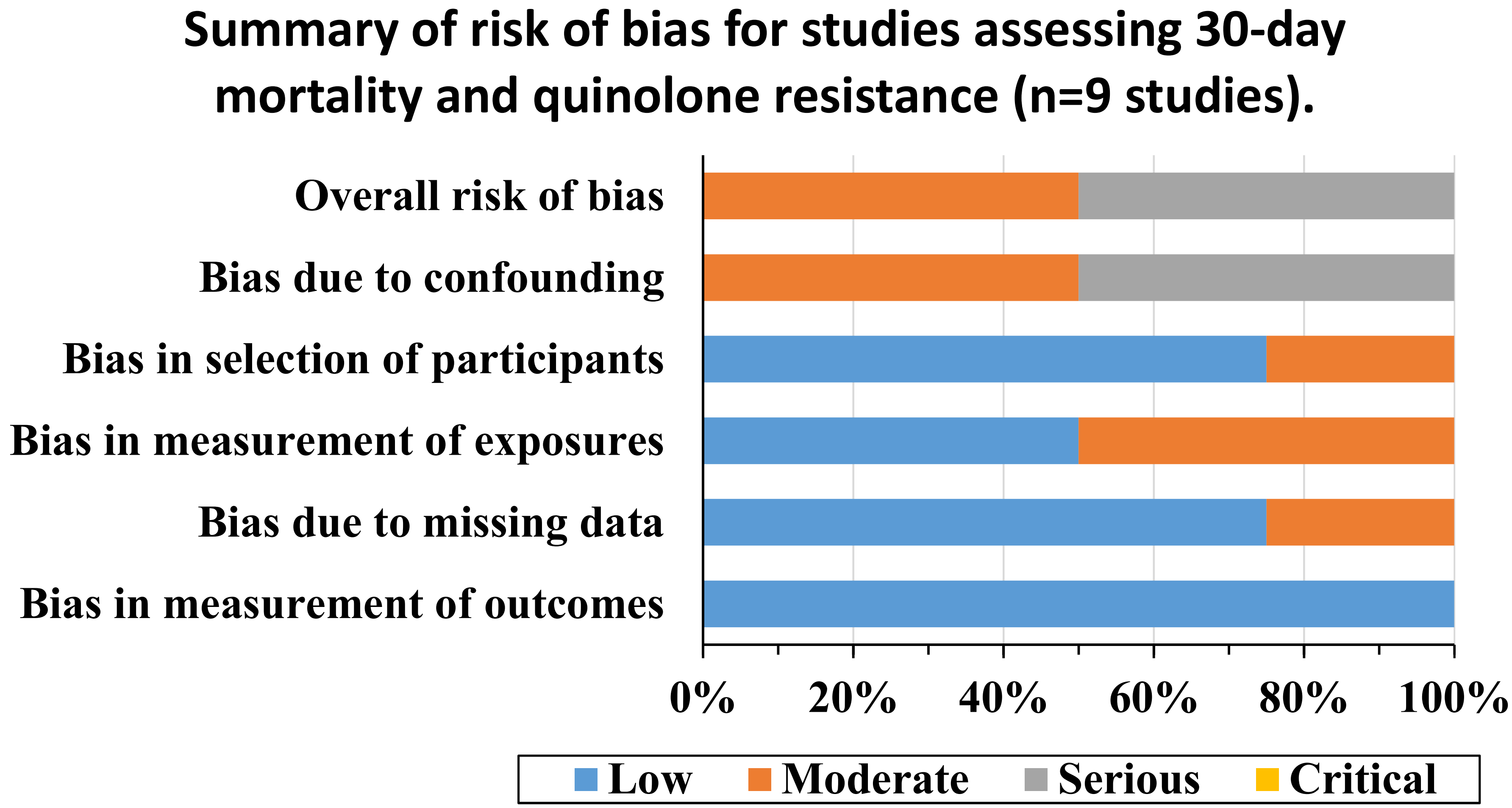
Summary of risk of bias for studies assessing 30-day mortality and quinolone resistance (n=9 studies).
Overall risk of bias
Bias due to confounding
Bias in selection of participants
Bias in measurement of exposures
Bias due to missing data
Bias in measurement of outcomes
0%
20%
40%
60%
80%
100%
Low
Moderate
Serious
Critical

Summary of risk of bias for studies assessing 30-day mortality and MDR (n=4 studies).

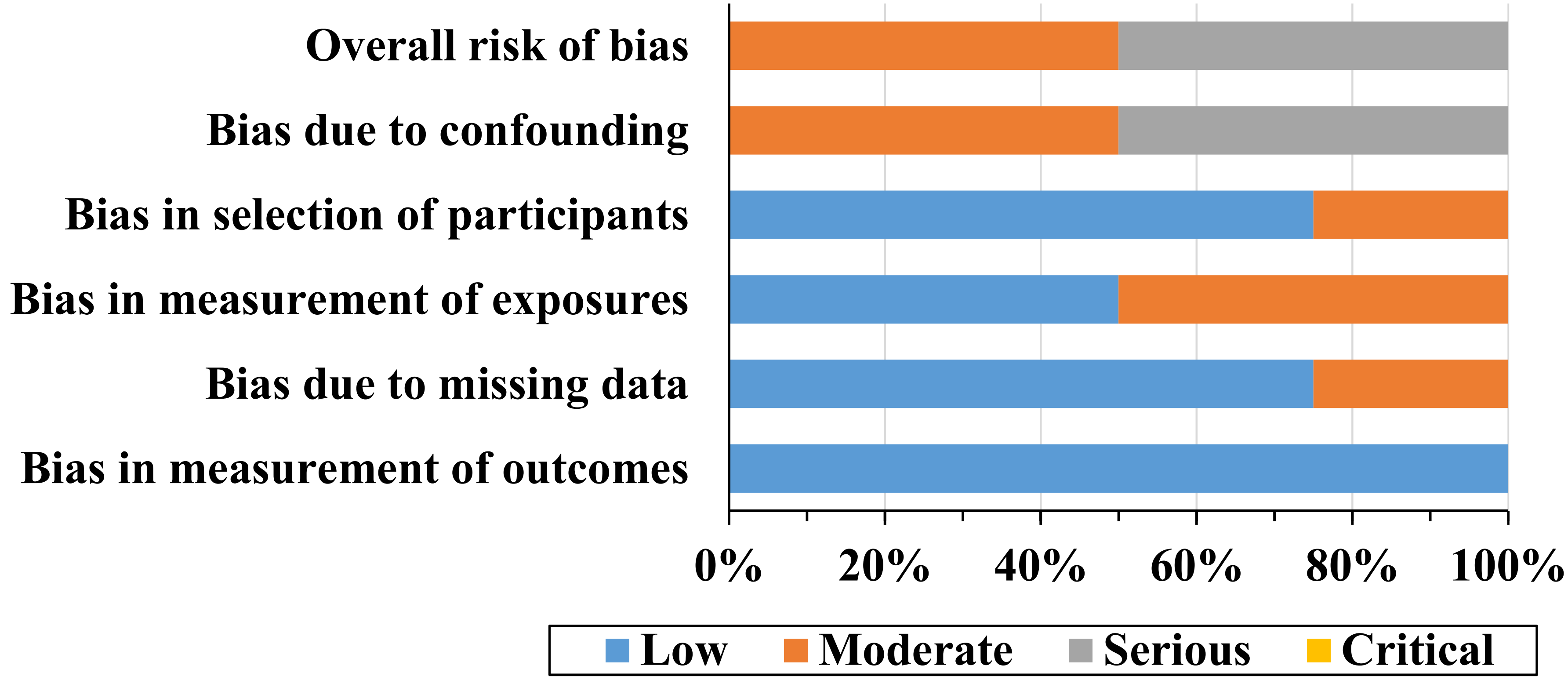

Summary of risk of bias for studies assessing all-cause mortality and third-generation cephalosporin resistance (n=51 studies).

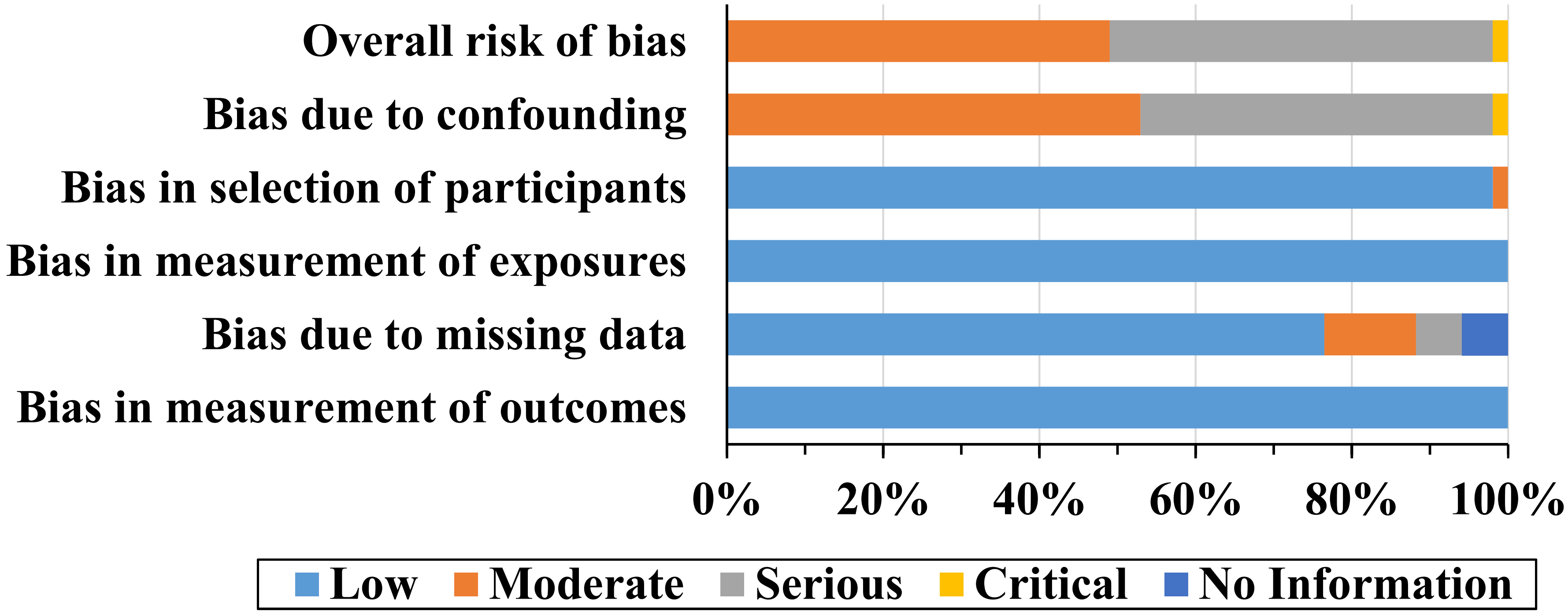

Summary of risk of bias for studies assessing all-cause mortality and quinolone resistance (n=17 studies).

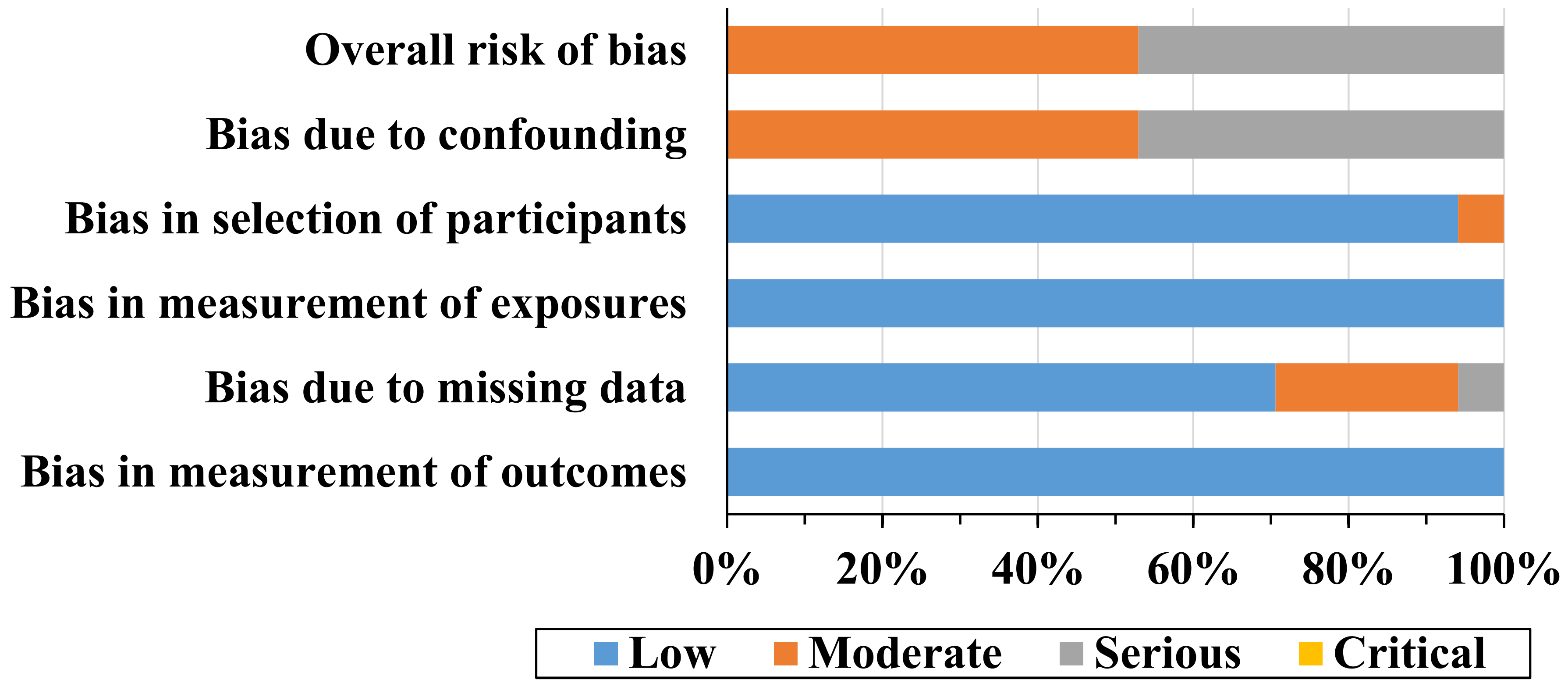

Summary of risk of bias for studies assessing all-cause mortality and MDR (n=5 studies).

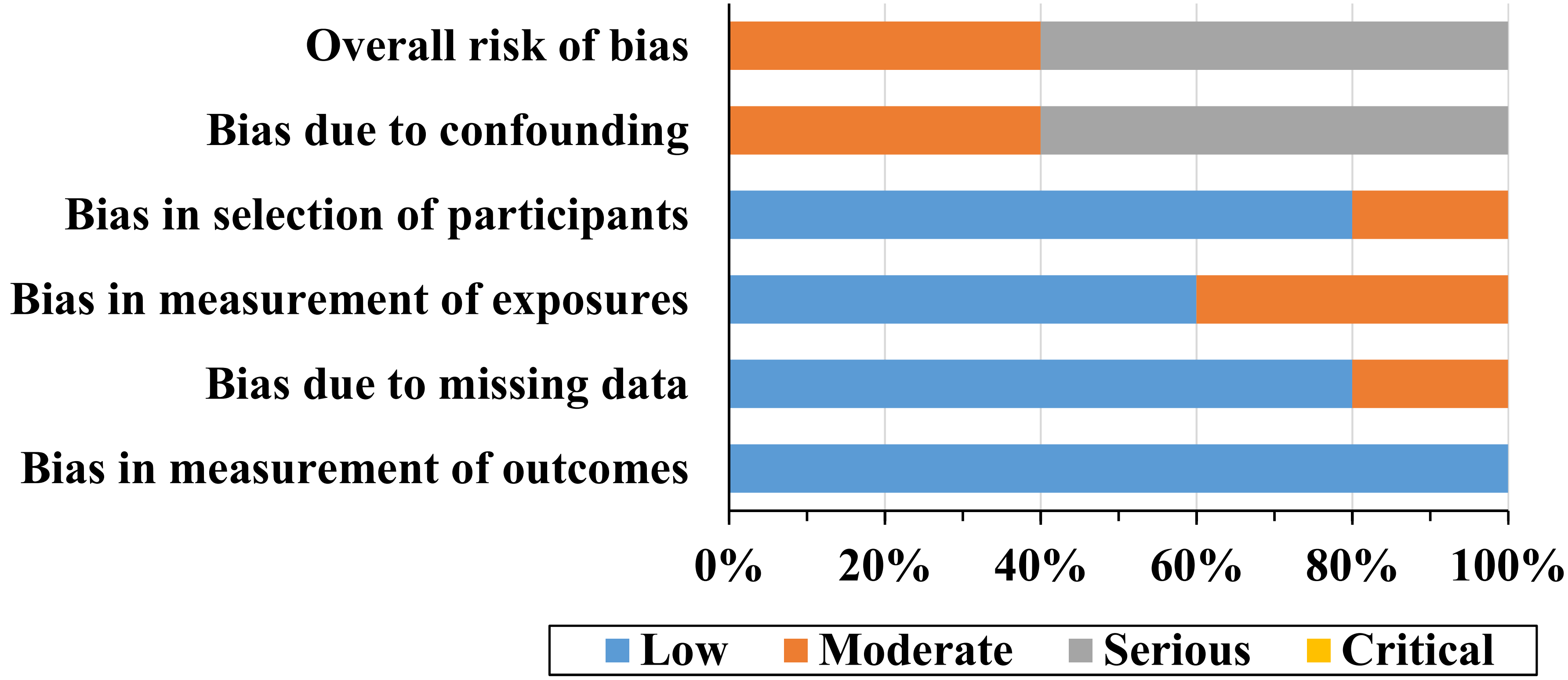

Summary of risk of bias for studies assessing bacterium-attributable mortality and third-generation cephalosporin resistance (n=3 studies).

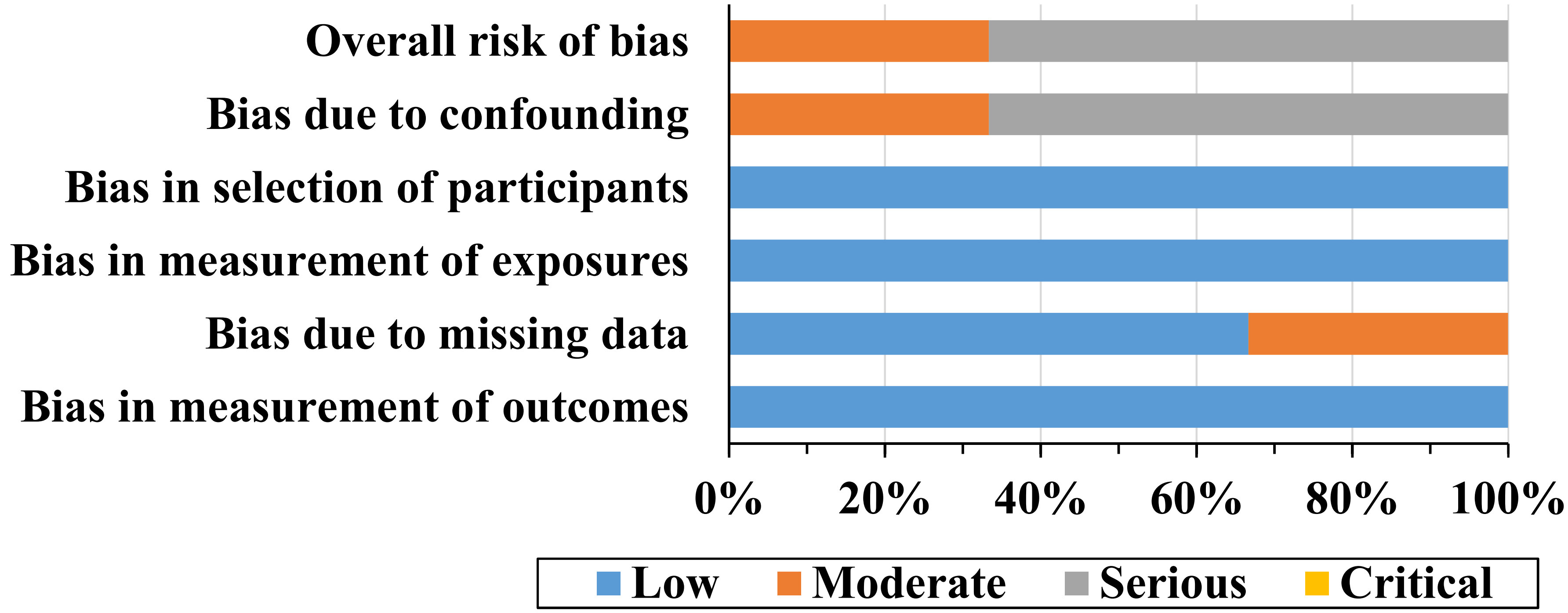

Summary of risk of bias for studies assessing treatment failure and third-generation cephalosporin resistance (n=15 studies).

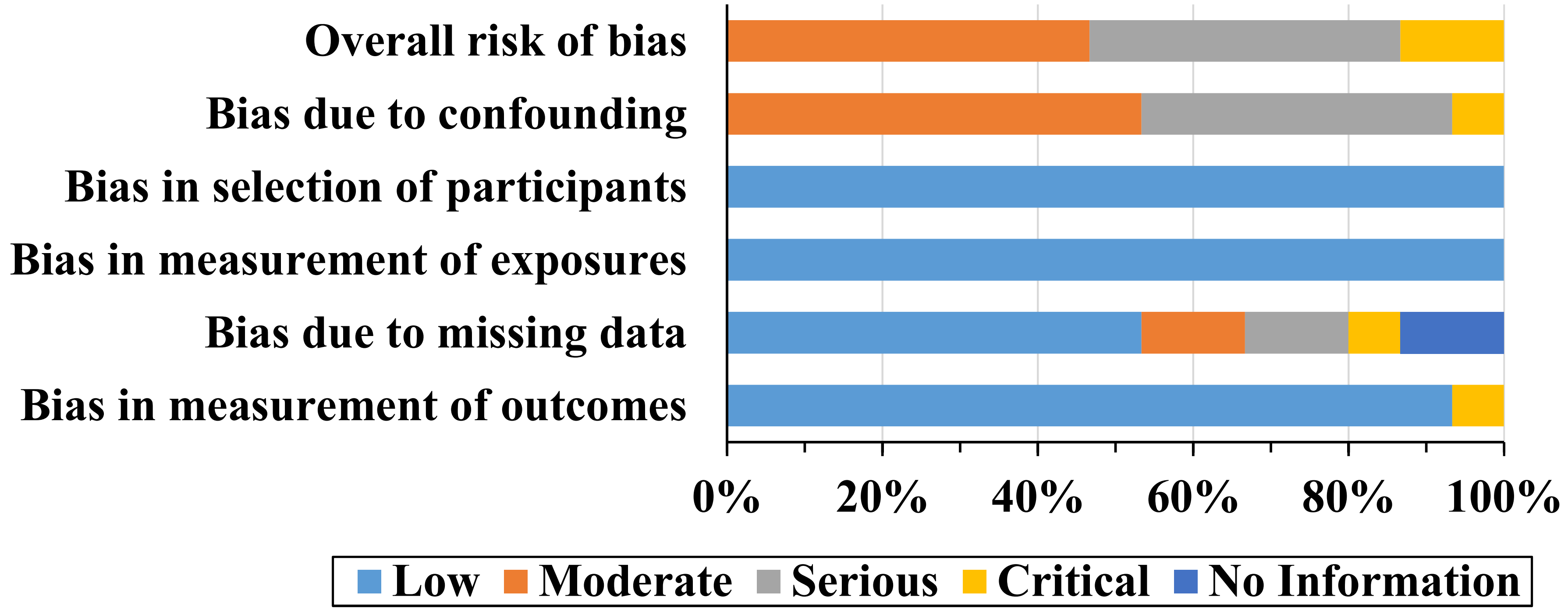

Summary of risk of bias for studies assessing treatment failure and quinolone resistance (n=7 studies).

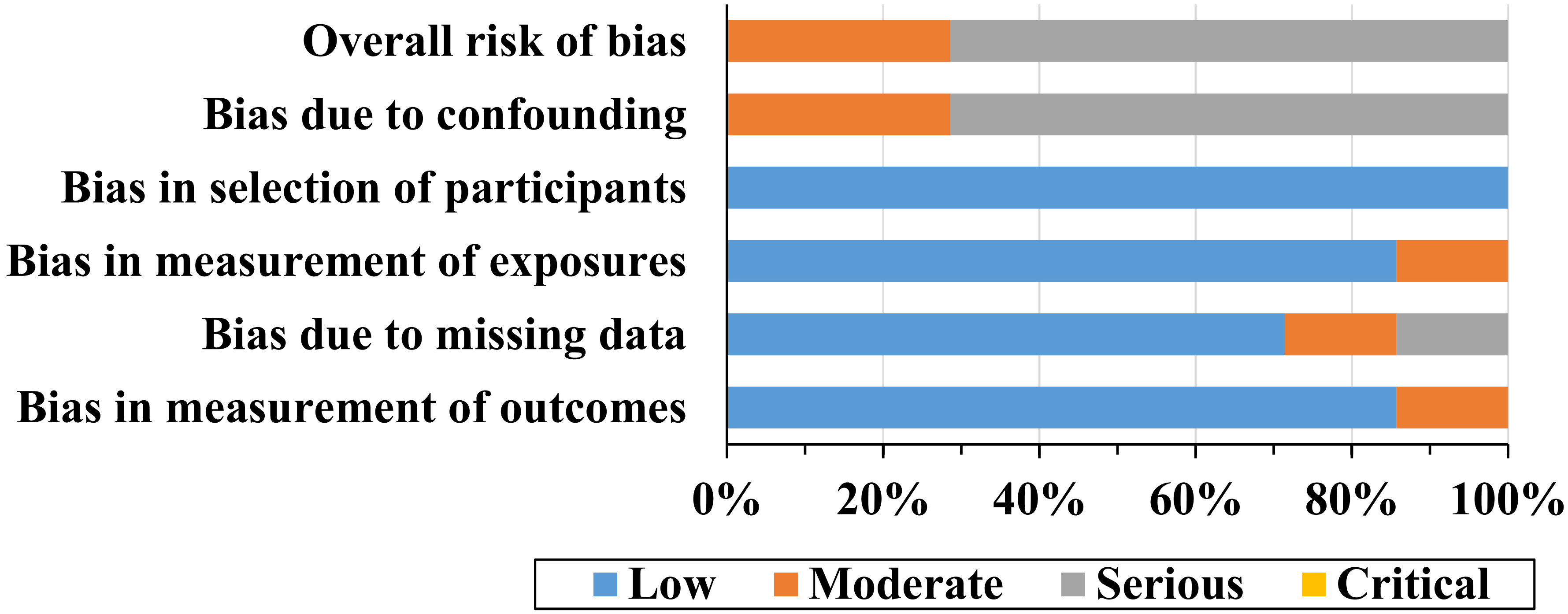

Summary of risk of bias for studies assessing treatment failure and MDR (n=2 studies).

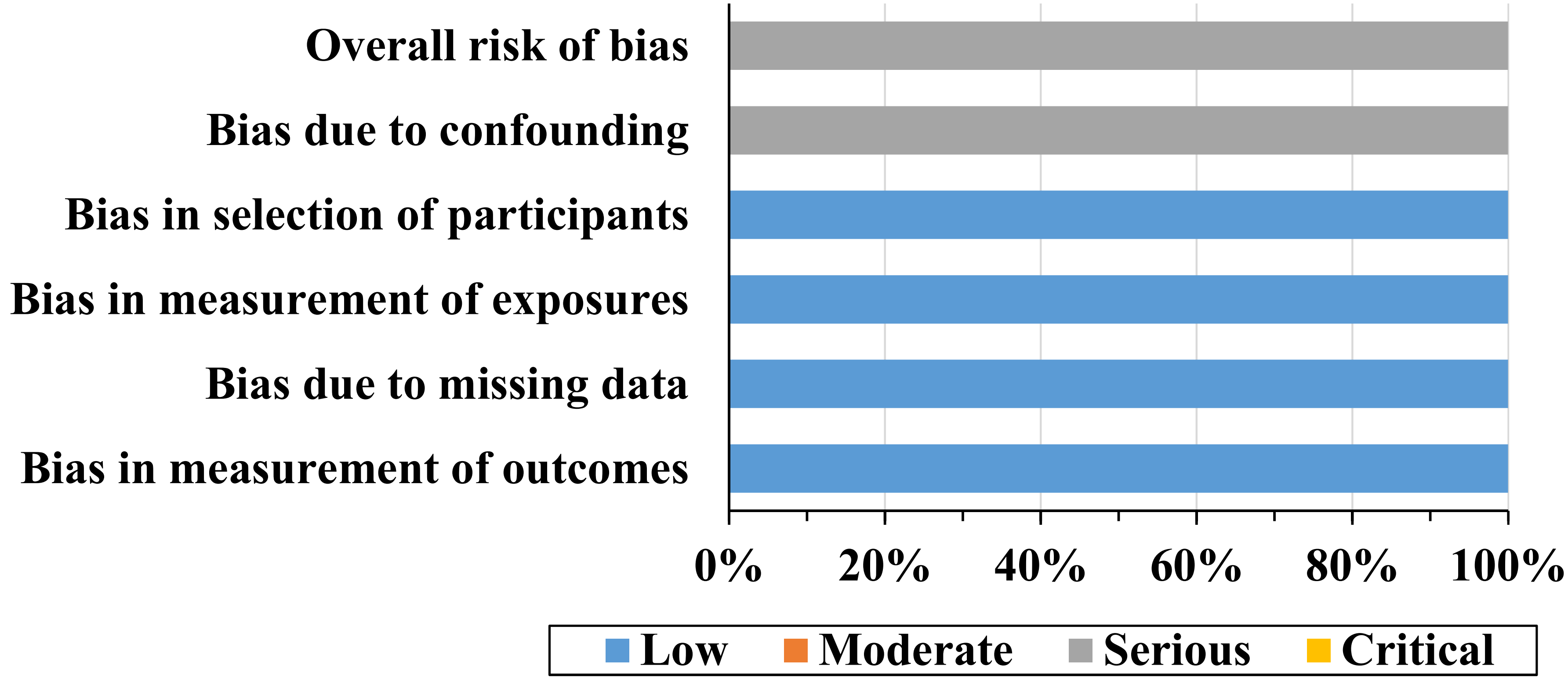

Summary of risk of bias for studies assessing total length of hospital stay and third-generation cephalosporin resistance (n=13 studies).

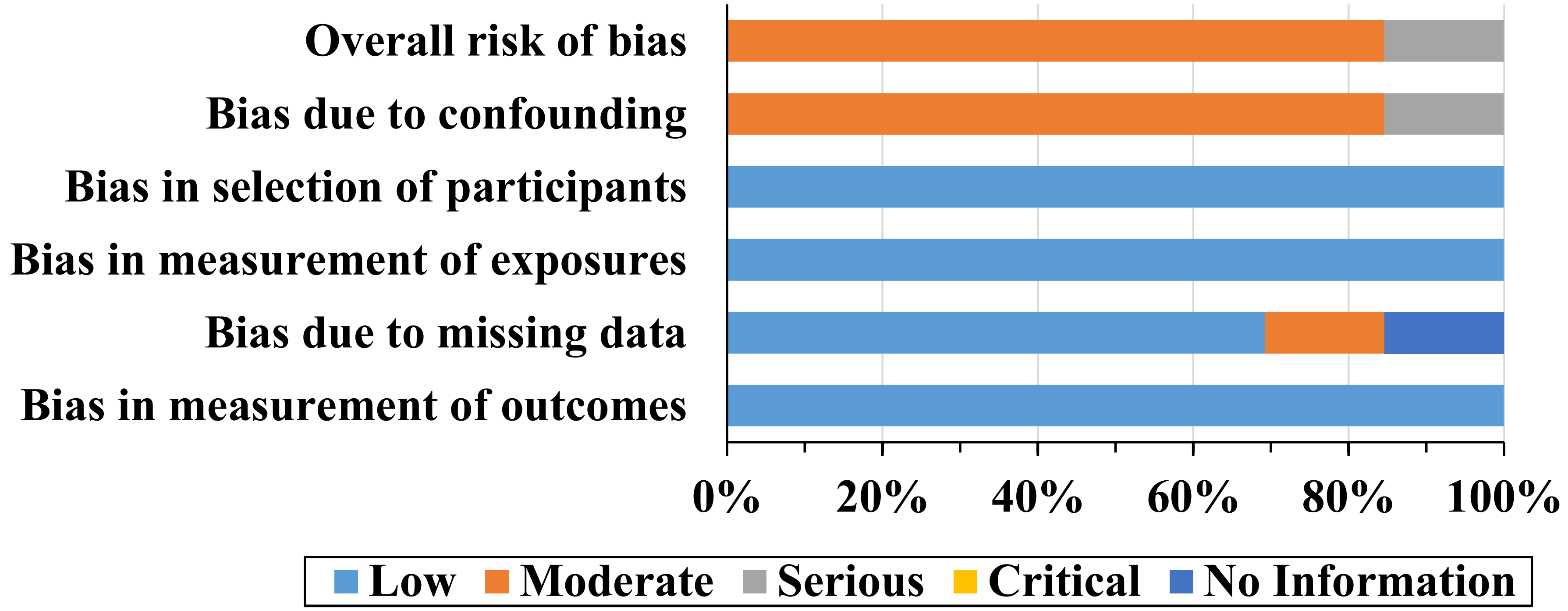

Summary of risk of bias for studies assessing total length of hospital stay and quinolone resistance (n=4 studies).

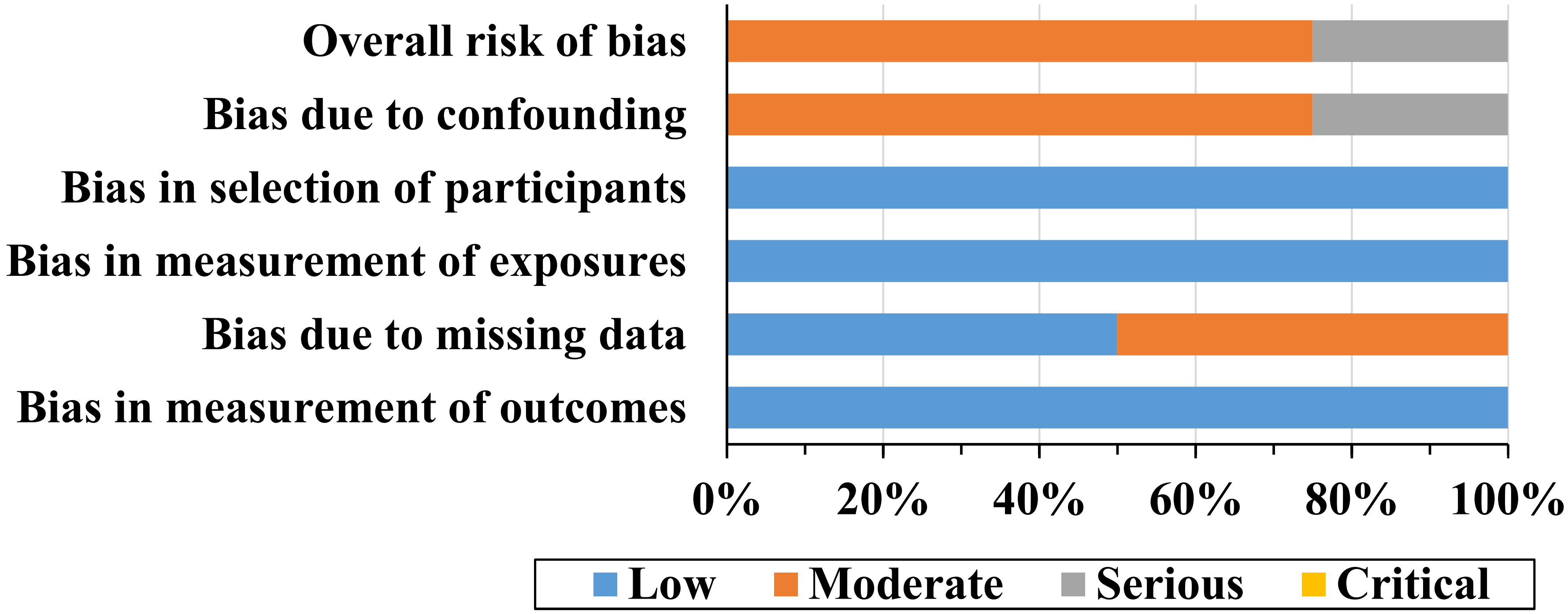

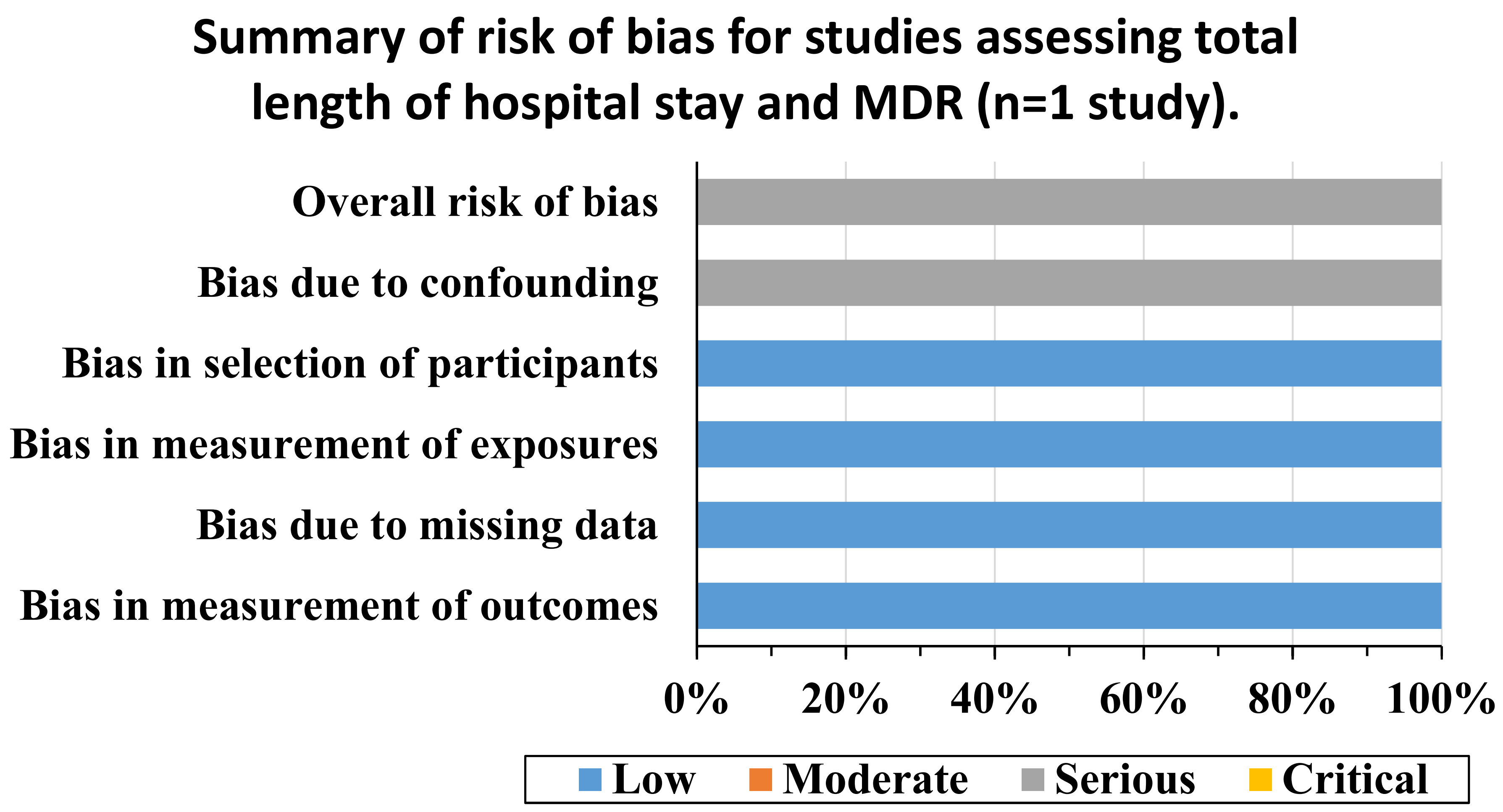

Summary of risk of bias for studies assessing total length of hospital stay and MDR (n=1 study).
Overall risk of bias
Bias due to confounding
Bias in selection of participants
Bias in measurement of exposures
Bias due to missing data
Bias in measurement of outcomes
0%
20%
40%
60%
80%
100%
Low
Moderate
Serious
Critical

Summary of risk of bias for studies assessing post-infection length of hospital stay and third-generation cephalosporin resistance (n=7 studies).

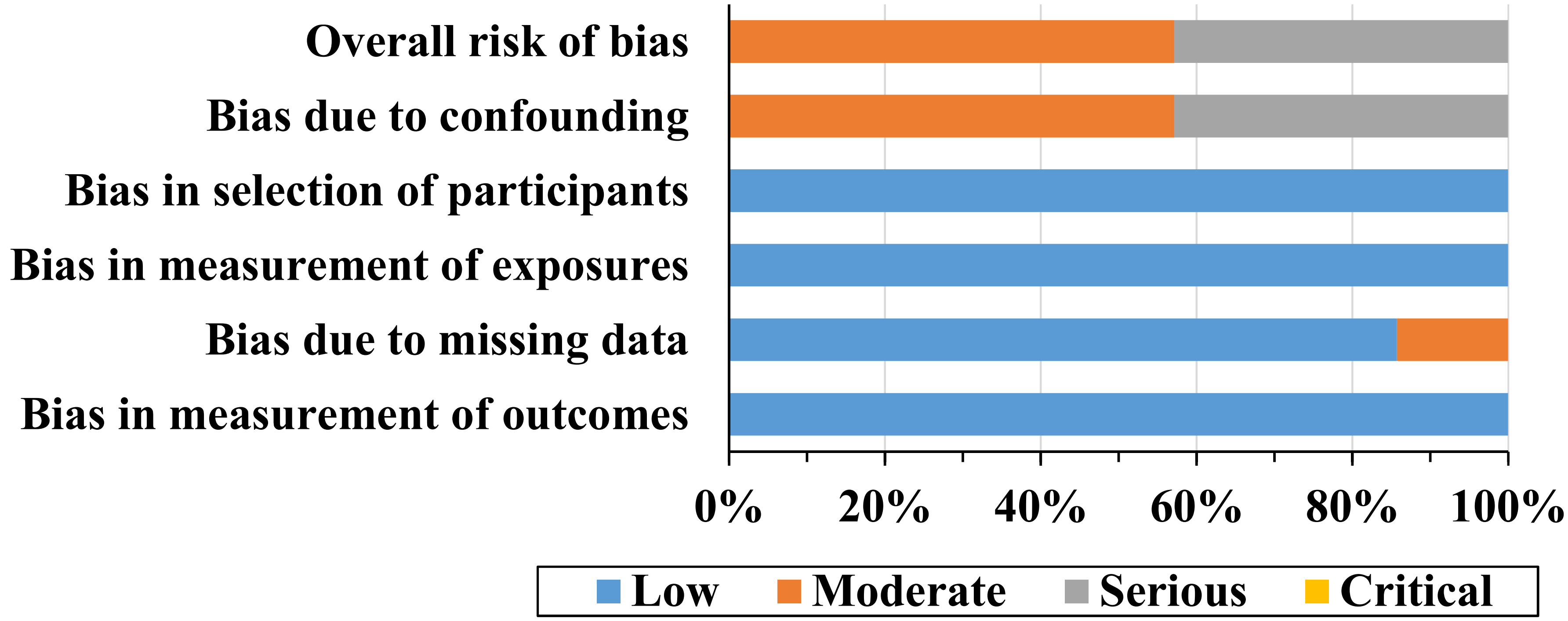

Summary of risk of bias for studies assessing healthcare costs and third-generation cephalosporin resistance (n=4 studies).

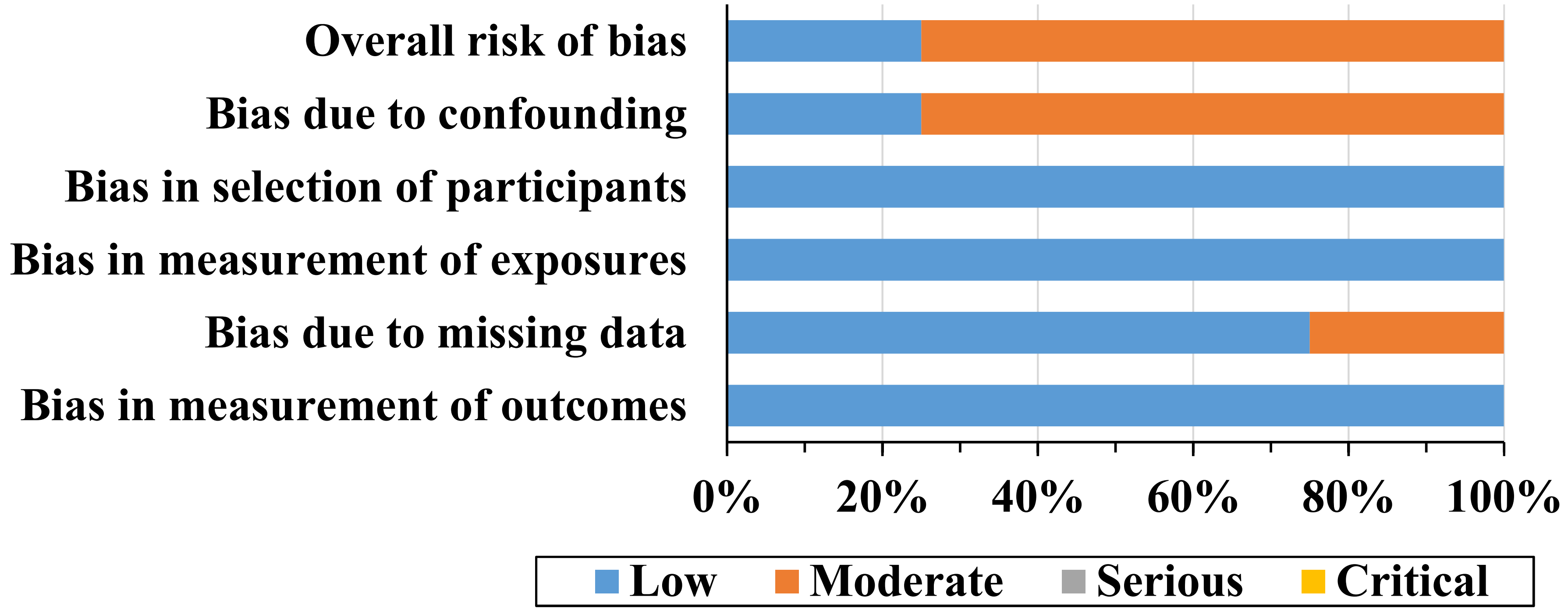

Summary of risk of bias for studies assessing healthcare costs and MDR (n=2 studies).

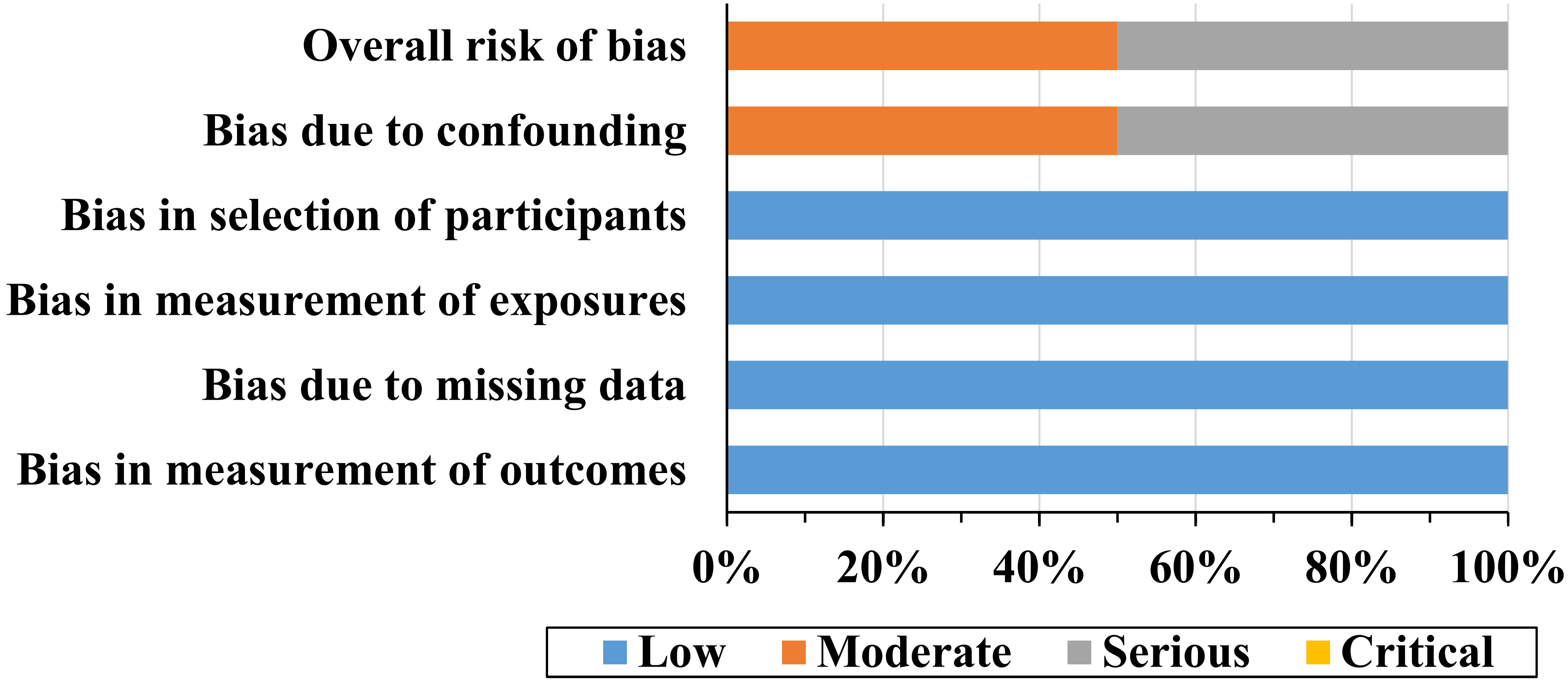

Supplement: Supplementary file 7 — Additional file 7: Summaries of risk of bias for studies assessing specified burden of disease measure and type of antimicrobial resistance [file 13756_2020_863_MOESM7_ESM.pdf]
