# Supplementary material for: Evaluation of the health and healthcare system burden due to antimicrobial-resistant Escherichia coli infections in humans: a systematic review and meta-analysis
Source: Antimicrob Resist Infect Control. 2020 Dec 10;9:200. doi: 10.1186/s13756-020-00863-x (PMC7726913; doi:10.1186/s13756-020-00863-x)
Supplement: Supplementary file 8 — Additional file 8: Results for 30-day mortality and the type of antimicrobial resistance of interest for the systematic review [file 13756_2020_863_MOESM8_ESM.pdf]

Additional file 8 - Results for 30-day mortality and the type of resistance of interest for a systematic review evaluating whether the measures of health or healthcare system burden increase in humans with antimicrobial-resistant *E. coli* infections.

Citation (Reference # in manuscript)	Deaths in resistant (R) group	Total in R	Deaths in susceptible (S) group	Total in S	Odds ratio (OR)	95% Cofidence interval	OR calculated from raw data or extracted from study	Crude or adjusted OR	Details of multivariable logistic regression	Details of matching	β (Coefficient)	Standard error	Comments
**8a) Third-generation cephalosporin resistance**													
Abernethy JK, 2015 (50)	392	1838	2911	16641	**1.28**	**1.14-1.44**	Extracted	Crude	n/a	n/a	0.247	0.060	
Cheong HS, 2007 (60)	5	19	64	489	2.37	0.86-6.57	Calculated	Crude	n/a	n/a	0.863	0.519	
de Kraker ME, 2011 (49)	34	105	180	1067	**2.36**	**1.53-3.65**	Calculated	Crude	n/a	n/a	0.859	0.222	
Denis B, 2015 (74)	12	41	11	41	1.23	0.36-4.23	Extracted	Adjusted	Adjusted for APACHE II score (severity)	Matched on date of diagnosis	0.207	0.629	
Esteve-Palau E, 2015 (75)	6	60	4	60	1.56	0.44-5.43	Calculated	Crude	n/a	Matched on age, sex, and date of admission	0.445	0.641	
Freeman JT, 2012 (70)	10	16	2	16	**11.67**	**2.1-61.93**	Calculated	Crude	n/a	Matched on species of organism, type of blood culture, timing of bacteremia, and data of diagnosis	2.457	0.863	
Gudiol C, 2010 (67)	6	17	23	118	2.25	0.78-6.54	Calculated	Crude	n/a	n/a	0.811	0.542	Results are for episodes not patients.
Ha YE, 2013 (72)	21	95	31	255	**3.01**	**1.45-6.28**	Extracted	Adjusted	Adjusted for septic shock, mechanical ventilation, source from UTI, source from intra-abdominal, source from respiratory, and CCI	n/a	1.102	0.374	
Haruki Y, 2018 (76)	9	24	12	77	**3.25**	**1.19-8.96**	Extracted	Crude	n/a	n/a	1.179	0.515	
Ho PL, 2002 (64)	9	50	7	100	**2.92**	**1.05-8.1**	Calculated	Crude	n/a	Matched on specialty, age (+/- 10 yr), sex, and date of isolation	1.072	0.521	Results are for episodes not patients.
Hsieh CJ, 2010 (68)	4	19	42	385	2.18	0.73-6.57	Calculated	Crude	n/a	n/a	0.779	0.561	

Kang CI, 2010 (51)	6	40	39	516	**2.99**	**1.01-8.84**	Extracted	Adjusted	Adjusted for severe sepsis, higher Pitt bacteremia score, primary bacteremia, pneumonia, and underlying liver disease	n/a	1.095	0.553	
Kang CI, 2011 (52)	3	29	19	175	0.95	0.28-3.23	Calculated	Crude	n/a	n/a	-0.051	0.624	
Kang CI, 2012 (71)	8	108	2	100	3.92	0.81-18.92	Calculated	Crude	n/a	n/a	1.366	0.804	
Kim SH, 2013 (73)	1	15	3	72	1.64	0.16-16.97	Calculated	Crude	n/a	n/a	0.495	1.190	
Ma J, 2017 (48)	15	70	6	43	1.68	0.61-4.58	Calculated	Crude	n/a	n/a	0.519	0.514	
Martelius T, 2016 (56)	26	182	243	2035	1.23	0.8-1.9	Calculated	Crude	n/a	n/a	0.207	0.221	
Melzer M, 2007 (65)	28	46	73	308	**3.57**	**1.48-8.6**	Extracted	Adjusted	Adjusted for age, sex, site of infection, hospital or community-acquired infection, presence in ICU, hypotension at time of bacteremia, malignancy, and neutropenia	n/a	1.273	0.449	
Ortega M, 2009 (58)	33	211	407	4547	**1.89**	**1.29-2.77**	Calculated	Crude	n/a	n/a	0.637	0.195	
Park SH, 2011 (94)	9	50	8	100	6.4	0.3-145.5	Extracted	Adjusted	Adjusted for severe sepsis or septic shock, malignancy, and severity of illness (APACHE II score)	Matched on date diagnosis	1.856	1.578	
Pena C, 2008 (66)	25	100	11	100	**2.7**	**1.26-5.77**	Calculated	Crude	n/a	Matched on site of infection, and date of admission	0.993	0.388	
Trecarichi EM, 2009 (59)	11	26	2	36	**8.84**	**1.48-52.91**	Extracted	Adjusted	Adjusted for inadequate initial antimicrobial therapy and prolonged neutropenia	n/a	2.179	0.912	
Yoon EJ, 2018 (61)	67	524	74	968	**1.77**	**1.25-2.51**	Calculated	Crude	n/a	n/a	0.571	0.178	
**8b) Quinolone resistance**													
Abernethy JK, 2015 (50)	797	3647	2853	16828	**1.37**	**1.25-1.5**	Extracted	Crude	n/a	n/a	0.315	0.047	
Camins BC, 2011 (78)	24	93	7	93	**3.9**	**1.5-10.2**	Extracted	Adjusted	Conditional and adjusted for cirrhosis, cardiac dysfunction, and female gender	Match on year of diagnosis	1.361	0.489	

Cheong HS, 2007 (60)	22	132	47	376	1.4	0.81-2.42	Calculated	Crude	n/a	n/a	0.336	0.279	
Eom JS, 2002 (77)	7	60	2	80	**5.15**	**1.03-25.76**	Calculated	Crude	n/a	n/a	1.639	0.821	
Huotari K, 2003 (54)	2.40%	51	5.50%	102	**nr**	**nr**	n/a	n/a	n/a	Matched on same type of infection	n/a	n/a	Excluded from MA because the numbers for mortality don't multiply to a whole number. Mortality was reported in percentages.
Kim J, 2014 (79)	11	26	23	56	1.05	0.42-2.67	Calculated	Crude	n/a	n/a	0.049	0.472	
Ortega M, 2009 (58)	161	1300	279	3458	**1.61**	**1.31-1.98**	Calculated	Crude	n/a	n/a	0.476	0.105	
Trecarichi EM, 2009 (59)	12	39	1	23	**9.78**	**1.18-81.15**	Extracted	Crude	n/a	n/a	2.280	1.079	
Yoon EJ, 2018 (61)	69	590	72	902	**1.53**	**1.08-2.16**	Calculated	Crude	n/a	n/a	0.425	0.177	
**8c) MDR**													
Cheong HS, 2007 (60)	26	137	43	371	1.81	0.73-4.48	Extracted	Adjusted	Adjusted for CCI, UTI, presentation with acute renal failure, Pitt bacteremia score, and healthcare-associated.	n/a	0.593	0.463	
Lim C, 2016 (45)	645	1717	697	2562	**1.61**	**1.41-1.83**	Calculated	Crude	n/a	n/a	0.476	0.067	
Parveen A, 2015 (39)	44	98	36	129	1.85	0.89-3.86	Extracted	Adjusted	Adjusted for gender, hematological malignancy, hospitalization within 30 days prior to BSI, admission to ICU, previous chemo within 30 day, previous sx with 30 day, previous radiation within 30 day, neutropenia (<100 cells/mm^3), co-morbidities, effectiveness of antimicrobial therapy, and age.	n/a	0.615	0.374	

Yoon EJ, 2018 (61)	97	865	44	627	**1.67**	**1.16-2.42**	Calculated	Crude	n/a	n/a	0.513	0.188	Study used multivariable cox proportional hazard regression to report HR 1.59; 95% CI 1.1-2.29; adjusted for critically ill, polymicrobial infection, primary infection of urinary tract, primary infection of peritoneum, moderate to severe kidney disease, chronic liver disease, WBC, and sfa/foc for adhesion
