# Supplementary material for: Evaluation of the health and healthcare system burden due to antimicrobial-resistant Escherichia coli infections in humans: a systematic review and meta-analysis
Source: Antimicrob Resist Infect Control. 2020 Dec 10;9:200. doi: 10.1186/s13756-020-00863-x (PMC7726913; doi:10.1186/s13756-020-00863-x)
Supplement: Supplementary file 9 — Additional file 9: Funnel plot of the results from the random-effects meta-analysis assessing the impact of third-generation cephalosporin-resistant E. coli infections on 30-day mortality [file 13756_2020_863_MOESM9_ESM.pdf]

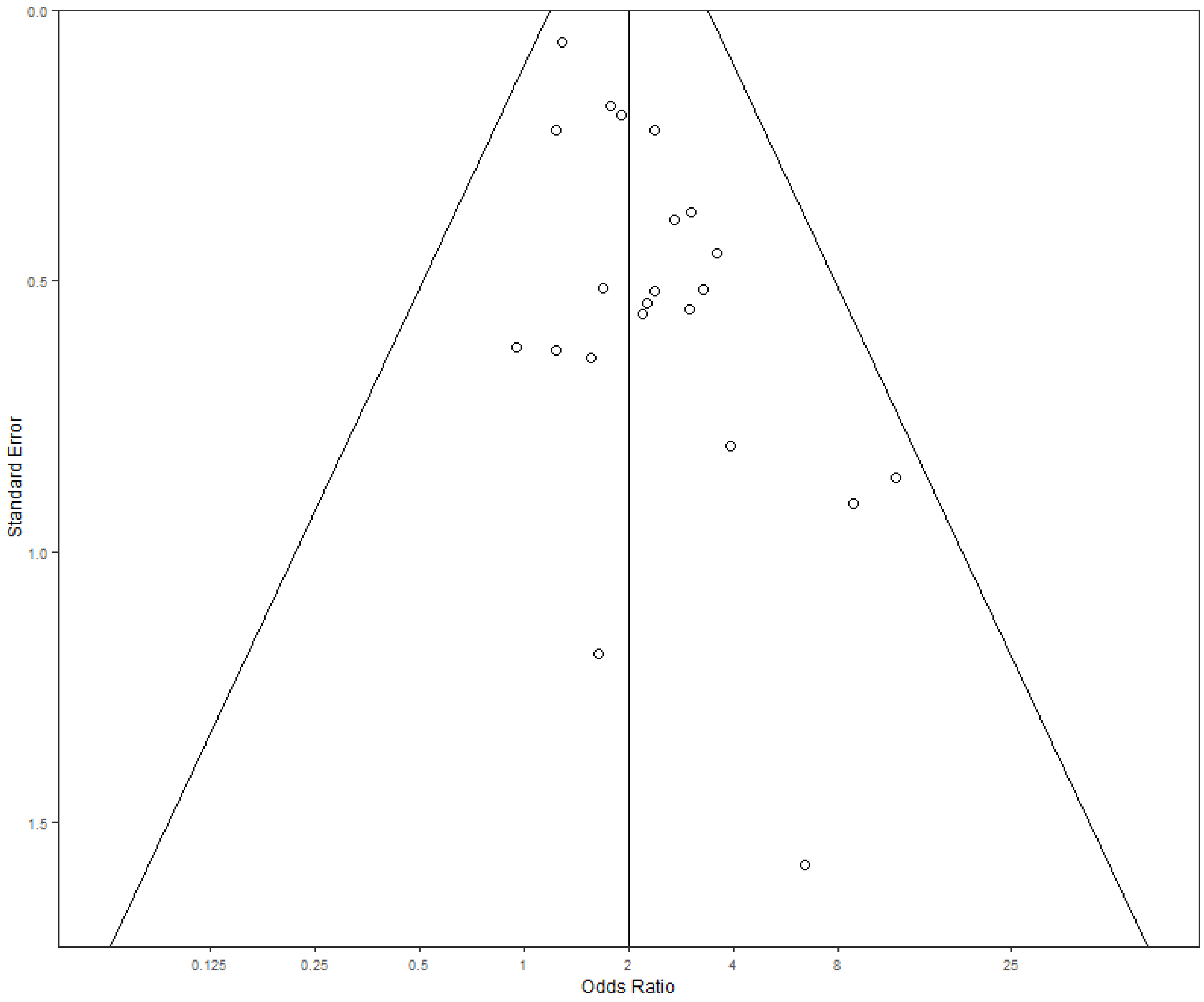

Additional file 9 – Funnel plot of the results from the random-effects meta-analysis assessing the impact of third-generation cephalosporin-resistant *E. coli* infections on 30-day mortality
