# Supplementary material for: Evaluation of the health and healthcare system burden due to antimicrobial-resistant Escherichia coli infections in humans: a systematic review and meta-analysis
Source: Antimicrob Resist Infect Control. 2020 Dec 10;9:200. doi: 10.1186/s13756-020-00863-x (PMC7726913; doi:10.1186/s13756-020-00863-x)
Supplement: Supplementary file 10 — Additional file 10: Forest plot for the random-effects meta-analysis assessing the impacts of MDR E. coli infections on 30-day mortality [file 13756_2020_863_MOESM10_ESM.pdf]

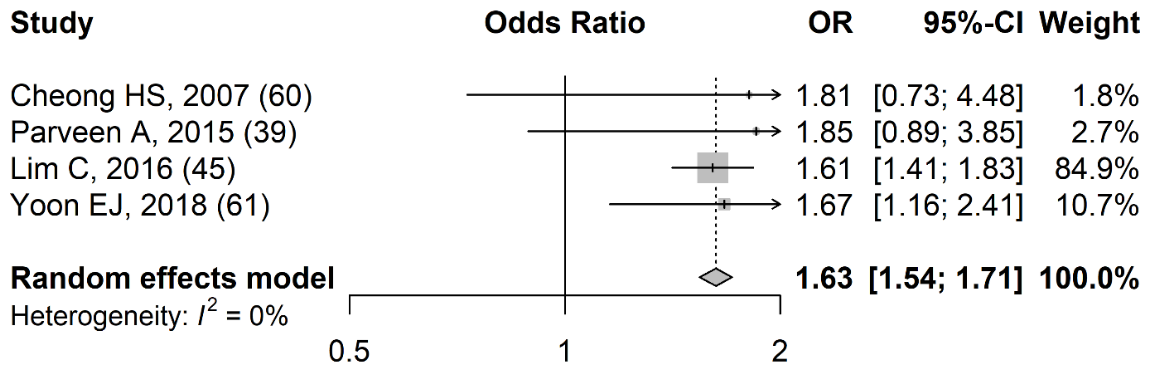

Additional file 10: Forest plot summarizing results from the random-effects meta-analysis assessing the impact of multidrug-resistant *E. coli* infections on 30-day mortality.
