# Supplementary material for: Evaluation of the health and healthcare system burden due to antimicrobial-resistant Escherichia coli infections in humans: a systematic review and meta-analysis
Source: Antimicrob Resist Infect Control. 2020 Dec 10;9:200. doi: 10.1186/s13756-020-00863-x (PMC7726913; doi:10.1186/s13756-020-00863-x)
Supplement: Supplementary file 11 — Additional file 11: Results for all-cause mortality and the type of antimicrobial resistance of interest for the systematic review [file 13756_2020_863_MOESM11_ESM.pdf]

Additional file 11 - Results for all-cause mortality and the type of resistance of interest for a systematic review evaluating whether the measures of health or healthcare system burden increase in humans with antimicrobial-resistant *E. coli* infections.

Citation (Reference # in manuscript)	All-cause definition	Deaths in resistant (R) group	Total in R	Deaths in susceptible (S) group	Total in S	Odds ratio (OR)	95% Confidence interval	OR calculated from raw data or extracted from study	Crude or adjusted	Details of multivariable logistic regression	Details of matching	β (coefficient)	Standard error	Comments
**11a) Third-generation cephalosporin resistance**														
Abernethy JK, 2015 (49)	30-day all cause	392	1838	2911	16641	**1.28**	**1.14-1.44**	Extracted	Crude	n/a	n/a	0.247	0.060	
Al-Otaibi FE, 2013 (86)	deaths (no definition)	10	113	2	226	**10.87**	**2.34-50.52**	Calculated	Crude	n/a	n/a	2.386	0.784	
Alvarez-Uria G, 2012 (37)	21-day all cause	24	154	2	40	**8.24**	**1.19-57.28**	Extracted	Adjusted	Adjusted for sex, HIV infection, isolation from a normally sterile site, and age	n/a	2.109	0.988	
Anunnatsiri S, 2012 (42)	overall in-hospital mortality	9	32	13	113	**7.76**	**1.84-32.72**	Extracted	Adjusted	Adjusted for APACHE II score (severity score at time of culture or within 24 hr), primary septicemia, and secondary septicemia from non-urinary source	n/a	2.049	0.734	
Apisarnthana-rak A, 2008 (43)	in-hospital crude mortality	14	46	9	138	**6.27**	**2.54-15.48**	Calculated	Crude	n/a	n/a	1.836	0.461	
Artero A, 2017 (95)	in-hospital all-cause	11	85	17	225	1.82	0.83-4.00	Calculated	Crude	n/a	n/a	0.599	0.401	
Chakraborty A, 2012 (38)	expired (no definition)	21	132	1	67	**12.49**	**1.64-94.99**	Calculated	Crude	n/a	n/a	2.525	1.035	
Cheong HS, 2007 (60)	30-day	5	19	64	489	2.37	0.86-6.57	Calculated	Crude	n/a	n/a	0.863	0.519	
Cornejo-Juarez P, 2012 (44)	60-day	51	88	51	88	1	0.55-1.82	Calculated	Crude	n/a	Matching details not provided	0.000	0.305	

Courpon-Claudinon A, 2011 (85)	in-hospital	12	39	124	1012	**3.18**	**1.59-6.37**	Calculated	Crude	n/a	n/a	1.157	0.354	
de Kraker ME, 2011 (49)	In-hospital	39	109	190	1101	**2.67**	**1.76-4.06**	Calculated	Crude	n/a	n/a	0.982	0.213	
Denis B, 2015 (74)	30-day	12	41	11	41	1.23	0.36-4.23	Extracted	Adjusted	Adjusted for APACHE II score (severity)	Matched on date of diagnosis	0.207	0.629	
Esteve-Palau E, 2015 (75)	30-day	6	60	4	60	1.56	0.44-5.43	Calculated	Crude	n/a	Matched on age, sex, and date of admission	0.445	0.641	
Freeman JT, 2012 (70)	30-day	10	16	2	16	**11.67**	**2.1-61.93**	Calculated	Crude	n/a	Matched on species of organism, type of blood culture, timing of bacteremia, and data of diagnosis	2.457	0.863	Additionally reported 8-day mortality
Gudiol C, 2010 (67)	30-day	6	17	23	118	2.25	0.78-6.54	Calculated	Crude	n/a	n/a	0.811	0.542	The outcomes are presented for episodes not patients and additionally reported 7-day mortality
Ha YE, 2013 (72)	30-day	21	95	31	255	**3.01**	**1.45-6.28**	Extracted	Adjusted	Adjusted for septic shock, mechanical ventilation, source from UTI, source from intra-abdominal, source from respiratory, and CCI	n/a	1.102	0.374	
Haruki Y, 2018 (76)	28-day	9	24	12	77	**3.25**	**1.18-8.96**	Calculated	Crude	n/a	n/a	1.179	0.517	
Henshke-Bar-Meir R, 2006 (80)	in-hospital	10	39	14	119	**2.59**	**1.06-6.33**	Calculated	Crude	n/a	n/a	0.952	0.456	
Ho PL, 2002 (64)	30-day	9	50	7	100	**2.92**	**1.05-8.10**	Calculated	Crude	n/a	Matched on specialty, age (+/- 10 yr), sex and date of isolation	1.072	0.521	The outcomes are presented for episodes not patients.

Hsieh CJ, 2010 (68)	30-day	4	19	42	385	2.18	0.73-6.57	Calculated	Crude	n/a	n/a	0.779	0.561	Additionally reported 3-day, 15-day and crude mortality.
Huang YY, 2018 (97)	in-hospital	7	58	4	118	**3.91**	**1.16-13.07**	Calculated	Crude	n/a	n/a	1.364	0.618	
Kang CI, 2010 (51)	30-day	6	40	36	516	**2.99**	**1.01-8.84**	Extracted	Adjusted	Adjusted for severe sepsis, higher Pitt bacteremia score, primary bacteremia, pneumonia, and underlying liver disease	n/a	1.095	0.553	
Kang CI, 2011 (52)	30-day	3	29	19	175	0.95	0.28-3.23	Calculated	Crude	n/a	n/a	-0.051	0.624	
Kang CI, 2012 (71)	30-day	8	108	2	100	3.92	0.81-18.92	Calculated	Crude	n/a	n/a	1.366	0.804	
Kaya O, 2013 (46)	in-hospital	16	44	19	69	1.5	0.67-3.38	Extracted	Crude	n/a	n/a	0.405	0.413	
Khan FY, 2010 (82)	in-hospital	14	27	9	70	**7.3**	**2.65-20.14**	Calculated	Crude	n/a	n/a	1.988	0.517	
Kim SH, 2013 (73)	7-day	0*	15*	1*	72*	1.54	0.06-39.55	Calculated	Crude	n/a	n/a	0.432	1.656	*Continuity correction added 0.5 to all cells of 2X2 table
Komatsu Y, 2018 (98)	14-day	6	30	4	85	**4.97**	**1.08-26.01**	Extracted	Crude	n/a	n/a	1.603	0.812	
Lambert ML, 2011 (53)	in ICU	21	42	86	217	1.52	0.79-2.94	Calculated	Crude	n/a	n/a	0.419	0.335	
Lee H, 2018 (99)	in-hospital	0*	50*	2*	100*	0.39	0.018-8.28	Calculated	Crude	n/a	Matched on sex and age (+/- 5 years)	-0.942	1.564	*Continuity correction added 0.5 to all cells of 2X2 table
Lee S, 2014 (89)	14-day	0*	26*	0*	52*	1.98	0.04-102.6	Calculated	Crude	n/a	Matched on bacteremia, age, sex, CCI, SAPS II and modified APN score	0.683	2.002	*Continuity correction added 0.5 to all cells of 2X2 table
Leistner R, 2014 (90)	in-hospital	30	115	181	983	1.56	1.00-2.44	Calculated	Crude	n/a	n/a	0.445	0.228	
Leistner R, 2014 (91)	In-hospital	45	178	233	1321	**1.58**	**1.10-2.28**	Calculated	Crude	n/a	n/a	0.457	0.186	
Ma J, 2017 (48)	30-day	15	70	6	43	1.68	0.61-4.58	Calculated	Crude	n/a	n/a	0.519	0.514	

Martelius T, 2016 (56)	7-day	14	182	121	2035	1.32	0.75-2.33	Calculated	Crude	n/a	n/a	0.278	0.289	
Maslikowska JA, 2016 (93)	All-cause deaths upto and including 14-days after completion of antibiotic therapy	9	61	1	49	**8.31**	**1.01-68.04**	Calculated	Crude	n/a	n/a	2.117	1.074	
Melzer M, 2007 (65)	30-day	28	46	73	308	**3.57**	**1.48-8.60**	Extracted	Adjusted	Adjusted for age, sex, site of infection, hospital or community-acquired infection, presence in ICU, hypotension at time of bacteremia, malignancy, and neutropenia	n/a	1.273	0.449	
Namikawa H, 2017 (96)	all-cause deaths	3	31	9	98	1.06	0.29-3.91	Calculated	Crude	n/a	n/a	0.058	0.664	
Nicolas-Chanoine MH, 2012 (86)	in-hospital	18	152	11	152	1.72	0.80-3.72	Calculated	Crude	n/a	Matched on time of diagnosis	0.542	0.392	
Nussbaum A, 2013 (88)	in-hospital	9	34	11	66	1.8	0.68-4.8	Calculated	Crude	n/a	Matched on age and gender	0.588	0.499	
Ortega M, 2009 (58)	30-day	33	211	407	4547	**1.89**	**1.29-2.77**	Calculated	Crude	n/a	n/a	0.637	0.195	
Park SH, 2011 (69)	7-day	4	50	6	100	1.36	0.39-4.74	Calculated	Crude	n/a	Matched on data of diagnosis	0.307	0.637	
Park SH, 2015 (94)	death within 4 wks after completion of antibiotic therapy	1	75	4	225	0.75	0.08-6.79	Calculated	Crude	n/a	Matched on month of diagnosis	-0.288	1.133	
Pena C, 2008 (66)	30-day	25	100	11	100	**2.7**	**1.26-5.77**	Calculated	Crude	n/a	Matched on site of infection, and date of admission	0.993	0.388	Addtionally, reported 7-day mortality
Peralta G, 2007 (57)	in-hospital	6	31	30	632	**4.82**	**1.89-12.32**	Calculated	Crude	n/a	n/a	1.573	0.478	

Rodriguez-Bano J, 2010 (83)	14-day	16	98	15	187	**2.24**	**1.07-4.69**	Calculated	Crude	n/a	Matched on month of diagnosis and hospital	0.806	0.377	
Trecarichi EM, 2009 (59)	30-day	11	26	2	36	**8.84**	**1.48-52.91**	Extracted	Adjusted	Adjusted for inadequate initial antimicrobial therapy and prolonged neutropenia	n/a	2.179	0.912	
Tumbarello M, 2010 (84)	21-day	11	37	6	97	**6.42**	**2.23-18.39**	Calculated	Crude	n/a	n/a	1.859	0.538	
Van Aken S, 2014 (92)	14-day	6	67	9	130	1.32	0.47-3.75	Calculated	Crude	n/a	Matched on time period, bacterial species, occurrence of bacteremia, and study location	0.278	0.530	
Yip T, 2006 (81)	mortality (no defintion)	3	11	3	77	**9.25**	**1.81-48.00**	Calculated	Crude	n/a	n/a	2.225	0.836	
Yoon EJ, 2018 (61)	30-day	67	524	74	968	**1.77**	**1.25-2.51**	Calculated	Crude	n/a	n/a	0.571	0.178	
**11b) Quinolone resistance**														
Abernethy JK, 2015 (50)	30-day all-cause	797	3647	2853	16828	**1.37**	**1.25-1.50**	Extracted	Crude	n/a	n/a	0.315	0.047	
Bert F, 2008 (103)	in-hospital all-cause	5	11	33	60	0.68	0.20-2.36	Calculated	Crude	n/a	n/a	-0.386	0.630	The outcomes are presented for episodes not patients.
Camins BC, 2011 (78)	in-hospital 30-day mortality	24	93	7	93	**3.9**	**1.50-10.20**	Extracted	Adjusted	Conditional and adjusted for cirrhosis, cardiac dysfunction, and gender	matched on year of infection	1.361	0.489	
Cereto F, 2003 (102)	in-hospital death during an SBP episode	9	12	15	35	4	0.97-16.01	Calculated	Crude	n/a	n/a	1.386	0.715	
Cereto F, 2008 (104)	in-hospital death during an SBP episode	9	18	9	29	2.22	0.66-7.34	Calculated	Crude	n/a	n/a	0.798	0.615	
Cheong HJ, 2001 (101)	in-hospital	12	40	13	80	2.21	0.91-5.36	Calculated	Crude	n/a	Matched on time	0.793	0.452	

Cheong HS, 2007 (60)	30-day	22	132	47	376	1.4	0.81-2.42	Calculated	Crude	n/a	n/a	0.336	0.279	
Eom JS, 2002 (77)	30-day	7	60	2	80	**5.15**	**1.03-25.76**	Calculated	Crude	n/a	n/a	1.639	0.821	
Garau J, 1999 (100)	in-hospital	20	70	53	502	**3.39**	**1.89-6.09**	Calculated	Crude	n/a	n/a	1.221	0.298	
Huotari K, 2003 (54)	30-day	2.40%	51	5.50%	102	**nr**	**nr**	n/a	n/a	n/a	Matched on same type of infection	n/a	n/a	Excluded from MA because the numbers for mortality don't multiply to a whole number. Mortality was reported in percentages.
Kim J, 2014 (79)	30-day	11	26	23	56	1.05	0.42-2.67	Calculated	Crude	n/a	n/a	0.049	0.472	
Laupland KB, 2008 (105)	in-hospital					**1.8**	**1.20-2.80**	Extracted	Adjusted	Adjusted for cancer, haematological malignancy, source/timing of infection, focus of infection, stroke, chronic lung disease, alcoholism, heart disease, & age	n/a	0.588	0.216	
Ortega M, 2009 (58)	30-day	161	1300	279	3458	**1.61**	**1.31-1.98**	Calculated	Crude	n/a	n/a	0.476	0.105	
Peralta G, 2007 (57)	in-hospital	10	125	26	538	1.71	0.82-3.60	Calculated	Crude	n/a	n/a	0.536	0.377	
Shin J, 2012 (106)	in hosppital	0*	32*	0*	173*	5.27	0.10-273.90	Calculated	Crude	n/a	n/a	1.662	2.019	*Continuity correction added 0.5 to all cells of 2X2 table
Trecarichi EM, 2009 (59)	30-day	12	39	1	23	**9.78**	**1.18-81.15**	Calculated	Crude	n/a	n/a	2.280	1.079	
Yoon EJ, 2018 (61)	30-day	69	590	72	902	**1.53**	**1.08-2.16**	Calculated	Crude	n/a	n/a	0.425	0.177	
**11c) MDR**														
Cheong HS, 2007 (60)	30-day	26	137	43	371	1.81	0.73-4.48	Extracted	Adjusted	Adjusted for CCI, UTI, Presentation with acute renal failure, Pitt bacteremia score, and Healthcare-associated	n/a	0.593	0.463	

Lim C, 2016 (45)	30-day	645	1717	697	2562	**1.61**	**1.41-1.83**	Calculated	Crude	n/a	n/a	0.476	0.067	
Parveen A, 2015 (39)	30-day	44	98	36	129	1.85	0.89-3.86	Extracted	Adjusted	Adjusted for gender, hematological malignancy, hospitalization within 30 days prior to BSI, admission to ICU, previous chemo within 30 day, previous sx with 30 day, previous radiation within 30 day, neutropenia (<100 cells/mm3), co-morbidities, effectiveness of antimicrobial therapy, and age	n/a	0.615	0.374	
Uzodi AS, 2017 (62)	death (no definition)	0*	34*	1*	274*	2.64	0.11-66.15	Calculated	Crude	n/a	n/a	0.971	1.632	*Continuity correction added 0.5 to all cells of 2X2 table
Yoon EJ, 2018 (61)	30-day	97	865	44	627	**1.67**	**1.16-2.42**	Extracted	Adjusted	Adjusted for critically ill, polymicrobial infection, primary infection of urinary tract, primary infection of peritoneum, moderate to severe kidney disease, chronic liver disease, WBC, and sfa/foc for adhesion	n/a	0.513	0.188	Study used multivariable cox proportional hazard regression to report HR 1.59; 95% CI 1.1-2.29; adjusted for critically ill, polymicrobial infection, primary infection of urinary tract, primary infection of peritoneum, moderate to severe kidney disease, chronic liver disease, WBC, and sfa/foc for adhesion
