# Supplementary material for: Evaluation of the health and healthcare system burden due to antimicrobial-resistant Escherichia coli infections in humans: a systematic review and meta-analysis
Source: Antimicrob Resist Infect Control. 2020 Dec 10;9:200. doi: 10.1186/s13756-020-00863-x (PMC7726913; doi:10.1186/s13756-020-00863-x)
Supplement: Supplementary file 15 — Additional file 15: Results for bacterium-attributable mortality and third-generation cephalosporin-resistant E. coli infections for the systematic review [file 13756_2020_863_MOESM15_ESM.pdf]

Additional file 15 - Results for bacterium-attributable mortality and third-generation cephalosporin resistance for a systematic review evaluating whether the measures of health or healthcare system burden increase in humans with antimicrobial-resistant *E. coli* infections.

Citation (Reference # in manuscript)	Deaths in resistant (R) group	Total in R	Deaths in susceptible (S) group	Total in S	Odds Ratio (OR)	95% Confidence Ratio	OR calculated from raw data or extracted from study	Crude or adjusted OR	Details of multivariable logistic regression	Details of matching	β (Coefficient)	Standard error	Comments
Cornejo-Juarez P, 2012 (44)	34	71	19	56	1.79	0.87-3.67	Calculated	Crude	n/a	Matched on date of culture	0.582	0.367	
Feng XR, 2014 (47)	0.5	33	1.5	59	0.59	0.02-14.90	Calculated	Crude	n/a	n/a	-0.528	1.687	The outcomes are presented for episodes not patients.
Maslikowska JA, 2016 (93)	3	61	1	49	2.48	0.25-24.65	Calculated	Crude	n/a	Matched on sex, age +/- 5 yr, type of infection, and bed allocation	0.908	1.171	
