# Supplementary material for: Evaluation of the health and healthcare system burden due to antimicrobial-resistant Escherichia coli infections in humans: a systematic review and meta-analysis
Source: Antimicrob Resist Infect Control. 2020 Dec 10;9:200. doi: 10.1186/s13756-020-00863-x (PMC7726913; doi:10.1186/s13756-020-00863-x)
Supplement: Supplementary file 17 — Additional file 17: Results for treatment failure and the type of antimicrobial resistance of interest for the systematic review [file 13756_2020_863_MOESM17_ESM.pdf]

Additional file 17 - Results for treatment failure and the type of resistance of interest for a systematic review evaluating whether the measures of health or healthcare system burden increase in humans with antimicrobial-resistant *E. coli* infections.

**Citation (Reference # in manuscript)**	**Term used to represent treatment failure**	**Definition of treatment failure**	**Lost to follow-up in each group**	**Result in antimicrobial resistant (R) group [number with treatment failure / total in group (%)]**	**Result in antimicrobial susceptible (S) group [number with treatment failure / total in group (%)]**	**Comparison of significance between groups (R vs S)**	**Comments**
**17a) Third-generation cephalosporin resistance**							
Anunnatsiri S, 2012. (42)	Failure	"Absence of improvement or worsening of sepsis" at 72 hours after the start of treatment (only assessed in patients that survived to 72 hours).	R - 3/32; S - 8/113 (all died within 72 hours)	7/29 (24.1%)	6/105 (5.7%)	p=0.01	
Bollestad M, 2018. (107)	Treatment failure (persistent symptoms (a) and second antibiotic prescription (b))	"Persistent symptoms leading to a second antibiotic prescription during the follow-up" period of up to two weeks after completion of initial antibiotic treatment	R (a) - 1/88 (b) none; S (a) & (b) - 2/74	(a) 32/87 (36.8%); (b) 30/88 (34.1%)	(a) 11/72 (15.3%); (b) 10/72 (13.9%)	(a) p=0.01; (b) p=0.01; univariable logistic regression model	
Can F, 2015. (41)	Treatment failure	Persistent symptoms 10 days after start of treatment or detection of resistance to initial/empiric antibiotic and switching to the appropriate antimicrobial.	none	17/70 (24.3%)	18/224 (8.0%)	OR 2.2; 95% CI 1.14-4.4; p=0.02; univariable logistic regression	
Chakraborty A, 2012. (38)	Failure to improve with treatment	Included both relapses and death (there was no explicit definition of relapse or death).	R - 5/132; S - 2/68	41/127 (32.3%)	14/66 (21.2%)	nr	
Chauhan S, 2015 (40)	(a) Clinical failure; (b) Microbiological failure	(a) Persistance or worsening of any clinical parameter. (b) Persistent bacterial growth on culture after 3 days or more of antimicrobial therapy	R (a) - 18/85 (b) - 36/85; S (a) - 11/85 (b) - 54/85	(a) 3/67 (4.5%); (b) 0/49 (0.0%)	(a) 3/74 (4.1%); (b) 3/31 (9.7%)	nr	
Esteve-Palau E, 2015. (75)	Clinical failure	"No improvement or at least one of the initial symptoms worsened, needed a switch of antimicrobial therapy or died" within 7 days of diagnosis.	none	24/60 (40.0%)	4/60 (7.0%)	OR 8.01; 95% CI 2.48 - 25.92; p = 0.001; multivariable logistic regression adjusted for acute pyelonephritis	
Fan NC, 2014. (108)	Early treatment failure	"Fever persisted for more than three days despite treating with antibiotics and antibiotics would be changed within 3-5 days to improve the above clinical conditions."	none	19/104 (18.3%)	14/208 (6.7%)	p = 0.003; $\chi 2$ test	

Feng XR, 2014. (47)	Treatment failure	"Discontinuation of peritoneal dialysis, whether temporary or permanent, and death during peritonitis"	none	2/32 (6.3%)	7/58 (12.1%)	p = 0.31; χ2 test	Outcomes are presented for episodes not patients.
Kang CI, 2010. (51)	Failure	"No improvement or worsening of any of their clinical parameters as well as those who died" within 7 days of starting treatment.	R - 42/82; S - 267/783	8/40 (20.0%)	60/516 (11.6%)	p=0.13; χ^2 test	
Lee S, 2014. (89)	Microbiological failure	Bacterial growth on follow-up urine culture (taken within 5 days after antibiotic treatment started).	R - 4/26; S - 7/52	5/22 (23.0%)	0/45 (0.0%)	p=0.01; McNemar's test	
Maslikowska JA, 2016. (93)	Clinical failure (a) and microbiological failure (b)	(a) "Absence of chart documented resolution of signs and symptoms of infection at the end of and 14 days following discontinuation of antibiotics." (b) "Failure to achieve negative source culture(s) by the end of therapy and 14 days after discontinuation of antibiotics."	R (a) - 28/61 (b) - 49/61; S (a) - 29/49 (b) - 46/49	(a) 13/33 (39.0%); (b) 1/12 (8.3%)	(a) 3/20 (15.0%); (b) 0/3 (0.0%)	(a) p = 0.0728; (b) p > 0.9999; Fisher's exact test	
Park SH, 2015. (94)	Clinical failure (a) and microbiological failure (b)	(a) After initial improvement, either recurrence of symptoms or death during follow-up period of 4 weeks after completing antibiotic therapy. (b) Persistence or recurrence of bacterial growth on culture during the follow-up period of 4 weeks after completing antibiotic therapy.	R (a) & (b) - none; S (a) - none (b) - 48/225	(a) 4/75 (5.3%); (b) 9/75 (12.0%)	(a) 20/225 (8.9%); (b) 26/177 (14.7%)	(a) HR 0.69; 95% CI 0.24-2.02; p = 0.50 (univariable) HR 0.39; 95% CI 0.12-1.30; p = 0.13 (multivariable, adjusted for septic shock, immunocompromise, delay in appropriate treatment, age > 65 yr and antibiotic use within the previous year) (b) HR 0.89; 95% CI 0.41-1.89; p = 0.75 (univariable) HR 0.49; 95% CI 0.21-1.12; p = 0.091 (multivariable, adjusted for antibiotic use with the previous year, chronic kidney disease, diabetes, age > 65 year, concomitant BSI, urinary tract abnormality); multivariable Cox proportional hazards regression model.	
Soraas A, 2014. (109)	Treatment failure	Prescribing a second antibiotic (appropriate for UTI) within 1-14 days after diagnosis	none	43/81 (53%)	58/262 (22.0%)	p < 0.001; χ2 test	
Tumbarello M, 2010. (84)	Treatment failure	Persistent or worsening signs of the infection or death within the first 72 hours	none	17/37 (45.9%)	11/97 (11.3%)	OR = 3.2; 95% CI 1.96-5.27; p < 0.001	

Yip T, 2006. (81)	Treatment failure	Discontinuation of peritoneal dialysis or death during peritonitis espisode	none	5/11 (45.5%)	10/77 (13.0%)	p = 0.02; $\chi 2$ test	
**17b) Quinolone resistance**							
Camins BC, 2011. (78)	Clinical failure	If not discharged or died within 30 days of diagnosis, recurrent infection within 30 days or a positive blood culture for *E. coli* within 14 days after starting antimicrobial treatment.	none	26/93 (28.0%)	8/93 (9.0%)	p = 0.001; Mantel-Haenszel $\chi 2$ test	
Can F, 2015. (41)	Treatment failure	Persistent symptoms 10 days after start of treatment or detection of resistance to initial/empiric antibiotic and switching to the appropriate choice.	none	28/114 (24.6%)	7/180 (3.9%)	OR 3.1; 95% CI 1.62-6.02; p = 0.001; univariable logistic regression	
Cereto F, 2008. (104)	Treatment failure	Persistance of spontaneous bacterial peritonitis after 6 days of antibiotic therapy.	none	4/18 (22.2%)	6/29 (20.7%)	p = 1; $\chi 2$ test	
Eom JS, 2002. (77)	Fail-to-treat	Bacterial growth on follow-up culture or UTI relapse within 2 weeks of the therapy ending	R - 28/60; S - 27/80	11/34 (32.4%)	6/53 (11.3%)	p=0.02	
Gagliotti C, 2008. (111)	Treatment failure	"Second isolation of *E. coli* from urine between 4 and 30 days after the first isolation."	nr	nr	nr	IRR 1.85; 95% CI 1.32-2.60; p < 0.001; multivariable poisson regression adjusted for prescription of an antibiotic to which E coli is susceptible, prescription of fluoroquinolones, prescription of penicillins-beta-lactamase inhibitors, prescription of penicillins with extended spectrum	
Jeon JH, 2012. (110)	(a) Clinical failure; (b) Microbiological failure	(a) Persistance or worsening of signs and symptoms, or recurrence after cure or improvement. (b) Identification of infection with a new pathogen or persistence of the original pathogen from culture during a follow-up visit (first (1st) - 4-7 days after starting treatment and second (2nd) - 14-21 days after finishing treatment)	1st R (a) none (b) 3/39; S (a) none (b) 18/216; 2nd R (a) none (b) 15/39; S (a) 7/216 (b) 161/216	1st (a) 9/39 (23.1%) (b) 21/36 (58.3%); 2nd (a) 2/39 (5.1%) (b) 7/24 (29.2%)	1st (a) 1/216 (0.5%) (b) 15/198 (7.6%); 2nd (a) 3/209 (1.4%) (b) 10/55 (18.2%)	1st (a) p=0.135 (b) p<0.001; 2nd (a) p=0.177 (b) p=0.372; $\chi 2$ test	

Shin J, 2012. (106)	Clinical treatment failure	Worsening of any clinical parameter after 3 days of treatment, failure to improve, or recurrence of symptoms within 4-10 days after completion of treatment.	none	7/32 (21.9%)	15/173 (8.7%)	OR 2.94; 95% CI 1.03-8.37; p = 0.04; multivariable logistic regression adjusted for age, antibiotic use before visit and presence of bacteremia	
**17c) MDR**							
Can F, 2015. (41)	Treatment failure	Persistent symptoms 10 days after start of treatment or detection of resistance to initial/empiric antibiotic and switching to the appropriate choice.	none	30/116 (25.9%)	5/178 (2.8%)	OR 2.9; 95% CI 1.65-5.3; p < 0.001; univariable logistic regression	
Uzodi AS, 2017. (62)	Cure with recurrence	Recurrence after cure (excluding death)	R - none S - 11/284	12/34 (35.0%)	55/273 (20.1%)	p > 0.05	
