# Supplementary material for: Evaluation of the health and healthcare system burden due to antimicrobial-resistant Escherichia coli infections in humans: a systematic review and meta-analysis
Source: Antimicrob Resist Infect Control. 2020 Dec 10;9:200. doi: 10.1186/s13756-020-00863-x (PMC7726913; doi:10.1186/s13756-020-00863-x)
Supplement: Supplementary file 18 — Additional file 18: Results for total length of hospital stay and the type of antimicrobial resistance of interest for the systematic review [file 13756_2020_863_MOESM18_ESM.pdf]

Additional file 18 - Results for total length of hospital stay and the type of resistance of interest for a systematic review evaluating whether the measures of health or healthcare system burden increase in humans with antimicrobial-resistant *E. coli* infections.

Citation (Reference # in manuscript)	Mean LOS in resistant (R) group in days	SD of LOS in R group	Total in R	Mean LOS in susceptible (S) group in days	SD of LOS in S group	Total in S	Alternate LOS raw data	Details of matching	Comments
**18a) Third-generation cephalosporin resistance**									
Apisarnthanarak A, 2008 (43)	nr	nr	46	nr	nr	138	median days (range) R - 8 (1-43); S - 6.5 (3-15)	Matched on site of infection, hospital unit and day of admission (+/- 7 days).	
Artero A, 2017 (95)	6.6	3.69	85	5.61	3.16	225	n/a	n/a	
Chauhan S, 2015 (40)	40.22	20.26	85	14	7.4	85	n/a	Matched on time	
Denis B, 2015 (74)	nr	nr	41	nr	nr	41	median days (IQR) R - 15 (10-21); S - 11 (7-17)	Matched on date of culture	
Esteve-Palau E, 2015 (75)	11.6	1.5	60	7.5	0.8	60	n/a	Matched on sex, age, and date of admission.	
Fan NC, 2014 (108)	nr	nr	104	nr	nr	208	days R - 12.08; S - 6.88	Matched on age and sex	Did not specify if LOS reported is a mean or median and no measure of variability
Huang YY, 2018 (97)	nr	nr	58	nr	nr	118	median days (IQR) R - 11 (6-27); S - 7 (3-13)	n/a	
Lee H, 2018 (99)	nr	nr	50	nr	nr	100	median days (IQR) R - 13.5 (7-21); S - 7 (3-12)	Matched on sex and age (+/- 5yr)	
Lee S, 2014 (89)	13.3	8.2	26	7.3	3.5	52	n/a	Matched on bacteremia, age, sex, CCI, SAPS II and modified APN score.	
Leistner R, 2014 (90)	nr	nr	115	nr	nr	983	median days (IQR) R - 27 (12-53); S - 15 (8-32)	n/a	
Nussbaum A, 2013 (88)	13.2	nr	34	13.7	nr	66	median days (range) R - 12.5 (1-39); S - 11 (1-71)	Matched on age and gender	
Park SH, 2015 (94)	nr	nr	75	nr	nr	225	median days (IQR) R - 11 (6-16); S - 7 (6-9)	Matched on time (within a month of each case)	
Van Aken S, 2014 (92)	11	12	70	9	10	140	n/a	Matched for time period, bacterial species, occurrence of bacteremia, and study location	

**18b) Quinolone resistance**									
Camins BC, 2011 (78)	18.2	21.9	93	10.4	10	93	n/a	Matched on year of infection	
Huotari K, 2003 (54)	nr	nr	51	nr	nr	102	days R - 17.7; S - 18.7	Matched on type of infection	Did not specify if LOS reported is a mean or median and no measure of variability
Jeon JH, 2012 (110)	8.2	5	39	7.8	3.1	216	n/a	n/a	
Shin J, 2012 (106)	9.6	5.5	32	7	3.5	173	n/a	n/a	
**18c) MDR**									
Uzodi AS, 2017 (62)	nr	nr	8	nr	nr	35	median days (IQR) R - 10 (4.5-20.5); S - 4 (2-6)	n/a	

LOS - length of hospital stay; SD - standard deviation; nr - not reported
