# Supplementary material for: Evaluation of the health and healthcare system burden due to antimicrobial-resistant Escherichia coli infections in humans: a systematic review and meta-analysis
Source: Antimicrob Resist Infect Control. 2020 Dec 10;9:200. doi: 10.1186/s13756-020-00863-x (PMC7726913; doi:10.1186/s13756-020-00863-x)
Supplement: Supplementary file 19 — Additional file 19: Forest plot for the random-effects meta-analysis assessing the impacts of third-generation cephalosporin-resistant E. coli infections on total length of hospital stay [file 13756_2020_863_MOESM19_ESM.pdf]

Study	Resistant Total	Resistant Mean	Resistant SD	Susceptible Total	Susceptible Mean	Susceptible SD	MD	95%-CI
Lee S, 2014 (89)	26	13.30	8.20	52	7.30	3.50	6.00	[2.71; 9.29]
Van Aken S, 2014 (92)	70	11.00	12.00	140	9.00	10.00	2.00	[-1.26; 5.26]
Esteve-Palau E, 2015 (75)	60	11.60	1.50	60	7.50	0.80	4.10	[3.67; 4.53]
Chauhan S, 2015 (40)	85	40.22	20.26	85	14.00	7.40	26.22	[21.63; 30.81]
Artero A, 2017 (95)	85	6.60	3.69	225	5.61	3.16	0.99	[0.10; 1.88]

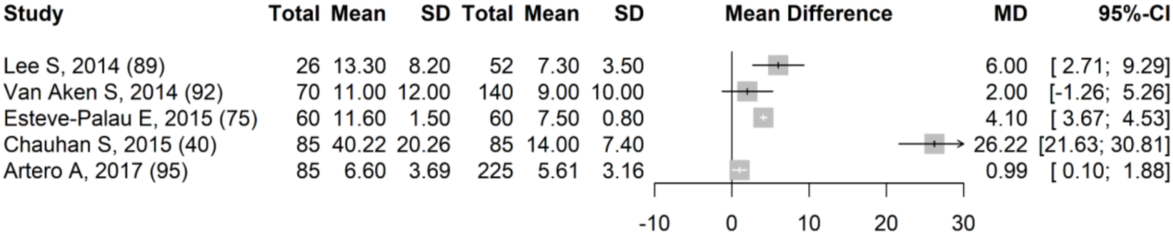

Additional file 19: Forest plot summarizing results from the random-effects meta-analysis assessing the impact of third-generation cephalosporin-resistant *E. coli* infections on total length of hospital stay (days).
