# Supplementary material for: Evaluation of the health and healthcare system burden due to antimicrobial-resistant Escherichia coli infections in humans: a systematic review and meta-analysis
Source: Antimicrob Resist Infect Control. 2020 Dec 10;9:200. doi: 10.1186/s13756-020-00863-x (PMC7726913; doi:10.1186/s13756-020-00863-x)
Supplement: Supplementary file 20 — Additional file 20: Forest plot for the random-effects meta-analysis assessing the impacts of quinolone-resistant E. coli infections on total length of hospital stay [file 13756_2020_863_MOESM20_ESM.pdf]

Study	Resistant Total	Resistant Mean	Resistant SD	Susceptible Total	Susceptible Mean	Susceptible SD	MD	95%-CI
Camins BC, 2011 (78)	93	18.20	21.90	93	10.40	10.00	7.80	[2.91; 12.69]
Jeon JH, 2012 (110)	39	8.20	5.00	216	7.80	3.10	0.40	[-1.22; 2.02]
Shin J, 2012 (106)	32	9.60	5.50	173	7.00	3.50	2.60	[0.62; 4.58]

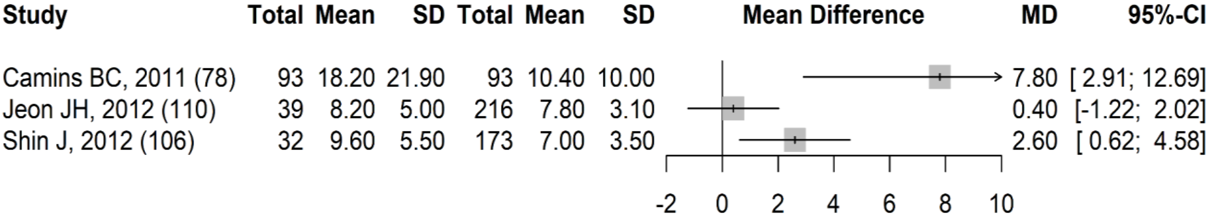

Additional file 20: Forest plot summarizing results from the random-effects meta-analysis assessing the impact of quinolone-resistant *E. coli* infection on total length of hospital stay (days).
