# Supplementary material for: Evaluation of the health and healthcare system burden due to antimicrobial-resistant Escherichia coli infections in humans: a systematic review and meta-analysis
Source: Antimicrob Resist Infect Control. 2020 Dec 10;9:200. doi: 10.1186/s13756-020-00863-x (PMC7726913; doi:10.1186/s13756-020-00863-x)
Supplement: Supplementary file 21 — Additional file 21: Results for post-infection length of hospital stay and third-generation cephalosporin-resistant E. coli infections for the systematic review [file 13756_2020_863_MOESM21_ESM.pdf]

Additional file 21 - Results for post-infection length of hospital stay and third-generation cephalosporin resistance for a systematic review evaluating whether the measures of health or healthcare system burden increase in humans with antimicrobial-resistant *E. coli* infections.

Citation (Reference # in manuscript)	Mean LOS in resistant (R) group in days	SD of LOS in R group	Total in R	Mean LOS in susceptible (S) group in days	SD of LOS in S group	Total in S	Alternate LOS raw data	Details of matching	Comments
Anunnatsiri S, 2012 (42)	nr	nr	29	nr	nr	105	median days (range) R- 13 (3-48); S - 8 (3-102)	n/a	
de Kraker ME, 2011 (49)	nr	nr	109	nr	nr	1101	median days (IQR) R - 12 (6-25); S - 10 (6-17)	n/a	
Hsieh CJ, 2010 (68)	21	27.1	19	13.1	13	385	n/a	n/a	
Lambert ML, 2011 (53)	nr	nr	21	nr	nr	131	median days (IQR) R - 16 (4-24); S - 9 (5-20)	n/a	
Leistner R, 2014 (112)	nr	nr	92	nr	nr	92	median days (IQR) R - 15 (8-32); S - 17 (8-38)	Matching of cases to controls 1:1, matched on age +/- 5 yr, sex, CCI +/- 2, discharge year and the LOS before BSI onset in the control had to be at least as long as the case	
Melzer M, 2007 (65)	nr	nr	18	nr	nr	235	median days R - 9; S - 12	n/a	Does not include any of the patients that died and did not report a measure of varibility
Tumbarello M, 2010 (84)	20	17	37	13	9	97	n/a	n/a	

LOS - length of hospital stay; SD - standard deviation; nr - not reported
