# Supplementary material for: Evaluation of the health and healthcare system burden due to antimicrobial-resistant Escherichia coli infections in humans: a systematic review and meta-analysis
Source: Antimicrob Resist Infect Control. 2020 Dec 10;9:200. doi: 10.1186/s13756-020-00863-x (PMC7726913; doi:10.1186/s13756-020-00863-x)
Supplement: Supplementary file 22 — Additional file 22: Results for healthcare costs and the type of antimicrobial resistance of interest for the systematic review [file 13756_2020_863_MOESM22_ESM.pdf]

**Additional file 22 - Results for healthcare costs and the type of resistance of interest for a systematic review evaluating whether the measures of health or healthcare system burden increase in humans with antimicrobial-resistant *E. coli* infections.**

| Citation (Reference # in manuscript) | Definition of healthcare system cost measure | Mean cost in resistant (R) group | SD for R | Total patients in R | Mean cost in susceptible (S) group | SD for S | Total patients in S | Year, currency | Additional cost related results |
|---|---|---|---|---|---|---|---|---|---|
| **22a) Third-generation cephalosporin resistance** | | | | | | | | | |
| Apisarnthanarak A, 2008. (43) | The costs were "the direct and indirect costs required to provide healthcare services and medications." They were the costs after onset of infection. | nr | nr | 46 | nr | nr | 138 | nr, USD | median (range) R - $528 ($43-$3,173); S - $194 ($53-$1,861); p<0.05 |
| Esteve-Palau E, 2015. (75) | (a) Cost of hospitalization, including costs related to pharmacy (antibiotics and pharmacy costs), laboratory and hospital stay.<br>(b) Cost of parenteral outpatient antibiotic therapy (OPAT).<br>(c) Cost of espisode - combination of (a) and (b) | nr | nr | 60 | nr | nr | 60 | nr, Euros | median (IQR) (a) R - $3,611 ($2,619-$7,141), S - $2,502 ($1,716-$4,218), p=0.007; (b) R - $2,966 ($1,998-$5,254), S - $1,577 ($782-$2,473), p=0.04; (c) R - $4,980 ($2,783-$8,465), S - $2,612 ($1,810-$4,318), p<0.001; OR 3.1; 95% CI 1.3-7.0; p = 0.008; Multivariable logistic regression for cost of episode adjusted for male gender, chronic renal failure and OPAT |
| Leistner R, 2014. (112) | (a) Total costs that included direct and indirect costs. Direct costs included medical products, laboratory and pharmacy costs. Indirect costs included overhead, costs of staffing. (b) Average cost per day. | nr | nr | 92 | nr | nr | 92 | nr, Euros | median (IQR) (a) R - $15,082 ($6,263-$41,488), S - $16,561 ($5,599-$47,028), p=0.36; (b) - R - $696/day ($469-$990/day), S - $658/day ($444-$1,003/day), p=0.69 |
| Tumbarello M, 2010. (84) | Direct health care costs, "total expenditures incurred by the hospital to provide services or goods for each patient with a BSI." This included costs related to medical care, nursing care, pharmacy services, diagnostic testing, support services, other costs (eg utilities, admission/discharge costs, depreciation costs, overhead costs). | $ 13,709 | $ 16,312 | 37 | $ 8,683 | $ 6,683 | 97 | 2006, Euros | p=0.03 |

| | | | | | | | | | |
|---|---|---|---|---|---|---|---|---|---|
| **22b) MDR** | | | | | | | | | |
| Riu M, 2016. (55) | "Cost of the hospitalization episode." | $ 16,754 | nr | 39 | $ 21,883 | nr | 145 | 2012, Euros | n/a |
| Thaden JT, 2017. (63) | "Cost estimates include fixed direct costs and variable direct costs for all medical services received, inclusive of supplies, laboratory tests, drugs, dietary needs," salary and benefits (not physicians), overhead costs and depreciation. Physician fees were estimated using the Medicare physician fee schedule. | $ 18,917 | $ 29,394 | 165 | $ 14,776 | $ 24,416 | 165 | 2015, USD | median (IQR) R - $9,527 ($5,915-$19,648); S - $7503 ($4301-$13447); Means ratio 1.43; 95%CI 1.20-1.71; p<0.0001; multivariable generalized linear model with gamma distribution and log link adjusted for age, race, APACHE II score, gender, 8 different co-morbidities, hospital-acquired vs community-acquired. |

SD - standard deviation; nr - not reported
