# Supplementary material for: Evaluation of the health and healthcare system burden due to antimicrobial-resistant Escherichia coli infections in humans: a systematic review and meta-analysis
Source: Antimicrob Resist Infect Control. 2020 Dec 10;9:200. doi: 10.1186/s13756-020-00863-x (PMC7726913; doi:10.1186/s13756-020-00863-x)
Supplement: Supplementary file 23 — Additional file 23: Detailed results of GRADE assessment of primary outcome for the systematic review [file 13756_2020_863_MOESM23_ESM.pdf]

Additional file 23 - Detailed results of GRADE assessment of primary outcomes for a systematic review evaluating whether the measures of health or healthcare system burden increase in humans with antimicrobial-resistant *E. coli* infections.[a]

Burden of disease measure	Type of antimicrobial resistance	Risk of bias	Inconsistency	Publication bias	Imprecision	Other considerations[b]	Certainty of evidence (GRADE)	Comments
30-day mortality	3GC-R	No strong evidence to downgrade, with ROBINS-I: Serious (11 studies); Moderate (12 studies)	No serious inconsistency, study point estimates generally same only 1 is 0.95 others all >1, 95% CIs overlap, I2 = 49%	No strong evidence to downgrade, minimal publication bias	No serious imprecision, very large sample size and 95% CI of sOR does not include null	Strong association sOR 2.02 (1.66-2.46) evidence to support upgrade	⊕⊕⊕◯ MODERATE	Evidence to support upgrading due to strong association and no evidence to support downgrading
30-day mortality	Quinolone	No strong evidence to downgrade, with ROBINS-I: Serious (5 studies); Moderate (3 studies)	No serious inconsistency, study point estimates generally same all >1, 95% CIs overlap, I2 = 44%	Unable to assess	No serious imprecision, very large sample size (even with only 8 studies) and 95% CI of sOR does not include null		⊕⊕◯◯ LOW	No evidence to support downgrading or upgrading
30-day mortality	MDR	No strong evidence to downgrade, with ROBINS-I: Serious (2 studies); Moderate (2 studies)	No serious inconsistency, study point estimates very similar, 95% CIs overlap, I2 = 0%	Unable to assess	No serious imprecision, large sample size (even with only 4 studies) and 95% CI of sOR does not include null		⊕⊕◯◯ LOW	No evidence to support downgrading or upgrading
All-cause mortality	3GC-R	No strong evidence to downgrade, with ROBINS-I: Critical (1 study); Serious (21 study); Moderate (29 studies)	No serious inconsistency, study point estimates generally same only 4 are ≤ 1 others all >1, 95% CIs overlap, I2 = 56%	No strong evidence to downgrade, minimal publication bias	No serious imprecision, very large sample size and 95% CI of sOR does not include null	Strong association sOR 2.26 (1.90-2.68), evidence to support upgrade	⊕⊕⊕◯ MODERATE	Evidence to support upgrading due to strong association and no evidence to support downgrading
All-cause mortality	Quinolone	No strong evidence to downgrade, with ROBINS-I: Serious (8 studies); Moderate (8 studies)	No serious inconsistency, study point estimates generally same only 1 is 0.68 others all >1, 95% CIs overlap, I2 = 44%	Minimal publication bias, no strong evidence to downgrade	No serious imprecision, very large sample size and 95% CI of sOR does not include null		⊕⊕◯◯ LOW	No evidence to support downgrading or upgrading

All-cause mortality	MDR	No strong evidence to downgrade, with ROBINS-I: Serious (3 studies) Moderate (2 studies)	No serious inconsistency, study point estimates generally very similar, 95% CIs overlap, I2 = 0%	Unable to assess	No serious imprecision, large sample size (even with only 5 studies) and 95% CI of sOR does not include null		⊕⊕◯◯ LOW	No evidence to support downgrading or upgrading
Bacterium-attributable mortality	3GC-R	No strong evidence to downgrade, with ROBINS-I: Serious (2 studies); Moderate (1 study)	Serious inconsistency, study point estimates different	Unable to assess	Serious imprecision, small sample size and 95% CI of sOR include null and is very wide		⊕◯◯◯ VERY LOW	Downgraded due to serious inconsistency and imprecision. No evidence to support upgrading.
LOS	3GC-R	No strong evidence to downgrade, with ROBINS-I: Moderate (5 studies)	Serious inconsistency, one study's point estimates very different and the 95% CI does not overlap, I2 = 97%	Unable to assess	Serious imprecision, small sample size	Unable to assess strength of sMD	⊕◯◯◯ VERY LOW	Downgraded due to serious inconsistency and imprecision.
LOS	Quinolone	No strong evidence to downgrade, with ROBINS-I: Serious (1 study); Moderate (2 studies)	Serious inconsistency, study point estimates different, I2 = 78.3%	Unable to assess	Serious imprecision, small sample size	Unable to assess strength of sMD	⊕◯◯◯ VERY LOW	Downgraded due to serious inconsistency and imprecision.
Post-infection LOS	3GC-R	No strong evidence to downgrade, with ROBINS-I: Moderate (2 studies)	No serious inconsistency, study point estimates are similar, 95% CIs overlap, I2 = 0%	Unable to assess	Serious imprecision, due to small sample size	Strong association sMD 7.16 days (2.76-11.57 days), evidence to support upgrade	⊕◯◯◯ VERY LOW	Downgraded due to serious imprecision. Evidence to support upgrading outweighed by evidence to support downgrading.

3GC-R - third-generation cephalosorin resistance; CI - confidence interval; sOR - summary odds ratio; MDR - multidrug resistance; LOS - length of hospital stay; sMD - summary mean difference

[a] For all of the outcomes and type of AMR there was no serious indirectedness, populations were comparable, exposures were the same and outcomes were direct

[b] Strong association sOR ≥ 2 or MD ≥ 5 days
